# Synthesis and Characterization of New Spirooxindoles Including Triazole and Benzimidazole Pharmacophores via [3+2] Cycloaddition Reaction: An MEDT Study of the Mechanism and Selectivity

**DOI:** 10.3390/molecules28196976

**Published:** 2023-10-08

**Authors:** Saeed Alshahrani, Abdullah Mohammed Al-Majid, Abdullah Saleh Alamary, Mohamed Ali, Mezna Saleh Altowyan, Mar Ríos-Gutiérrez, Sammer Yousuf, Assem Barakat

**Affiliations:** 1 Department of Chemistry, College of Science, King Saud University, P.O. Box 2455, Riyadh 11451, Saudi Arabia; chemistry99y@gmail.com (S.A.); amajid@ksu.edu.sa (A.M.A.-M.); alamary1401@yahoo.com (A.S.A.); maly.c@ksu.edu.sa (M.A.); 2 Department of Chemistry, College of Science, Princess Nourah bint Abdulrahman University, P.O. Box 84428, Riyadh 11671, Saudi Arabia; msaltowyan@pnu.edu.sa; 3 Department of Organic Chemistry, University of Valencia, Dr. Moliner 50, 46100 Burjassot, Valencia, Spain; m.mar.rios@uv.es; 4 H.E.J. Research Institute of Chemistry, International Centre for Chemical and Biological Sciences, University of Karachi, Karachi 75270, Pakistan; dr.sammer.yousuf@gmail.com

**Keywords:** spirooxindoles, benzimidazole, triazoles, molecular electron density theory (MEDT)

## Abstract

A new series of spirooxindoles based on benzimidazole, triazole, and isatin moieties were synthesized via a [3+2] cycloaddition reaction protocol in one step. The single X-ray crystal structure of the intermediate triazole-benzimidazole **4** was solved. The new chemical structures of these spirooxindole molecules have been achieved for the first time. The final synthesized chemical architecture has differently characterized electronic effects. An MEDT study of the key 32CA reaction between in situ generated azomethine ylide (AY) and chalcones explained the low reaction rates and the total selectivities observed. The supernucleophilic character of AY and the strong electrophilicity of chalcones favor these reactions through a highly polar *two-stage one-step* mechanism in which bond formation at the β-conjugated carbon of the chalcones is more advanced. The present combined experimental and theoretical study reports the synthesis of new spirooxindoles with potential biological activities and fully characterizes the molecular mechanisms for their formation through the key 32CA reaction step.

## 1. Introduction

There are excellent moieties in spiro-heterocyclic compounds that have garnered the attention of researchers due to their numerous biological activities. Among these moieties, the 1,2,3-triazole moiety represents an important class of pharmacophore in medicinal chemistry with a wide range of biological activities, such as antimicrobial, anticancer, anti-inflammatory, and antiviral activity, among others. Because of their higher stability toward light, oxygen, moisture, and metabolism in the body, they are useful building blocks in chemistry and play an important role in pharmacological applications [1,2,3,4]. On the other hand, isatin derivatives have recently drawn considerable attention from researchers worldwide due to their wide applications as anti-HIV, anti-tubercular, sedative, hypnotic, and anticancer agents [5,6]. The important biological activities of both isatin and triazole derivatives as discussed above impelled us to take up the synthesis of these new combined heterocycles, which are likely to have augmented, diverse types of biological activity.

**Figure 1:**
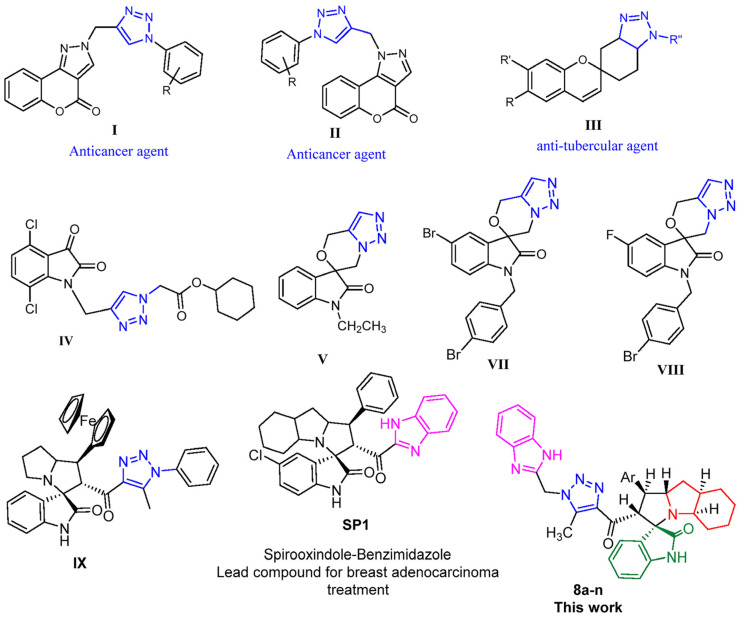
Selective bioactive fused triazoles and spirooxindoles derivatives. Spirooxindole-Benzimidazole Lead compound for breast adenocarcinoma treatment.

To develop potent anticancer candidates, Bin Yu et al. [4] reported, in 2016, a series of new isatin/triazole conjugates with anti-proliferative activity and evaluated their cytotoxic potential against MGC-803 and MCF-7 (breast) cells. Some of these conjugates are shown in Figure 1 (compounds **I**–**III**). These compounds showed selective inhibition toward MGC-803 cells and were less toxic to normal cells HL-7702 and GES-1. Of these compounds, compound **IV** showed the best inhibitory activity against MGC-803 cells (IC_50_ = 9.78 µM), induced apoptosis through multiple mechanisms, and inhibited the migration of MGC-803 cells. 

Senwar et al. [7], in 2015, synthesized a series of new spirooxindole-derived morpholine-fused-1,2,3-triazole derivatives from isatin spiro-epoxides. These compounds were evaluated for their antiproliferative activity against lung (A549), breast (MCF–7), cervical (HeLa), and prostate (DU–145) tumor cell lines. Among the tested compounds, **V–VIII** (see Figure 1) showed potent growth inhibition against the A549 cell line, with IC_50_ values in the range of 1.87–4.36 µM, and decreased migration potential, constituting results that are comparable to those obtained for the reference standards 5-flourouracil and doxorubicin. In another study, Kishore Kumar et al. [8], in 2016, synthesized and developed a new series of 1,2,3-triazole derivatives. The products were tested for their anti-inflammatory activity in vivo. The several tested compounds demonstrated potent anti-inflammatory activity compared to the reference drug ibuprofen [8]. In 2016, Rajeswari et al. [9] developed an efficient, one-pot, four-component condensation procedure for the synthesis of selective spirooxindole-pyrrolizine-linked 1,2,3-triazole conjugates via a [3+2] cycloaddition (32CA) reaction using coumarin-3-carboxylic acid, *N*-propargylated isatin, L-proline/sarcosine, and aryl azides and using Cu(I) as a catalyst in the presence of glacial CH_3_COOH at 60 °C [9]. In 2019, Malarkodi et al. [10] synthesized and compared 3’-(1-benzyl-5-methyl-1*H*-1,2,3-triazole-4carbonyl)-1’methyl-4’-phenyl-2*H*-spiro[acenaphthylene-1,2’-pyrrolidin]-2-one (BTANP) against a few bacterial and fungal strains as well as standard drugs. In addition, molecular docking mockups were developed on BTANP against topoisomerase II gyrase and human lanosterol 14 *α* demethylase enzymes [10].

Recently, Barakat et al. reported the synthesis of new spirooxindoles, **IX**, with the triazole moiety and a ferrocene scaffold using the 32CA reaction approach, and their mechanism was studied via molecular electron density theory (MEDT) [11,12]. Another representative example is the spirooxindole with a benzimidazole scaffold (see **SP1** in Figure 1), which has been extensively studied and has shown to be a potent anti-cancer agent [13]. In continuation of our research program about spirooxindoles [14,15,16], we report herein the synthesis of new spiro compounds containing benzimidazole and 1,2,3–triazole scaffolds as well as the theoretical study of the reaction mechanisms of these relevant 32CA reactions based on MEDT [17].

## 2. Results and Discussion

**Scheme 1:**
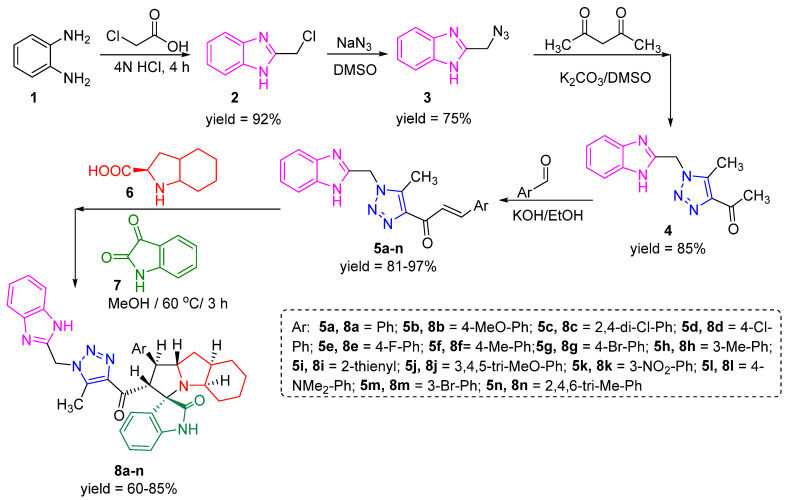
Synthesis of chalcones (**5a-n**) and spiro compounds (**8a-n**).

1,2,3-Triazoles represent an important class of heterocyclic compounds with a wide range of biological activities, constituting useful building blocks in chemistry and pharmacological applications. In this context, an attempt was made to synthesize a novel series of spiro compounds having a triazole nucleus combined with the benzimidazole scaffold, as depicted in Scheme 1.

### 2.1. Synthesis of Chalcones (***5a-n***)

The four steps of the synthesis of the target α,β-unsaturated compounds (**5a-n**) are presented in (Scheme 1). The first step was to synthesize 2-(chloromethyl) benzimidazole (**2**) via the Phillip’s reaction, involving the condensation of *o*-phenylenediamine with chloroacetic acid in the presence of dilute hydrochloric acid. The second step was the reaction of a mixture of 2-(chloromethyl) benzimidazole (1.0 equiv.) and sodium azide (1.1 equiv.) in DMSO (15 mL), followed by stirring at room temperature. The reaction was completed in 3 h, affording 2-(azidomethyl) benzimidazole (**3**) in a 75% yield (^1^HNMR and ^13^CNMR data provided in Supplementary materials; Figures S1 and S2). In the third step, we used the cycloaddition reaction between 2-(azidomethyl) benzimidazole and acetylacetone in DMSO in the presence of an equimolar amount of K_2_CO_3_ at 25 °C (3 h); in this case, the yield of 1,2,3-triazole compound (**4**) was 85%, which is required for preparing chalcone derivatives.

As shown in Scheme 1, in the last step, a mixture of 1,2,3-triazolyl ketone (**4**, 1.0 eq), aromatic aldehydes (1.1 eq) and a 10% solution of KOH in ethanol (20 mL) was stirred at room temperature. The reaction was completed in 5–10 h, affording 1,2,3-triazolyl chalcone derivatives (**5a-n**) in an 81–97% yield. Thus, the diversity points in this scheme are the aromatic substituents in the 1,2,3-triazolyl chalcones, which are later used in the production of a variety of spiro compounds. The structures of the synthesized compounds were assigned based on spectroscopy techniques, including IR spectral analyses, ^1^H- and ^13^C-NMR, and CHN analysis, which showed that the synthesized structures had high consistency with the proposed chemical structures. The ^1^H-NMR spectrum of azide compound (**3**) showed the assigned protons and matched with the proposed structure (Figure S1). A singlet at *δ* 12.57 ppm was assigned to the -NH proton, and a singlet at *δ* 4.64 ppm was assigned to the –CH_2_ protons. The ^13^C-NMR spectrum showed the characteristic carbon signals of the proposed compound (Figure S2). Similarly, for 1,2,3-triazolyl ketone (**4**), the corresponding ^1^H-NMR spectrum (Figure S3) exhibited a singlet in the region at *δ* 12.62 ppm for one proton of the –NH group of benzimidazole, a singlet in the region at *δ* 5.86 ppm related to the two protons of the –CH_2_ group that was apparent, and two singlets at *δ* 2.54 ppm and *δ* 2.53 ppm corresponding to the protons present in the two methyl groups –CH_3_ and –COCH_3_, respectively. The ^13^C-NMR spectrum (Figure S4) exhibited a signal at *δ* 193.88 ppm for one carbon of the C=O group and two carbon signals for –COCH_3_ and –CH_3_ groups at *δ* 27.99 and 9.37 ppm, respectively. Additionally, compound (**4**) was obtained in a crystalline form suited for single-crystal X-ray diffraction analysis. Similarly, the proposed structures of 1,2,3-triazolyl chalcones (**5a-n**) were confirmed using the same spectroscopic analysis tools. The infrared (IR) spectrum (Figure S10) data for compound **5f** supported the proposed structure of the compound. In the IR spectrum, (C=O) stretching was found in the expected region at 1666 cm^−1^. In addition, the derivative showed a typical absorption band due to (–NH) at 3430 cm^−1^. The ^1^H-NMR spectrum (Figure S11) of compound (**5f**) exhibited a singlet at *δ* 12.63 ppm for the –NH proton and two doublet peaks at *δ* 7.88 ppm and 7.75 ppm for the α,β-unsaturated protons H_β_ and H_α_, respectively, with a *J* value of 16 Hz, confirming *trans* coupling and indicating the presence of olefinic protons in the *E* form. The ^13^C-NMR spectrum (Figure S12) showed the characteristic carbon signals of the proposed compound **5f**.

### 2.2. Synthesis of Spiro Compounds (***8a-n***)

Spiro compounds (**8a-n**) were synthesized via a three-component reaction in which the 32CA reaction between 1,2,3 triazolyl chalcones (**5a-n**) and the azomethine ylide (AY), generated by the interaction between isatin and octahydroindole-2-carboxylic acid, was a key-reaction step (Scheme 1). All three-component reactions were carried out by heating an equimolar mixture of the chalcones (**5a-n**), isatin (**7**), and octahydroindole-2-carboxylic acid (**6**) in MeOH under reflux conditions for 3–6 h. After the completion of the reaction (which was checked using TLC), the solvent was evaporated, and the cyclized spiro compounds were purified via column chromatography to afford target spiro compounds in a pure form and in a good to excellent yield (60–85%). The structures of the synthesized spiro compounds were characterized using different spectroscopic techniques, such as FT-IR, ^1^H-NMR, ^13^C-NMR, and CHN analysis. For example, the FT-IR spectrum (Figure S22) of compound (**8f**) showed two strong absorption bands at 1724 and 1684 cm^−1^ corresponding to the oxindole ring carbonyl and the benzimidazole ring carbonyl, respectively. The strongest absorption band appeared at 3428 cm^−1^ due to the –NH functionality in the oxindole ring and benzimidazole ring. The ^1^H-NMR spectrum (Figure S23) of compound (**8f**) showed a singlet at *δ* 12.48 ppm due to the –NH proton of the benzimidazole ring, a singlet at *δ* 9.91 ppm due to the –NH proton of the isatin ring, and a multiplet between *δ* 7.57 and 6.35 ppm due to the presence of aromatic protons. A singlet at *δ* 5.74 ppm due to –CH_2_ protons and two singlets at *δ* 2.23 ppm and *δ* 1.98 ppm corresponding to protons of the two –CH_3_ groups were also observed. The ^13^C-NMR spectrum (Figure S24) showed the characteristic carbon signals of the proposed compound **8f**. The final cycloadduct stereochemistry was aligned with and matched a similar type of [3+2] cycloaddition reaction, which proceeded via complete *ortho*/*endo* selectivity [13]. Based on the reported X-ray single-crystal structure of the reported compound in Ref. [13] and a comparison of its ^1^H-NMR spectrum with the ^1^H-NMR data for compound **8a** as an example, we observed that the chemical shifts of the protons for the stereogenic centers totally matched.

### 2.3. Structural Features

**Figure 2:**
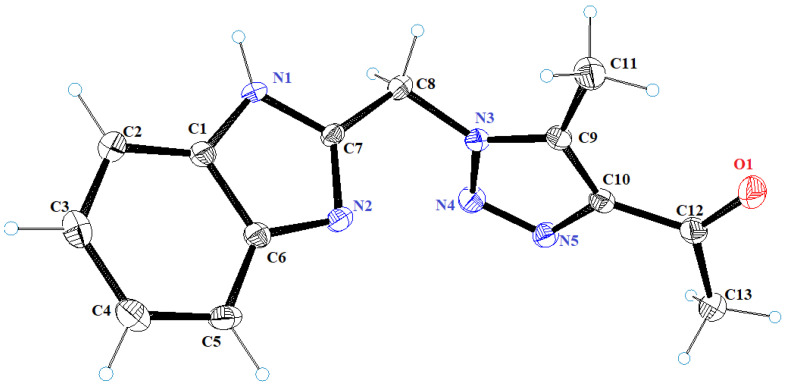
ORTEP view of the synthesized 1,2,3-triazolyl ketone (**4**).

The synthesized 1,2,3-triazolyl ketone (**4**), a precursor of chalcones **5a-n**, crystallizes in tetragonal space group *P4(3)*, having four asymmetric units inside the unit cell (see Figure 2 and Table 1). The compound is a benzimidazole derivative that contains a methyl- and acetaldehyde-substituted triazole at the C8 position having a bond length of 1.450 Å. The nine-membered benzimidazole ring C1-C7/N1/N2 and the triazole ring N3-N5/C9/C10 form a dihedral angle [18] of 81.15°. All the other bond lengths and angles observed were not unusual. The mean plane deviation in the benzimidazole ring C1-C7/N1/N2 was 0.022 Å for C1. The CCDC number for the synthesized 1,2,3-triazolyl ketone (**4**) is 2282490.

### 2.4. Supramolecular Features

**Figure 3:**
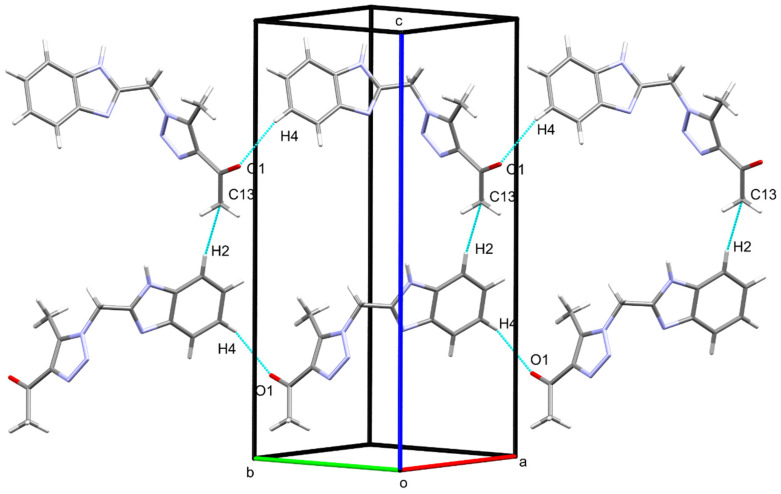
Molecular-packing diagram for 1,2,3-triazolyl ketone (**4**).

PLATON [19] analysis revealed the presence of both conventional and non-conventional hydrogen bonding [20]. Generally, this analysis showed that N(1)–H2A···N2, C8–H8AB···O1, and C4–H4···O1 inter-molecular interactions were involved in the unit-cell packing. Among them, the N1–H2A···N2 interaction, involved in connecting molecules along the *c-axis*, is the strongest one, having a bond distance of 2.06 Å. The O1 carbonyl oxygen of the acetaldehyde moiety is responsible for connecting two molecules along the *a-axis* via C8–H8AB···O1 and C4–H4···O1 interactions, with hydrogen bond distances of 2.50 and 2.58 Å, respectively (see Table 2). Hence, the unit-cell packing was determined to be two-dimensional, as chain elongation occurred in both *a-* and *c-axis* accordingly (see Figure 3).

### 2.5. MEDT Study of the 32CA Reaction between AY ***9*** and Chalcone ***5a***

In order to understand the experimental formation of spiro compounds **8a-n**, the 32CA reaction of chalcone **5a** with AY **9**, generated in situ through the reaction between (2*R*)-octahydro-1*H*-indole-2-carboxylic acid **6** and isatin **7**, was theoretically studied from the perspective of MEDT [17].

#### 2.5.1. Analysis of Reactivity Indicators

The reactivity indices defined within Conceptual DFT (CDFT) [21,22] provide valuable insights into the prediction and comprehension of reactivity in polar reactions [23]. Table 3 summarizes the global reactivity indices, including the electronic chemical potential (μ), chemical hardness (η), electrophilicity (ω), and nucleophilicity (N), for both AY **9** and chalcone **5a**.

The electronic chemical potential (μ) [24] of AY **9** is −2.92 eV, which is higher than that of chalcone **5a** (−4.46 eV). This disparity indicates that in a polar 32CA reaction, a global electron density transfer (GEDT) [25] will occur between AY **9** and chalcone **5a**. Consequently, AY **9** acts as a nucleophile, while chalcone **5a** serves as an electrophile, classifying the corresponding 32CA reaction as a forward electron density flux (FEDF) process [26].

AY **9** exhibits an electrophilicity (ω) index [27] of 0.61 eV, categorizing it as a moderate electrophile according to the electrophilicity scale [22,28]. Additionally, it possesses a nucleophilicity (*N*) index [29] of 5.02 eV, classifying it as a strong nucleophile based on the nucleophilicity scale [22,28]. In fact, its nucleophilic character exceeds 4.0 eV, earning it the title of a supernucleophile [23,28]. On the other hand, chalcone **5a** presents electrophilicity (ω) and nucleophilicity (*N*) indices of 1.25 eV and 2.96 eV, respectively. This characterizes it as a strong electrophile and positions it at the borderline between moderate and strong nucleophiles.

The combination of the supernucleophilic character of AY **9** and the strong electrophilic character of chalcone **5a** suggests that the corresponding FEDF 32CA reaction will possess a highly polar character [23]. This heightened polarity enhances reaction rates by reducing activation energies due to the generation of more favorable nucleophilic/electrophilic interactions.

#### 2.5.2. Study of the Competitive Reaction Paths

**Scheme 2:**
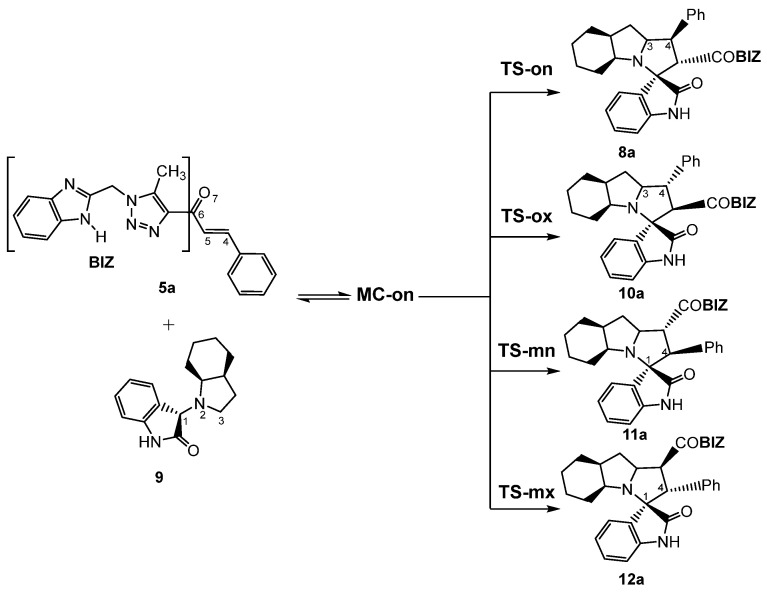
Competitive reaction paths associated with the 32CA reaction of AY **9** with chalcone **5a**.

**Figure 4:**
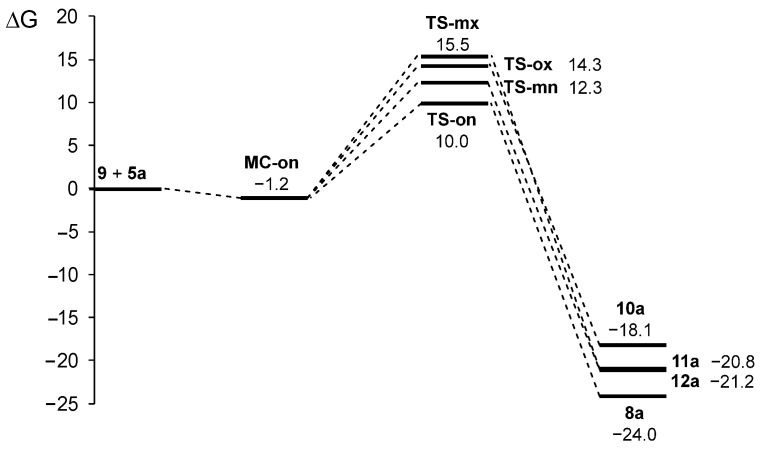
*ω*B97X-D/6-311G(d,p) Gibbs free energy profile, in kcal·mol^−1^ as well as in methanol at 60 °C, for the 32CA reaction of AY **9** with chalcone **5a**.

Due to the non-symmetry of the reagents, the 32CA reaction between AY **9** and chalcone **5a** can take place along two *ortho*/*meta* regioisomeric reaction paths, two *endo*/*exo* stereoisomeric paths, and two facial diastereoisomeric paths, thus leading to up to eight different cycloadducts. However, as the octahydroindole substituent of AY **9** hinders one of its two diastereoisomeric faces, only the less-hindered approach leading to the four isomeric reaction paths depicted in Scheme 2 was studied. For clarity, a reaction mechanism roadmap showing the main isomeric possibilities is provided in Figure S32 in the Supplementary Materials. Note that due to the presence of a methylene (–CH_2_) in chalcone **5a**, the benzimidazole (–BIZ) substituent can be oriented either towards or away from AY **9**, thus adding four possible isomeric paths. All of the eight paths were studied, but only the most favourable ones, with the –BIZ fragment situated away from the AY framework, are discussed herein. In addition, a conformational analysis of the reagents and products was performed whenever different conformers were possible in order to consider only the most stable structures. The Gibbs free energy profiles associated with the four competitive reaction paths are represented in Figure 4, while full thermodynamic data are given in Table S1 in the Supplementary Materials.

Upon analyzing the stationary points along the four reaction paths, it becomes evident that the 32CA reaction occurs through a one-step mechanism. Each reaction path exhibits a stable molecular complex (MC) formed through weak intermolecular interactions between the reagents. However, due to the thermodynamic equilibrium between the several MCs, only the most stable complex, **MC-on**, was chosen as the energy reference. The formation of **MC-on** is slightly exergonic, releasing 1.2 kcal·mol^−1^ of energy (see Figure 4). Considering the formation of **MC-on**, the activation Gibbs free energies for the selected isomeric paths range from 11.1 kcal·mol^−1^ (**TS-on**) to 16.6 kcal·mol^−1^ (**TS-mx**). On the other hand, the reaction Gibbs free energies fall between −18.1 kcal·mol^−1^ (**10a**) and −24.0 kcal·mol^−1^ (**8a**). The highly exergonic nature of this reaction suggests irreversibility under the experimental conditions, indicating that the reaction is controlled kinetically. Using the Eyring–Polanyi kinetics equation [30], the following product distribution was predicted: 97.0% (**8a**), 0.1% (**10a**), 2.8% (**11a**), and 0.0% (**12a**). This demonstrates complete *ortho*/*endo* selectivity, exclusively yielding the formation of **8a** through **TS-on**, aligning with the experimental data.

**Figure 5:**
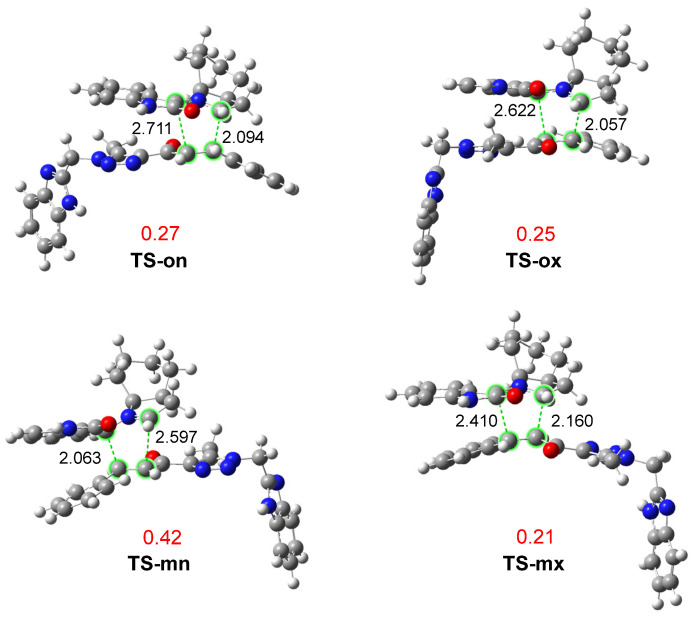
*ω*B97X-D/6-311G(d,p) optimized geometries in methanol of the TSs involved in the 32CA reaction of AY **9** with chalcone **5a**. Distances between the interacting carbon atoms, highlighted in green, are given in angstroms, Å, while GEDT values, in red, are given in average number of electrons, e.

Figure 5 presents the optimized geometries of the four isomeric transition states (TSs) in methanol. The C–C distances between the interacting carbons provide insights into the C–C single-bond formation processes. Except for the most unfavorable **TS-mx**, the other three TSs exhibit an asynchronous behavior, with the shorter C–C distance involving the most electrophilic β-conjugated C4 carbon of chalcone **5a**. The most favorable **TS-on**, characterized by C3–C4 and C1–C5 distances of 2.094 and 2.711 Å, respectively, has the highest degree of asynchronicity. Examining the intrinsic reaction coordinate (IRC) path [31] from the highly asynchronous **TS-on** to **8a** reveals that the 32CA reaction follows a non-concerted *two-stage, one-step* mechanism [32]. In this mechanism, the formation of the second C1–C5 single bond commences only after the first C3–C4 single bond is fully formed (see Figure S33 in the Supplementary Materials).

Figure 5 also provides the GEDT [25] values for the four isomeric TSs. The GEDT taking place in the TS is a measure of the polarity of the 32CA reaction. GEDT values below 0.05 e indicate non-polar processes, while values above 0.20 e indicate polar processes. Among the TSs, the most favorable **TS-on** exhibits a GEDT value of 0.27 e. This high value arises from the supernucleophilic nature of AY **9** and the strong electrophilic character of chalcone **5a** (refer to Table 3). Consequently, the 32CA reaction through **TS-on** possesses a significant polar character, which accounts for its low activation Gibbs free energy of 11.1 kcal·mol^−1^ and the overall *endo* stereoselectivity observed. Note that polar cycloaddition reactions typically exhibit *endo* stereoselectivity. Furthermore, the positive GEDT sign computed at the AY framework of the TS indicates an electron density flow from AY **9** to chalcone **5a**, classifying this 32CA reaction as FEDF, [26] in accordance with the previous analysis of the reactivity indices.

## 3. Materials and Methods

### 3.1. Synthesis of Chalcones (***5a-n***) and Spiro Compounds (***8a-n***)

#### 3.1.1. Synthesis of 2-(Chloromethyl)-1H-benzo[d]imidazole **2**

In accordance with the Phillip’s reaction, a mixture of *o*-phenylenediamine (10 mmol, 1.08 g) and chloroacetic acid (10 mmol, 0.945 g) was stirred under reflux conditions in the presence of 4N HCl (40 mL) for approximately 4 h. Then, the reaction mixture was cooled at room temperature, and the pH was adjusted to 9 by adding NH_4_OH solution. The obtained precipitate was collected via filtration, washed with water, dried, and recrystallized from ethanol. The pure product was a pale-yellow-colored solid whose melting point was approximately 150–152 °C, and the yield was 92%.

#### 3.1.2. Synthesis of 2-(Azidomethyl)-1H-benzo[d]imidazole **3**

NaN_3_ (11 mmol, 0.715 g) was added to a solution of 2-(chloromethyl)-1*H*-benzo[*d*]imidazole **2** (10 mmol, 1.66 g) in DMSO (10 mL), and the mixture was stirred for 3–4 h. After completion of reaction (as indicated via TLC), water (50 mL) was added with consistent stirring for 10 min. Then, the organic phase was separated using ethyl acetate. The extract was dried over anhydrous sodium sulphate. Evaporation of the solvent gave the crude product which was purified via column chromatography using hexane: ethylacetate (80:20), as an eluent, which was recrystallized from absolute ethanol.


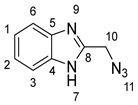


Yield: 75%; m.p.: 119–121 °C; a pale-yellow-colored solid compound; ^1^H-NMR (500 MHz, DMSO-*d*_6_) *δ* 12.57 (s, 1H, NH), 7.52–7.50 (m, 2H, ArH), 7.16–7.13 (m, 2H, ArH), and 4.64 (s, 2H, CH_2_); ^13^C-NMR (126 MHz, DMSO-*d*_6_) *δ* 149.8 (C-8), 134.4 (C-5), 122.5 (C-4), 118.6 (C-6), 113.9 (C-3), 109.4 (C-2, C-1), and 47.8 (C-10); Anal. for C_8_H_7_N_5_; Calcd: C, 55.48; H, 4.07; N, 40.44 Found: C, 55.52; H, 4.03; N, 40.38; [M+] *m*/*z*: 173.

#### 3.1.3. Synthesis of 1-(1-((1H-Benzo[d]imidazol-2-yl)methyl)-5-methyl-1H-1,2,3-triazol-4-yl)ethan-1-one **4**

2-(Azidomethyl)-1*H*-benzo[*d*]imidazole **3** (2 mmol, 0.346 g) was added to a solution of (2 mmol, 0.2 g) of acetylacetone and (2 mmol, 0.276 g) of K_2_CO_3_ in 10 mL of DMSO. The mixture was stirred for 3 h at 25 °C and poured into ice water, and the precipitate was filtered off and recrystallized from ethylacetate/ethanol. The yield was 0.4 g (78%) of white crystalline compound **4**.


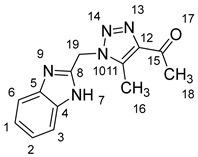


Yield: 78%; m.p.: 192–194 °C; a white, solid compound; ^1^H-NMR (500 MHz, DMSO-*d*_6_) *δ* 12.62 (s, 1H, NH), 7.49 (m, 2H, ArH), 7.14 (m, 2H, ArH), 5.86 (s, 2H, CH_2_), 2.54 (s, 3H, CH_3_), and 2.53 (s, 3H, COCH_3_). ^13^C-NMR (126 MHz, DMSO-*d*_6_) *δ* 193.9 (C-15), 148.5 (C-11), 143.4 (C-8), 138.6 (C-5), 134.0 (C-4), 123.2 (C-12), 122.1 (C-2, C-1), 119.4 (C-6), 112.1 (C-3), 45.9 (C-19), 28.0 (C-18), and 9.4 (C-16); Anal. for C_13_H_13_N_5_O; Calcd: C, 61.17; H, 5.13; N, 27.43 Found: C, 61.12; H, 5.08; N, 27.39; [M+] *m*/*z*: 255.

#### 3.1.4. General Procedure for Synthesis of Chalcones **5a-n**

A mixture of 1.1 mmol of aromatic aldehydes was added to a solution of acetyl derivative **4** (1 mmol, 0.255 g) in EtOH (20 mL). Then, a 10% solution of KOH was added dropwise at 20 °C with stirring. The reaction mixture was stirred for 10 h. After the completion of the reaction (monitored via TLC), the mixture was poured over crushed ice. The separated precipitate was filtered, washed with water, and dried. The residue was purified via column chromatography (30% ethyl acetate/*n*-hexane) to afford purely derived chalcones **5a-n**.

1-(1-((1*H*-Benzo[*d*]imidazol-2-yl)methyl)-5-methyl-1*H*-1,2,3-triazol-4-yl)-3-phenylprop-2-en-1-one **5a**.


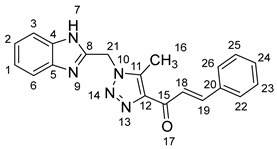


Yield: 83%; m.p.: 158–160 °C; a yellow, solid compound; ^1^H-NMR (500 MHz, DMSO-*d*_6_) *δ* 12.64 (s, 1H, NH), 7.94 (d, *J* = 16.0 Hz, 1H, CH_β_), 7.83–7.73 (m, 4H, ArH, CH_α_), 7.42 (m, 4H, ArH), 7.18–7.12 (m, 2H, ArH), 5.91 (s, 2H, CH_2_), 2.63 (s, 3H, CH_3_); ^13^C-NMR (126 MHz, DMSO-*d*_6_) *δ* 183.9 (C-15), 148.5 (C-11), 143.6 (C-19), 143.5 (C-8), 143.4 (C-5), 139.9 (C-4), 135.1 (C-20), 134.9 (C-12), 131.3 and 130.0 (C-26, (C-22), 129.8 (C-24), 129.7 and 129.6 ((C-23, C-25), 129.2 (C-2), 123.3 (C-1), 122.2 (C-18), 119.4 (C-6), 112.1 (C-3), 46.0 (C-21), and 9.6 (C-16); Anal. for C_20_H_17_N_5_O; Calcd: C, 69.96; H, 4.99; N, 20.40 Found: C, 69.92; H, 5.03; N, 20.35; [M+] *m*/*z*: 343; IR (KBr, cm^−1^): 1572 (C=N), 1663 (C=O), 3431 (NH).

1-(1-((1*H*-Benzo[*d*]imidazol-2-yl)methyl)-5-methyl-1*H*-1,2,3-triazol-4-yl)-3-(4-methoxyphenyl)prop-2-en-1-one **5b**.


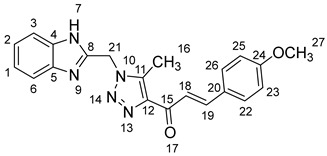


Yield: 94%; m.p.: 215–217 °C; a yellow, solid compound; ^1^H-NMR (500 MHz, DMSO-*d*_6_) *δ* 12.69 (s, 1H, NH), 7.83 (d, *J* = 10.3 Hz, 1H, CH_β_), 7.77 (m, 2H, ArH, CH_α_), 7.59 (d, *J* = 8.1 Hz, 1H, ArH), 7.50 (d, *J* = 7.3 Hz, 1H, ArH), 7.27–7.11 (m, 3H, ArH), 7.02 (d, *J* = 8.8 Hz, 2H, ArH), 5.95 (s, 2H, CH_2_), 3.82 (s, 3H, OCH_3_), and 2.67 (s, 3H, CH_3_); ^13^C-NMR (126 MHz, DMSO-*d*_6_) *δ* 183.9 (C-15), 162.0 (C-24), 148.6 (C-11), 143.8 (C-19), 143.5 (C-8), 143.4 (C-5), 139.7 (C-4), 134.9 (C-12), 131.2 and 127.6 (C-26, C-22), 123.2 (C-20), 122.2 (C-2), 120.9 (C-1), 119.5 (C-18), 115.2 (C-6, C-3), 112.2 (C-25, C-23), 56.0 (C-27), 46.0 (C-21), and 9.6 (C-16); Anal. for C_21_H_19_N_5_O_2_; Calcd: C, 67.55; H, 5.13; N, 18.76 Found: C, 67.64; H, 5.10; N, 18.69; [M+] *m*/*z*: 373; IR (KBr, cm^−1^): 1568 (C=N), 1665 (C=O), 3432 (NH).

1-(1-((1*H*-Benzo[*d*]imidazol-2-yl)methyl)-5-methyl-1*H*-1,2,3-triazol-4-yl)-3-(2,4-dichlorophenyl)prop-2-en-1-one **5c**.


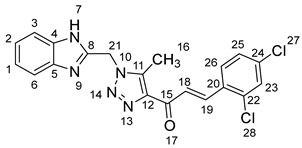


Yield: 88%; m.p.: 182–185 °C; a yellow, solid compound; ^1^H-NMR (500 MHz, DMSO-*d*_6_) *δ* 12.60 (s, 1H, NH), 8.03 (d, *J* = 12.1 Hz, 1H, CH_β_), 7.97 (d, *J* = 12.9 Hz, 1H, CH_α_), 7.83 (d, *J* = 8.4 Hz, 1H, Ar-H), 7.58 (d, *J* = 8.4 Hz, 2H, Ar-H), 7.06–7.01 (m, 3H, Ar-H), 6.86 (d, *J* = 8.4 Hz, 1H, Ar-H), 5.92 (s, 2H, CH_2_), and 2.63 (s, 3H, CH_3_); ^13^C-NMR (126 MHz, DMSO-*d*_6_) *δ* 189.4 (C-15), 149.6 (C-11), 148.4 (C-19), 144.6 (C-8), 143.4 (C-5), 143.2 (C-4), 140.4 (C-22), 138.9 (C-12), 137.1 (C-20), 131.7 (C-28), 130.8 (C-23), 130.2 (C-25), 129.6 (C-24), 128.8 (C-2), 128.7 (C-1), 125.6 (C-18), 122.2 (C-6, C-3), 45.9 (C-21), and 9.6 (C-16); Anal. for C_20_H_15_C_l2_N_5_O; calcd: C, 58.27; H, 3.67; N, 16.99 Found: C, 58.22; H, 3.63; N, 16.94; [M+] *m*/*z*: 411; IR (KBr, cm^−1^): 1570 (C=N), 1664 (C=O), 3430 (NH).

1-(1-((1*H*-Benzo[*d*]imidazol-2-yl)methyl)-5-methyl-1*H*-1,2,3-triazol-4-yl)-3-(4-chlorophenyl)prop-2-en-1-one **5d**.


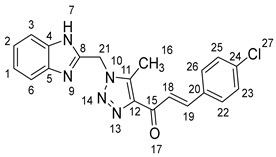


Yield: 84%; m.p.: 200–202 °C; a pale-yellow, solid compound; ^1^H-NMR (500 MHz, DMSO-*d*_6_) *δ* 12.63 (s, 1H, NH), 7.93 (d, *J* = 16.1 Hz, 1H, CH_β_), 7.81 (d, *J* = 8.6 Hz, 2H, ArH), 7.77 (d, *J* = 16.1 Hz, 1H, COCH_α_), 7.53 (d, *J* = 7.9 Hz, 1H, ArH), 7.48 (d, *J* = 8.6 Hz, 2H, ArH), 7.45 (d, *J* = 7.9 Hz, 1H, ArH), 7.17 (t, *J* = 7.5 Hz, 1H, ArH), 7.12 (t, *J* = 7.5 Hz, 1H, ArH), 5.91 (s, 2H, CH_2_), and 2.62 (s, 3H, CH_3_); ^13^C-NMR (126 MHz, DMSO-*d*_6_) *δ* 183.8 (C-15), 148.5 (C-11), 143.6 (C-19), 143.4 (C-8), 142.1 (C-5), 140.0 (C-4), 135.8 (C-24), 134.9 (C-20), 133.9 (C-12), 131.0 (C-22, C-28), 129.7 (C-25, C-23), 124.0 (C-2), 123.2 (C-1), 122.1 (C-18), 119.4 (C-6), 112.1 (C-3), 46.0 (C-21), and 9.6 (C-16); Anal. for C_20_H_16_ClN_5_O; Calcd: C, 63.58; H, 4.27; N, 18.54 Found: C, 63.61; H, 4.21; N, 18.59; [M+] *m*/*z*: 377; IR (KBr, cm^−1^): 1567 (C=N), 1665 (C=O), 3433 (NH).

1-(1-((1*H*-Benzo[*d*]imidazol-2-yl)methyl)-5-methyl-1*H*-1,2,3-triazol-4-yl)-3-(4-fluorophenyl)prop-2-en-1-one **5e**.


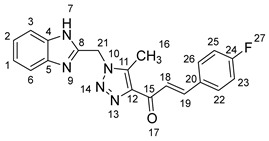


Yield: 98%; m.p.: 220–222 °C; a pale-yellow, solid compound; ^1^H-NMR (500 MHz, DMSO-*d*_6_) *δ* 12.63 (s, 1H, NH), 7.88 (d, *J* = 16.0 Hz, 1H, CH_β_), 7.85 (dd, *J* = 6.0, 2.8 Hz, 2H, ArH), 7.78 (d, *J* = 16.0 Hz, 1H, COCH_α_), 7.54–7.44 (m, 2H, ArH), 7.26 (t, *J* = 8.8 Hz, 2H, ArH), 7.17–7.11 (m, 2H, ArH), 5.91 (s, 2H, CH_2_), and 2.62 (s, 3H, CH_3_); ^13^C-NMR (126 MHz, DMSO-*d*_6_) *δ* 183.8 (C-15), 165.0 and 163.0 (C-24), 148.5 (C-11), 143.6 (C-19), 142.3 (C-8), 139.9 (C-5), 131.7 (C-4), 131.6 (C-12), 123.2 (C-20), 122.2 (C-26, C-22), 119.4 (C-2, C-1), 116.7 (C-18), 116.6 (C-6, C-3), 112.1 (C-25, C-23), 46.0 (C-21), and 9.6 (C-16); Anal. for C_20_H_16_FN_5_O; Calcd: C, 66.47; H, 4.46; N, 19.38 Found: C, 66.41; H, 4.49; N, 19.43; [M+] *m*/*z*: 361; IR (KBr, cm^−1^): 1568 (C=N), 1661 (C=O), 3431 (NH).

1-(1-((1*H*-benzo[*d*]imidazol-2-yl)methyl)-5-methyl-1*H*-1,2,3-triazol-4-yl)-3-(p-tolyl)prop-2-en-1-one **5f**.


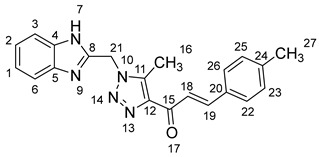


Yield: 84%; m.p.: 207–209 °C; a yellow, solid compound; ^1^H-NMR (500 MHz, DMSO-*d*_6_) *δ* 12.63 (s, 1H, NH), 7.88 (d, *J* = 16.0 Hz, 1H, CH_β_), 7.75 (d, *J* = 16.0 Hz, 1H, COCH_α_), 766 (d, *J* = 8.1 Hz, 2H, ArH), 7.54 (d, *J* = 7.6 Hz, 1H, ArH), 7.45 (d, *J* = 7.6 Hz, 1H, ArH), 7.24 (d, *J* = 8.1 Hz, 2H, ArH), 7.16 (t, *J* = 7.5 Hz, 1H, ArH), 7.14 (t, *J* = 7.5 Hz, 1H, ArH), 5.91 (s, 2H, CH_2_), 2.62 (s, 3H, CH_3_), and 2.31 (s, 3H, Ph-CH_3_); ^13^C-NMR (126 MHz, DMSO-*d_6_*) *δ* 183.9 (C-15), 148.5 (C-11), 143.7 (C-19), 143.6 (C-8), 143.4 (C-5), 141.4 (C-4), 139.8 (C-24), 134.9 (C-20), 132.2 (C-25), 130.3 (C-23), 129.3 (C-26), 123.2 (C-22), 122.3 (C-2), 122.1 (C-1), 119.4 (C-18), 112.1 (C-6, C-3), 46.0 (C-21), 21.6 (C-27), and 9.6 (C-16); Anal. for C_21_H_19_N_5_O; Calcd: C, 70.57; H, 5.36; N, 19.59 Found: C, 70.62; H, 5.40; N, 19.54; [M+] *m*/*z*: 357; IR (KBr, cm^−1^): 1569 (C=N), 1666 (C=O), 3430 (NH).

1-(1-((1*H*-Benzo[*d*]imidazol-2-yl)methyl)-5-methyl-1*H*-1,2,3-triazol-4-yl)-3-(4-bromophenyl)prop-2-en-1-one **5g**.


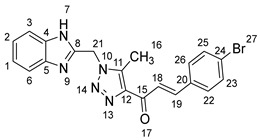


Yield: 82%; m.p.: 194–196 °C; a yellow, solid compound; ^1^H-NMR (500 MHz, DMSO-*d*_6_) *δ* 12.61 (s, 1H, NH), 7.89 (d, *J* = 16.1 Hz, 1H, CH_β_), 7.83 (d, *J* = 8.5 Hz, 2H, ArH), 7.76 (d, *J* = 16.1 Hz, 1H, COCH_α_), 7.55 (d, *J* = 8.0 Hz, 1H, ArH), 7.46 (d, *J* = 8.5 Hz, 2H, ArH), 7.45 (d, *J* = 8.0 Hz, 1H, ArH), 7.16 (t, *J* = 7.5 Hz, 1H, ArH), 7.13 (t, *J* = 7.5 Hz, 1H, ArH), 5.92 (s, 2H, CH_2_), and 2.61 (s, 3H, CH_3_); ^13^C-NMR (126 MHz, DMSO-*d_6_*) *δ* 184.8 (C-15), 149.5 (C-11), 142.6 (C-19), 142.2 (C-8), 140.1 (C-5), 134.8 (C-4), 133.9 (C-20), 132.9 (C-25), 131.0 (C-23), 129.6 (C-12), 124.0 (C-26), 123.2 (C-22), 122.1 (C-2, C-1), 119.4 (C-18), 118.6 (C-24), 112.1 (C-6, C-3), 46.1 (C-21), and 9.6 (C-16); Anal. for C_20_H_16_BrN_5_O; Calcd: C, 56.89; H, 3.82; N, 16.58 Found: C, 56.85; H, 3.86; N, 16.55; [M+] *m*/*z*: 421; IR (KBr, cm^−1^): 1573 (C=N), 1664 (C=O), 3433 (NH).

1-(1-((1*H*-Benzo[*d*]imidazol-2-yl)methyl)-5-methyl-1*H*-1,2,3-triazol-4-yl)-3-(*m*-tolyl)prop-2-en-1-one **5h**.


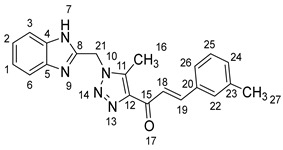


Yield: 93%; m.p.: 220–222 °C; a pale-yellow, solid compound; ^1^H-NMR (500 MHz, DMSO-*d*_6_) *δ* 12.63 (s, 1H, NH), 7.92 (d, *J* = 16.0 Hz, 1H, CH_β_), 7.75 (d, *J* = 16.0 Hz, 1H, COCH_α_), 7.60 (s, 1H, ArH), 7.54 (dd, *J* = 7.9, 3.7 Hz, 2H ArH), 7.46 (d, *J* = 7.5 Hz, 1H, ArH), 7.31 (t, *J* = 7.6 Hz, 1H ArH), 7.24 (d, *J* = 8.7 Hz, 1H, ArH), 7.17 (t, *J* = 7.6 Hz, 1H, ArH), 7.12 (t, J = 7.6 Hz, 1H, ArH), 5.91 (s, 2H, CH_2_), 2.62 (s, 3H, CH_3_), and 2.32 (s, 3H, CH_3_); ^13^C-NMR (126 MHz, DMSO-*d_6_*) *δ* 183.9 (C-15), 148.5 (C-11), 143.6 (C-19), 143.4 (C-8), 139.9 (C-5), 138.9 (C-4), 134.9 (C-23), 132.1 (C-20), 129.5 (C-12), 129.4 (C-25), 126.7 (C-24), 123.2 (C-22), 123.1 (C-26), 122.1 (C-2, C-1), 119.4 (C-18), 112.1 (C-6, C-3), 46.0 (C-21), 21.4 (C-27), and 9.6 (C-16); Anal. for C_21_H_19_N_5_O; Calcd: C, 70.57; H, 5.36; N, 19.59 Found: C, 70.62; H, 5.31; N, 19.54; [M+] *m*/*z*: 357; IR (KBr, cm^−1^): 1567 (C=N), 1666 (C=O), 3431 (NH).

1-(1-((1*H*-Benzo[*d*]imidazol-2-yl)methyl)-5-methyl-1*H*-1,2,3-triazol-4-yl)-3-(thiophen-2-yl)prop-2-en-1-one **5i**.


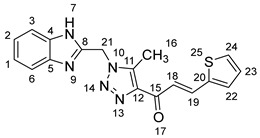


Yield: 95%; m.p.: 212–214 °C; a pale-yellow, solid compound; ^1^H-NMR (500 MHz, DMSO-*d*_6_) *δ* 12.62 (s, 1H, NH), 7.95 (d, *J* = 15.8 Hz, 1H, CH_β_), 7.75 (d, *J* = 5.1 Hz, 1H, thiophene), 7.63 (d, *J* = 15.8 Hz, 1H, COCH_α_), 7.62 (d, *J* = 5.1 Hz, 1H, thiophene), 7.53 (d, *J* = 7.8 Hz, 1H, ArH), 7.45 (d, *J* = 7.8 Hz, 1H, ArH), 7.18–7.15 (m, 2H, ArH, thiophene), 7.11 (t, *J* = 7.5 Hz, 1H, ArH), 5.90 (s, 2H, CH_2_), 2.61 (s, 3H, CH_3_); ^13^C-NMR (126 MHz, DMSO-*d_6_*) *δ* 183.3 (C-15), 148.5 (C-11), 143.5 (C-8), 143.4 (C-20), 140.2 (C-5), 139.8 (C-4), 136.3 (C-19), 134.9 (C-12), 134.0 (C-24), 130.9 (C-22), 129.4 (C-23), 123.2 (C-18), 122.1 (C-2), 121.6 (C-1), 119.4 (C-6), 112.1 (C-3), 46.0 (C-21), and 9.6 (C-16); Anal. for C_18_H_15_N_5_OS; Calcd: C, 61.87; H, 4.33; N, 20.04 Found: C, 61.82; H, 4.36; N, 20.07; [M+] *m*/*z*: 349; IR (KBr, cm^−1^): 1567 (C=N), 1665 (C=O), 3428 (NH).

1-(1-((1*H*-Benzo[*d*]imidazol-2-yl)methyl)-5-methyl-1*H*-1,2,3-triazol-4-yl)-3-(3,4,5-trimethoxyphenyl)prop-2-en-1-one **5j**.


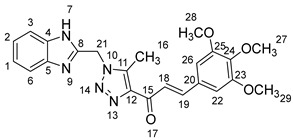


Yield: 89%; m.p.: 203–205 °C; a yellow, solid compound; ^1^H-NMR (500 MHz, DMSO-*d*_6_) *δ* 12.65 (s, 1H, NH), 7.90 (d, *J* = 16.0 Hz, 1H, CH_β_), 7.79 (d, *J* = 16.0 Hz, 1H, COCH_α_), 7.61 (d, *J* = 8.0 Hz, 2H, ArH), 7.24 (d, *J* = 8.0 Hz, 2H, ArH), 7.12 (s, 2H, ArH), 5.93 (s, 2H, CH_2_), 3.69 (s, 6H, OCH_3_), 3.48 (s, 3H, OCH_3_), and 2.64 (s, 3H, CH_3_); ^13^C-NMR (126 MHz, DMSO-*d_6_*) *δ* 185.91 (C-15), 158.33 (C-25, C-23), 148.41 (C-11), 143.65 (C-19), 143.50 (C-8), 143.27 (C-5), 141.42 (C-4), 139.78 (C-24), 134.82 (C-12), 132.21 (C-20), 131.26 (C-2), 129.25 (C-1), 123.14 (C-18), 122.45 (C-6), 121.76 (C-3), 122.11 (C-26), 119.42 (C-22), 60.45 (C-27), 52.12 (C-29, C-28), 45.99 (C-21), and 9.57 (C-16); Anal. for C_23_H_23_N_5_O_4_; Calcd: C, 63.73; H, 5.35; N, 16.16 Found: C, 63.75; H, 5.31; N, 16.14; [M+] *m*/*z*: 433; IR (KBr, cm^−1^): 1568 (C=N), 1668 (C=O), 3432 (NH).

1-(1-((1*H*-Benzo[*d*]imidazol-2-yl)methyl)-5-methyl-1*H*-1,2,3-triazol-4-yl)-3-(3-nitrophenyl)prop-2-en-1-one **5k**.


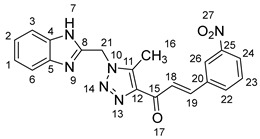


Yield: 86%; m.p.: 188–190 °C; a brown, solid compound; ^1^H-NMR (500 MHz, DMSO-*d*_6_) *δ* 12.63 (s, 1H, NH), 8.13–8.02 (m, 2H, ArH), 7.84 (d, *J* = 16.0 Hz, 1H, CH_β_), 7.75 (d, *J* = 16.0 Hz, 1H, COCH_α_), 7.61 (d, *J* = 8.1 Hz, 2H, ArH), 7.59 (d, *J* = 7.5 Hz, 1H, ArH), 7.55 (d, *J* = 7.5 Hz, 1H, ArH), 7.24 (d, *J* = 8.1 Hz, 2H, ArH), 5.95 (s, 2H, CH_2_), 2.63 (s, 3H, CH_3_); ^13^C-NMR (126 MHz, DMSO-*d_6_*) *δ* 185.9 (C-15), 148.5 (C-11), 146.8 (C-25), 143.7 (C-19), 143.4 (C-8), 141.5 (C-5), 139.9 (C-4), 134.9 (C-20), 132.2 (C-22), 130.3 (C-12), 129.3 (C-23), 123.3 (C-24), 122.4 (C-2, C-1), 122.1 (C-26), 119.5 (C-18), 112.2 (C-6, C-3), 46.0 (C-21), and 9.6 (C-16); Anal. for C_20_H_16_N_6_O_3_; Calcd: C, 61.85; H, 4.15; N, 21.64 Found: C, 61.78; H, 4.10; N, 21.68; [M+] *m*/*z*: 388; IR (KBr, cm^−1^): 1569 (C=N), 1667 (C=O), 3429 (NH).

1-(1-((1*H*-Benzo[*d*]imidazol-2-yl)methyl)-5-methyl-1*H*-1,2,3-triazol-4-yl)-3-(4-(dimethylamino)phenyl)prop-2-en-1-one **5l**.


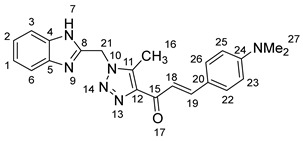


Yield: 83%; m.p.: 197–198 °C; a red, solid compound; ^1^H-NMR (500 MHz, DMSO-*d*_6_) *δ* 12.61 (s, 1H, NH), 7.68 (d, *J* = 6.6 Hz, 2H), 7.61–7.55 (m, 2H), 7.49 (d, *J* = 3.3 Hz, 2H), 7.14 (dd, *J* = 6.1, 3.1 Hz, 2H), 6.71 (d, *J* = 9.1 Hz, 2H), 5.88 (s, 2H, CH_2_), 2.96 (s, 6H, NCH_3_), and 2.60 (s, 3H, CH_3_); ^13^C-NMR (126 MHz, DMSO-*d*_6_) *δ* 183.6 (C-15), 152.6 (C-24), 148.6 (C-11), 144.5 (C-19), 144.0 (C-8), 139.2 (C-5, C-4), 131.1 (C-12), 122.2 (C-26, C-22), 117.5 (C-20), 112.4 (C-2, C-1), 111.6 (C-25, C-23), 45.9 (C-27), and 9.5 (C-16); Anal. for C_22_H_22_N_6_O; Calcd: C, 68.38; H, 5.74; N, 21.75 Found: C, 68.34; H, 5.69; N, 21.80; [M+] *m*/*z*: 386; IR (KBr, cm^−1^): 1575(C=N), 1669 (C=O), 3434 (NH).

1-(1-((1*H*-Benzo[*d*]imidazol-2-yl)methyl)-5-methyl-1*H*-1,2,3-triazol-4-yl)-3-(3-bromophenyl)prop-2-en-1-one **5m**.


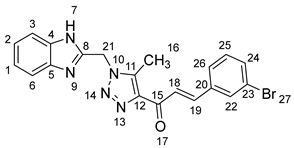


Yield: 95%; m.p.: 185–187 °C; a pale-yellow, solid compound; ^1^H-NMR (500 MHz, DMSO-*d*_6_) *δ* 12.59 (s, 1H, NH), 7.87 (d, *J* = 16.1 Hz, 1H, CH_β_), 7.81 (d, *J* = 8.5 Hz, 2H, ArH), 7.74 (d, *J* = 16.1 Hz, 1H, COCH_α_), 7.52 (d, *J* = 8.0 Hz, 1H, ArH), 7.41 (d, *J* = 8.5 Hz, 2H, ArH), 7.45 (d, *J* = 8.0 Hz, 1H, ArH), 7.18 (t, *J* = 7.5 Hz, 1H, ArH), 7.15 (t, *J* = 7.5 Hz, 1H, ArH), 5.89 (s, 2H, CH_2_), and 2.63 (s, 3H, CH_3_); ^13^C-NMR (126 MHz, DMSO-*d_6_*) *δ* 185.2 (C-15), 149.5 (C-11), 142.6 (C-19), 143.4 (C-8), 142.2 (C-5, C-4), 140.1 (C-12), 134.8 (C-20), 133.9 (C-22), 132.9 (C-24), 131.0 (C-25), 129.6 (C-26), 124.0 (C-2), 123.2 (C-1), 122.1 (C-23), 119.4 (C-18), 112.1 (C-6, C-3), 46.1 (C-21), and 9.6 (C-16); Anal. for C_20_H_16_BrN_5_O; Calcd: C, 56.89; H, 3.82; N, 16.58 Found: C, 56.89; H, 3.78; N, 16.52; [M+] *m*/*z*: 421; IR (KBr, cm^−1^): 1568 (C=N), 1666 (C=O), 3431 (NH).

1-(1-((1*H*-Benzo[*d*]imidazol-2-yl)methyl)-5-methyl-1*H*-1,2,3-triazol-4-yl)-3-mesitylprop-2-en-1-one **5n**.


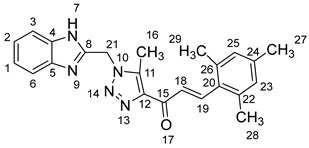


Yield: 92%; m.p.: 195–197 °C; a yellow, solid compound; ^1^H-NMR (400 MHz, DMSO-*d*_6_) *δ* 12.82 (s, 1H, NH), 7.95 (d, *J* = 16.1 Hz, 1H, CH_β_), 7.62–7.50 (m, 3H, CH_α_, ArH), 7.20 (m, 2H, ArH), 6.97 (s, 2H, ArH), 5.96 (s, 2H, CH_2_), 2.67 (s, 3H, CH_3_), 2.36 (s, 6H, CH_3_), and 2.25 (s, 3H, CH_3_); ^13^C NMR (101 MHz, DMSO-*d*_6_) *δ* 183.9 (C-15), 148.5 (C-11), 143.7 (C-19), 141.3 (C-8), 140.1 (C-5), 138.9 (C-4), 137.6 (C-24), 131.2 (C-26, C-22), 129.9 (C-20), 129.9 (C-12), 127.9 (C-25, C-23), 122.8 (C-2, C-1), 45.9 (C-21), 21.6 (C-27), 21.5 (C-29, C-29), and 9.6 (C-16); Anal. for C_23_H_23_N_5_; Calcd: C,71.67; H,6.01; N,18.17 Found: C,71.63; H,5.97; N, 18.20; [M+] *m*/*z*: 385; IR (KBr, cm^−1^): 1567 (C=N), 1665 (C=O), 3430 (NH).

#### 3.1.5. General Procedure for [3+2] Cycloaddition Reactions for the Synthesis of Spiro Compounds **8a-n**

A mixture of the chalcone derivatives **5a-n** (0.5 mmol), isatin (0.5 mmol, 73.5 mg), and octahydroindole-2-carboxylic acid (0.5 mmol, 84.5 mg) in methanol (15 mL) was refluxed using an oil bath for an appropriate time of 3–4 h. After completion of the reaction (Monitored using TLC), the solvent volume was removed under vacuum. The crude was purified via column chromatography on silica gel (30% ethyl acetate in *n*-hexane), yielding the spiro compounds as solids in a pure form.

(*1’S,2’R,3S*)-2’-(1-((1*H*-Benzo[*d*]imidazol-2-yl)methyl)-5-methyl-1*H*-1,2,3-triazole-4-carbonyl)-1’-phenyl-1’,2’,4a’,5’,6’,7’,8’,8a’,9’,9a’-decahydrospiro[indoline-3,3’-pyrrolo [1,2-*a*]indol]-2-one **8a**.


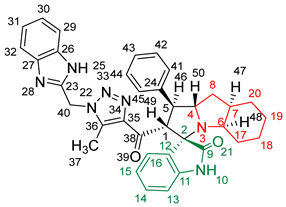


Yield: 81%; m.p.: 195–197 °C; a pale-yellow, solid compound; ^1^H-NMR (500 MHz, DMSO-*d*_6_) *δ* 12.43 (s, 1H, NH), 9.87 (s, 1H, NH), 7.52 (d, *J* = 8.0 Hz, 1H, ArH), 7.42 (d, *J* = 8.0 Hz, 1H, ArH), 7.33 (t, *J* = 7.4 Hz, 2H, ArH), 7.26 (t, *J* = 7.4 Hz, 2H, ArH), 7.19–7.09 (m, 4H, ArH), 6.89 (t, *J* = 7.6 Hz, 1H, ArH), 6.81 (t, *J* = 7.6 Hz, 1H, ArH), 6.29 (d, *J* = 7.5 Hz, 1H, ArH), 5.69 (s, 2H, CH_2_), 5.05 (d, *J* = 12.3 Hz, 1H, COCH), 4.05–3.98 (m, 1H), 3.81 (t, *J* = 11.1 Hz, 1H), 3.18 (d, *J* = 7.2 Hz, 1H, aliphatic-H), 2.08–2.01 (m, 1H, aliphatic-H), 1.92 (s, 3H, CH_3_), 1.83 (dt, *J* = 12.4, 6.4 Hz, 1H, aliphatic-H), 1.53 (dd, *J* = 12.1, 6.5 Hz, 1H, aliphatic-H), 1.47–1.37 (m, 2H, aliphatic-H), 1.33–1.24 (m, 2H, aliphatic-H), 1.07–0.95 (m, 2H, aliphatic-H), 0.76 (d, *J* = 8.8 Hz, 1H, aliphatic-H), and 0.66 (d, *J* = 11.1 Hz, 1H, aliphatic-H); ^13^C-NMR (126 MHz, DMSO-*d*_6_) *δ* 191.6 (C-38), 180.3 (C-9), 148.1 (C-12), 143.2 (C-36), 142.5 (C-23), 140.3 (C-11), 138.5 (C-24), 134.8 (C-27), 129.3 (C-26), 129.1 (C-15), 128.3 (C-35), 128.0 (C-44, C-42), 127.2 (C-45, C-41), 124.3 (C-14), 123.1 (C-43), 122.1 (C-31, C-30), 120.7 (C-16), 119.4 (C-32, 29), 112.1 (C-13), 109.5 (C-2), 71.3 (C-6), 65.9 (C-4), 57.1 (C-1), 52.9 (C-40), 45.7 (C-7), 41.7 (C-20), 37.2 (C-17), 28.3 (C-5), 28.0 (C-8), 25.0 (C-19), 19.8 (C-18), and 8.5 (37); Anal. for C_36_H_35_N_7_O_2_; Calcd: C, 72.34; H, 5.90; N, 16.40 Found: C, 72.32; H, 5.88; N, 16.44; [M+] *m*/*z*: 597; IR (KBr, cm^−1^): 1617 (C=N), 1681–1724 (C=O), 3427(NH).

(*1’S,2’R,3S*)-2’-(1-((1*H*-Benzo[*d*]imidazol-2-yl)methyl)-5-methyl-1*H*-1,2,3-triazole-4-carbonyl)-1’-(4-methoxyphenyl)-1’,2’,4a’,5’,6’,7’,8’,8a’,9’,9a’-decahydrospiro[indoline-3,3’-pyrrolo[1,2-*a*]indol]-2-one **8b**.


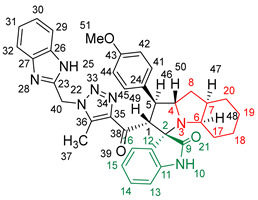


Yield: 89%; m.p.: 178–180 °C; a pale-yellow, solid compound; ^1^H-NMR (400 MHz, DMSO-*d_6_*) *δ* 12.50 (s, 1H, NH), 9.92 (s, 1H, NH), 7.57 (d, *J* = 8.1 Hz, 1H, ArH), 7.47 (d, *J* = 8.1 Hz, 1H, ArH), 7.29 (d, *J* = 8.8 Hz, 2H, ArH), 7.18 (m, 3H, ArH), 6.94 (t, *J* = 7.7 Hz, 1H, ArH), 6.86 (dd, *J* = 7.7, 5.5 Hz, 3H, ArH), 6.35 (d, *J* = 8.1 Hz, 1H, ArH), 5.74 (s, 2H, CH_2_), 5.04 (d, *J* = 12.5 Hz, 1H, COCH), 4.04 (q, *J* = 8.1 Hz, 1H), 3.79 (t, *J* = 11.4 Hz, 1H), 3.69 (s, 3H, OCH_3_), 3.21 (d, *J* = 4.4 Hz, 1H, aliphatic-H), 2.08–2.03 (m, 1H, aliphatic-H), 1.98 (s, 3H, CH_3_), 1.88–1.79 (m, 1H, aliphatic-H), 1.56 (dd, *J* = 12.1, 6.2 Hz, 1H, aliphatic-H), 1.52–1.41 (m, 2H, aliphatic-H), 1.34 (d, *J* = 13.2 Hz, 2H, aliphatic-H), 0.98 (dt, *J* = 24.2, 11.0 Hz, 2H, aliphatic-H), 0.80 (t, *J* = 12.8 Hz, 1H, aliphatic-H), and 0.70 (d, *J* = 11.7 Hz, 1H, aliphatic-H); ^13^C-NMR (126 MHz, DMSO-*d_6_*) *δ* 191.7 (C-38), 180.3 (C-9), 158.5 (C-43), 148.1 (C-12), 143.2 (C-36), 142.5 (C-23), 138.5 (C-11), 135.3 (C-27), 132.0 (C-26), 129.3 (C-15), 129.0 (C-35), 128.3 (C-24), 124.4 (C-45, C-41), 123.2 (C-14), 122.1 (C-31, C-30), 120.6 (C-16), 119.4 (C-32, C-29), 114.5 (C-13), 112.1 (C-44, C-42), 109.5 (C-2), 71.3 (C-6), 71.2 (C-4), 66.0 (C-51), 65.5 (C-1), 57.1 (C-40), 55.5 (C-7), 52.2 (C-20), 45.7 (C-17), 41.7 (C-5), 37.2 (C-8), 28.3 (C-19), 28.0 (C-18), and 8.5 (C-37); Anal. for C_37_H_37_N_7_O_3_; Calcd: C, 70.79; H, 5.94; N, 15.62 Found: C, 70.82; H, 5.90; N, 15.64; [M+] *m*/*z*: 627; IR (KBr, cm^−1^): 1618 (C=N), 1682–1722 (C=O), 3429 (NH).

(*1’S,2’R,3S*)-2’-(1-((1*H*-Benzo[*d*]imidazol-2-yl)methyl)-5-methyl-1*H*-1,2,3-triazole-4-carbonyl)-1’-(2,4-dichlorophenyl)-1’,2’,4a’,5’,6’,7’,8’,8a’,9’,9a’-decahydrospiro[indoline-3,3’-pyrrolo[1,2-*a*]indol]-2-one **8c**.


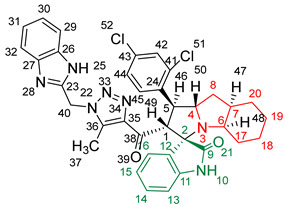


Yield: 71%; m.p.: 202–204 °C; a yellow, solid compound; ^1^H-NMR (500 MHz, DMSO-*d*_6_) *δ* 12.44 (s, 1H, NH), 9.94 (s, 1H, NH), 7.53 (s, 1H, ArH), 7.37 (dd, *J* = 8.5, 2.3 Hz, 2H, ArH), 7.14–6.82 (m, 7H, ArH), 6.30 (d, *J* = 7.6 Hz, 1H, ArH), 5.68 (s, 2H, CH_2_), 5.13 (d, *J* = 12.2 Hz, 1H, COCH), 4.40–4.32 (m, 1H), 3.93 (t, *J* = 6.6 Hz, 1H), 3.15 (d, J = 4.3 Hz, 1H, aliphatic-H), 2.24–2.14 (m, 2H, aliphatic-H), 1.93 (s, 3H, CH_3_), and 1.83–0.71 (m, 7H, aliphatic-H); ^13^C-NMR (126 MHz, DMSO-*d_6_*) *δ* 191.2 (C-38), 180.0 (C-9), 148.1 (C-12), 142.9 (C-36), 142.7 (C-23), 139.6 (C-11), 138.7 (C-27), 136.2 (C-26), 135.2 (C-15), 132.3 (C-43), 129.8 (C-41), 129.5 (C-24), 128.4 (C-35), 127.7 (C-45), 124.6 (C-42), 124.1 (C-44), 121.8 (C-14), 121.4 (C-31, 30), 120.9 (C-16), 114.7 (C-32, C-29), 112.4 (C-13), 109.7 (C-2), 71.7 (C-6), 71.1 (C-4), 66.2 (C-1), 57.2 (C-40), 52.7 (C-7), 48.1 (C-20), 45.7 (C-8), 41.6 (C-17), 36.8, 35.0, 30.3, 24.8 (C-19), 22.6 (C-18), 19.8 (C-5), and 8.6 (C-37); Anal. for C_36_H_33_Cl_2_N_7_O_2_; Calcd: C, 64.87; H, 4.99; N, 14.71 Found: C, 64.83; H, 5.02; N, 14.73; [M+] *m*/*z*: 665; IR (KBr, cm^−1^): 1615 (C=N), 1683–1724 (C=O), 3428 (NH).

(1’*S*,2’*R*,3*S*)-2’-(1-((1*H*-Benzo[d]imidazol-2-yl)methyl)-5-methyl-1*H*-1,2,3-triazole-4-carbonyl)-1’-(4-chlorophenyl)-1’,2’,4a’,5’,6’,7’,8’,8a’,9’,9a’-decahydrospiro[indoline-3,3’-pyrrolo[1,2-*a*]indol]-2-one **8d**.


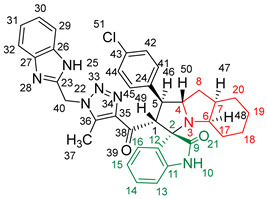


Yield: 85%; m.p.: 179–181 °C; a yellow, solid compound; ^1^H-NMR (400 MHz, DMSO-*d*_6_) *δ* 12.48 (s, 1H, NH), 9.93 (s, 1H, NH), 7.57 (d, *J* = 8.1 Hz, 1H, ArH), 7.47 (d, *J* = 8.1 Hz, 1H, ArH), 7.41 (d, *J* = 8.1 Hz, 2H, ArH), 7.37 (d, *J* = 8.1 Hz, 2H, ArH), 7.22 (d, *J* = 7.3 Hz, 1H, ArH), 7.17 (m, 2H, ArH), 6.93 (t, *J* = 7.7 Hz, 1H, ArH), 6.84 (t, *J* = 7.7 Hz, 1H, ArH), 6.34 (d, *J* = 8.1 Hz, 1H, ArH), 5.74 (s, 2H, CH_2_), 5.03 (d, *J* = 12.5 Hz, 1H,COCH), 4.05 (q, *J* = 8.8, 8.1 Hz, 1H), 3.86 (t, *J* = 11.0 Hz, 1H), 3.21 (d, *J* = 3.7 Hz, 1H, aliphatic-H), 2.08 (dd, *J* = 11.0, 5.9 Hz, 1H, aliphatic-H), 1.97 (s, 3H, CH_3_), 1.86 (q, *J* = 6.2 Hz, 1H, aliphatic-H), 1.56 (dd, *J* = 11.7, 6.6 Hz, 1H, aliphatic-H), 1.47 (t, *J* = 16.5 Hz, 2H, aliphatic-H), 1.37–1.28 (m, 2H, aliphatic-H), 1.07–0.93 (m, 2H, aliphatic-H), 0.80 (t, *J* = 12.8 Hz, 1H, aliphatic-H), and 0.70 (d, *J* = 13.9 Hz, 1H, aliphatic-H); ^13^C-NMR (101 MHz, DMSO-*d_6_*) *δ* 191.5 (C-38), 180.3 (C-9), 148.1 (C-12), 143.3 (C-36), 143.2 (C-23), 142.6 (C-11), 139.4 (C-27), 138.6 (C-26), 134.9 (C-24), 131.8 (C-15), 130.0 (C-43), 129.1 (C-35), 128.3 (C-45, C-41), 127.1 (C-44, C-42), 124.3 (C-14), 123.2 (C-31, C-30), 122.2 (C-16), 120.7 (C-32), 119.2 (C-29), 112.2 (C-13), 109.6 (C-2), 71.9 (C-6), 66.1 (C-4), 58.1 (C-1), 50.9 (C-40), 45.6 (C-7), 41.3 (C-20), 36.6 (C-17), 28.3 (C-5), 27.6 (C-8), 24.6 (C-19), 18.8 (C-18), and 8.5 (C-37); Anal. for C_36_H_34_ClN_7_O_2_; Calcd: C, 68.40; H, 5.42; N, 15.51 Found: C, 68.45; H, 5.44; N, 15.48; [M+] *m*/*z*: 631; IR (KBr, cm^−1^): 1617 (C=N), 1684–1723 (C=O), 3426 (NH).

(*1’S,2’R,3S*)-2’-(1-((1*H*-Benzo[*d*]imidazol-2-yl)methyl)-5-methyl-1*H*-1,2,3-triazole-4-carbonyl)-1’-(4-fluorophenyl)-1’,2’,4a’,5’,6’,7’,8’,8a’,9’,9a’-decahydrospiro[indoline-3,3’-pyrrolo[1,2-*a*]indol]-2-one **8e**.


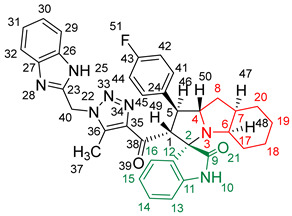


Yield: 96%; m.p.: 183–185 °C; a white, solid compound; ^1^H-NMR (400 MHz, DMSO-*d*_6_) *δ* 12.50 (s, 1H, NH), 9.94 (s, 1H, NH), 7.57 (d, *J* = 8.1 Hz, 1H, ArH), 7.47 (d, *J* = 7.3 Hz, 1H, ArH), 7.44–7.39 (m, 2H, ArH), 7.22 (d, *J* = 7.3 Hz, 1H, ArH), 7.18 (d, *J* = 7.3 Hz, 1H, ArH), 7.14 (t, *J* = 8.8 Hz, 3H, ArH), 6.93 (t, *J* = 7.3 Hz, 1H, ArH), 6.85 (t, *J* = 7.3 Hz, 1H, ArH), 6.34 (d, *J* = 7.3 Hz, 1H, ArH), 5.74 (s, 2H, CH_2_), 5.03 (d, *J* = 11.7 Hz, 1H, COCH), 4.06 (q, *J* = 8.8, 8.1 Hz, 1H), 3.86 (t, *J* = 11.4 Hz, 1H), 3.21 (d, *J* = 3.7 Hz, 1H, aliphatic-H), 2.07 (dt, *J* = 11.0, 5.5 Hz, 1H, aliphatic-H), 1.97 (s, 3H, CH_3_), 1.90–1.81 (m, 1H, aliphatic-H), 1.56 (dd, *J* = 11.7, 5.9 Hz, 1H, aliphatic-H), 1.52–1.41 (m, 2H, aliphatic-H), 1.33 (d, *J* = 12.5 Hz, 2H, aliphatic-H), 1.06–0.91 (m, 2H, aliphatic-H), 0.80 (t, *J* = 13.2 Hz, 1H, aliphatic-H), and 0.70 (d, *J* = 11.7 Hz, 1H, aliphatic-H); ^13^C-NMR (101 MHz, DMSO-*d*_6_) *δ* 191.6 (C-38), 180.3 (C-9), 162.8 and 160.4 (C-43), 148.2 (C-12), 143.3 (C-36), 143.2 (C-23), 142.6 (C-11), 138.6 (C-27), 136.4 (C-26), 134.9 (C-24), 130.5 (C-15), 129.8 (C-35), 129.2 (C-45, C-41), 124.3 (C-14), 122.4 (C-31, C-30), 120.2 (C-16), 118.7 (C-44, C-42), 115.5 (C-32, C-29), 113.4 (C-13), 109.4 (C-2) 71.3 (C-6), 64.2 (C-4), 60.1 (C-1), 52.9 (C-40), 45.8 (C-7), 41.9 (C-20), 37.4 (C-17), 28.9 (C-5), 28.8 (C-8), 24.2 (C-19), 19.2 (C-18), and 9.9 (C-37); Anal. for C_36_H_34_FN_7_O_2_; Calcd: C, 70.23; H, 5.57; N, 15.92 Found: C, 70.19; H, 5.60; N, 15.56; [M+] *m*/*z*: 615; IR (KBr, cm^−1^): 1618 (C=N), 1686–1725 (C=O), 3429 (NH).

(*1’S,2’R,3S*)-2’-(1-((1*H*-Benzo[*d*]imidazol-2-yl)methyl)-5-methyl-1*H*-1,2,3-triazole-4-carbonyl)-1’-(p-tolyl)-1’,2’,4a’,5’,6’,7’,8’,8a’,9’,9a’-decahydrospiro[indoline-3,3’-pyrrolo[1,2-*a*]indol]-2-one **8f**.


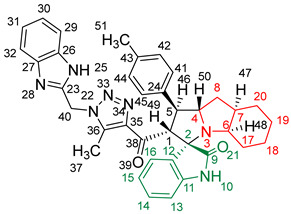


Yield: 88%; m.p.: 182–184 °C; a yellow, solid compound; ^1^H-NMR (400 MHz, DMSO-*d*_6_) *δ* 12.48 (s, 1H, NH), 9.91 (s, 1H, NH), 7.57 (d, *J* = 8.1 Hz, 1H, ArH), 7.47 (d, *J* = 8.1 Hz, 1H, ArH), 7.26 (d, *J* = 8.1 Hz, 2H, ArH), 7.20 (t, *J* = 6.6 Hz, 2H, ArH), 7.15 (d, *J* = 8.8 Hz, 1H, ArH), 7.10 (d, *J* = 8.1 Hz, 2H, ArH), 6.93 (t, *J* = 7.7 Hz, 1H, ArH), 6.85 (t, *J* = 7.7 Hz, 1H, ArH), 6.35 (d, *J* = 8.1 Hz, 1H, ArH), 5.74 (s, 2H, CH_2_), 5.08 (d, *J* = 11.7 Hz, 1H,COCH), 4.03 (m, 1H), 3.80 (t, *J* = 11.0 Hz, 1H), 3.22 (d, *J* = 4.4 Hz, 1H, aliphatic-H), 2.23 (s, 3H, Ph-CH_3_), 2.08 (d, *J* = 5.9 Hz, 1H, aliphatic-H), 1.98 (s, 3H, CH_3_), 1.84 (dt, J = 13.9, 6.2 Hz, 1H, aliphatic-H), 1.56 (dd, *J* = 12.1, 6.2 Hz, 1H, aliphatic-H), 1.46 (m, 2H, aliphatic-H), 1.34 (m, 2H, aliphatic-H), 1.07–0.93 (m, 2H, aliphatic-H), 0.79 (d, *J* = 12.5 Hz, 1H, aliphatic-H), and 0.70 (d, *J* = 13.9 Hz, 1H, aliphatic-H); ^13^C-NMR (101 MHz, DMSO-*d_6_*) *δ* 191.7 (C-38), 180.4 (C-9), 148.2 (C-12), 143.3 (C-36), 142.6 (C-23), 138.5 (C-11), 137.2 (C-27), 136.3 (C-26), 135.0 (C-24), 129.7 (C-43), 129.3 (C-15), 128.3 (C-35), 128.0 (C-44, C-42), 127.9 (C-45, C-41), 124.4 (C-14), 123.6 (C-31, C-30), 120.7 (C-16), 119.6 (C-32, C-29), 112.4 (C-13), 109.6 (C-2), 71.4 (C-6), 65.9 (C-4), 57.2 (C-1), 52.7 (C-40), 45.8 (C-7), 41.8 (C-20), 37.3 (C-17), 28.0 (C-5), 25.0 (C-8), 21.1 (C-19), 19.8 (C-18), 14.7 (C-51), and 8.6 (C-37); Anal. for C_37_H_37_N_7_O_2_; Calcd: C, 72.65; H, 6.10; N, 16.03 Found: C, 72.68; H, 6.06; N, 15.97; [M+] *m*/*z*: 611; IR (KBr, cm^−1^): 1617 (C=N), 1684–1724 (C=O), 3428 (NH).

(*1’S,2’R,3S*)-2’-(1-((1*H*-Benzo[*d*]imidazol-2-yl)methyl)-5-methyl-1*H*-1,2,3-triazole-4-carbonyl)-1’-(4-bromophenyl)-1’,2’,4a’,5’,6’,7’,8’,8a’,9’,9a’-decahydrospiro[indoline-3,3’-pyrrolo[1,2-*a*]indol]-2-one **8g**.


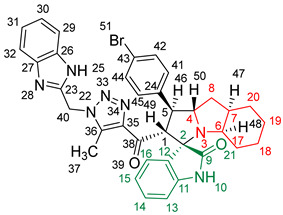


Yield: 82%; m.p.: 186–188 °C; a yellow, solid compound; ^1^H-NMR (400 MHz, DMSO-*d*_6_) *δ* 12.46 (s, 1H, NH), 9.91 (s, 1H, NH), 7.57 (d, *J* = 7.3 Hz, 1H, ArH), 7.50 (d, *J* = 8.8 Hz, 2H, ArH), 7.47 (d, *J* = 8.1 Hz, 1H, ArH), 7.35 (d, *J* = 8.1 Hz, 2H, ArH), 7.22–7.12 (m, 3H, ArH), 6.93 (t, *J* = 7.7 Hz, 1H, ArH), 6.84 (t, *J* = 7.7 Hz, 1H, ArH), 6.33 (d, *J* = 8.1 Hz, 1H, ArH), 5.73 (s, 2H, CH_2_), 5.02 (d, *J* = 11.7 Hz, 1H, COCH), 4.04 (q, *J* = 9.2 Hz, 1H), 3.85 (t, *J* = 11.0 Hz, 1H), 3.21 (d, *J* = 4.4 Hz, 1H, aliphatic-H), 2.08 (dt, *J* = 11.0, 5.5 Hz, 1H, aliphatic-H), 1.97 (s, 3H, CH_3_), 1.90–1.83 (m, 1H, aliphatic-H), 1.56 (dd, *J* = 11.7, 5.9 Hz, 1H, aliphatic-H), 1.45 (dt, *J* = 15.4, 3.3 Hz, 2H, aliphatic-H), 1.32 (dd, *J* = 14.7, 7.3 Hz, 2H, aliphatic-H), 1.07–0.95 (m, 2H, aliphatic-H), 0.83–0.75 (m, 1H, aliphatic-H), 0.69 (d, *J* = 13.9 Hz, 1H, aliphatic-H); ^13^C-NMR (101 MHz, DMSO-*d*_6_) *δ* 191.5 (C-38), 180.2 (C-9), 148.1 (C-12), 143.3 (C-36), 143.1 (C-23), 142.6 (C-11), 139.8 (C-27), 138.6 (C-26), 134.9 (C-24), 132.0 (C-15), 131.6 (C-35), 131.3 (C-44, C-42),130.3 (C-45, C-41), 124.3 (C-14), 122.7 (C-43), 121.8 (C-31, C-30), 120.7 (C-16), 120.3 (C-32, C-29), 112.1 (C-13), 109.6 (C-2), 71.3 (C-6), 65.3 (C-4), 57.2 (C-1), 52.4 (C-40), 45.8 (C-7), 41.7 (C-20), 37.3 (C-17), 28.3 (C-5), 28.0 (C-8), 25.0 (C-19), 19.8 (C-18), and 8.5 (C-37); Anal. for C_36_H_34_BrN_7_O_2_; Calcd: C, 63.91; H, 5.07; N, 14.49 Found: C, 63.94; H, 5.10; N, 14.45; [M+] *m*/*z*: 675; IR (KBr, cm^−1^): 1616 (C=N), 1684–1724(C=O), 3427 (NH).

(*1’S,2’R,3S*)-2’-(1-((1*H*-Benzo[*d*]imidazol-2-yl)methyl)-5-methyl-1H-1,2,3-triazole-4-carbonyl)-1’-(*m*-tolyl)-1’,2’,4a’,5’,6’,7’,8’,8a’,9’,9a’-decahydrospiro[indoline-3,3’-pyrrolo[1,2-*a*]indol]-2-one **8h**.


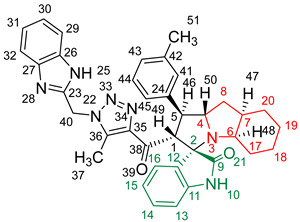


Yield: 71%; m.p.: 175–177 °C; a pale-yellow, solid compound; ^1^H-NMR (500 MHz, DMSO-*d*_6_) *δ* 12.44 (s, 1H, NH), 9.86 (s, 1H, NH), 7.51 (d, *J* = 8.1 Hz, 1H, ArH), 7.43 (d, *J* = 8.1 Hz, 1H, ArH), 7.19–7.10 (m, 6H, ArH), 6.95 (dd, *J* = 6.7, 1.9 Hz, 1H, ArH), 6.88 (t, *J* = 7.5 Hz, 1H, ArH), 6.80 (t, *J* = 7.5 Hz, 1H, ArH), 6.28 (d, *J* = 7.8 Hz, 1H, ArH), 5.68 (s, 2H, CH_2_), 5.02 (d, *J* = 12.2 Hz, 1H, COCH), 4.02 (q, *J* = 5.1, 4.6 Hz, 1H), 3.78–3.71 (m, 1H), 3.17 (d, *J* = 4.4 Hz, 1H, aliphatic-H), 2.23 (s, 3H, Ph-CH_3_), 2.06–2.00 (m, 1H, aliphatic-H), 1.91 (s, 3H, CH_3_), 1.83–1.77 (m, 1H, aliphatic-H), 1.52 (dd, *J* = 11.9, 6.2 Hz, 1H, aliphatic-H), 1.47–1.38 (m, 2H, aliphatic-H), 1.32–1.25 (m, 2H, aliphatic-H), 1.02–0.97 (m, 1H, aliphatic-H), 0.96–0.87 (m, 1H, aliphatic-H), 0.76 (t, *J* = 13.1 Hz, 1H, aliphatic-H), 0.66 (d, *J* = 11.4 Hz, 1H, aliphatic-H); ^13^C-NMR (126 MHz, DMSO-*d_6_*) *δ* 191.6 (C-38), 180.3 (C-9), 148.1 (C-12), 143.2 (C-36), 142.5 (C-23), 140.3 (C-11), 138.5 (C-24), 138.1 (C-27), 130.1 (C-26), 129.3 (C-42), 129.0 (C-15), 128.6 (C-35), 128.2 (C-41), 127.9 (C-44), 125.3 (C-43), 124.3 (C-14), 122.9 (C-45), 121.9 (C-31,C-30), 120.6 (C-16), 119.3 (C-32, C-29), 113.2 (C-13), 109.5 (C-2), 71.3 (C-6), 66.0 (C-4), 57.1 (C-1), 52.9 (C-40), 45.7 (C-7), 41.7 (C-20), 37.3 (C-17), 28.3 (C-5), 28.0 (C-8), 25.0 (C-19), 21.6 (C-51), 19.8 (C-18), and 8.5 (C-37); Anal. for C_37_H_37_N_7_O_2_; Calcd: C, 72.65; H, 6.10; N, 16.03 Found: C, 72.62; H, 6.13; N, 15.98; [M+] *m*/*z*: 611; IR (KBr, cm^−1^): 1617 (C=N), 1682–1721 (C=O), 3432 (NH).

(*1’R,2’R,3S*)-2’-(1-((1*H*-Benzo[*d*]imidazol-2-yl)methyl)-5-methyl-1*H*-1,2,3-triazole-4-carbonyl)-1’-(thiophen-2-yl)-1’,2’,4a’,5’,6’,7’,8’,8a’,9’,9a’-decahydrospiro[indoline-3,3’-pyrrolo[1,2-*a*]indol]-2-one **8i**.


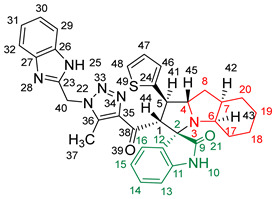


Yield: 89%; m.p.: 193–195 °C; a yellow, solid compound; ^1^H-NMR (400 MHz, DMSO-*d*_6_) *δ* 12.52 (s, 1H, NH), 9.94 (s, 1H, NH), 7.58 (d, *J* = 8.8 Hz, 1H, ArH), 7.48 (d, *J* = 8.8 Hz, 1H,ArH), 7.34 (d, *J* = 5.1 Hz, 1H, thiophene-H), 7.19 (m, 3H, ArH), 6.98–6.91 (m, 3H, ArH, thiophene-H), 6.84 (t, *J* = 7.7 Hz, 1H, thiophene-H), 6.34 (d, *J* = 8.1 Hz, 1H, ArH), 5.76 (s, 2H, CH_2_), 4.93 (d, *J* = 11.7 Hz, 1H, COCH), 4.19–4.09 (m, 2H), 3.22 (d, *J* = 4.4 Hz, 1H, aliphatic-H), 2.09 (q, *J* = 5.1 Hz, 1H, aliphatic-H), 2.01 (s, 3H, CH_3_), 1.88 (q, *J* = 6.2 Hz, 1H, aliphatic-H), 1.70 (dd, *J* = 11.7, 5.9 Hz, 1H, aliphatic-H), 1.53–1.44 (m, 2H, aliphatic-H), 1.34 (t, *J* = 10.3 Hz, 2H, aliphatic-H), 1.08–0.94 (m, 2H, aliphatic-H), 0.80 (t, *J* = 13.2 Hz, 1H, aliphatic-H), and 0.68 (d, *J* = 13.9 Hz, 1H, aliphatic-H); ^13^C-NMR (101 MHz, DMSO-*d*_6_) *δ* 191.4 (C-38), 180.1 (C-9), 148.1 (C-12), 143.4 (C-36), 143.2 (C-24), 142.5 (C-23), 138.7 (C-11), 134.5 (C-27), 129.4 (C-26), 128.4 (C-15), 127.6 (C-35), 125.1 (C-47), 124.6 (C-14), 124.1 (C-46), 123.1 (C-48), 122.6 (C-31, C-30), 122.2 (C-16), 120.7 (C-32), 119.5 (C-29), 112.3 (C-13), 109.6 (C-2), 71.4 (C-6), 71.0, 67.2 (C-6), 57.2 (C-1), 48.1 (C-40), 45.8 (C-7), 41.7 (C-20), 37.3 (C-17), 28.2 (C-5), 28.1 (C-8), 25.0 (C-19), 19.8 (C-18), and 8.6 (C-37); Anal. for C_34_H_33_N_7_O_2_S; Calcd: C, 67.64; H, 5.51; N, 16.24 Found: C, 67.61; H, 5.48; N, 16.21; [M+] *m*/*z*: 603; IR (KBr, cm^−1^): 1682–1724 (C=N), 1665 (C=O), 3426 (NH).

(*1’S,2’R,3S*)-2’-(1-((1*H*-Benzo[*d*]imidazol-2-yl)methyl)-5-methyl-1*H*-1,2,3-triazole-4-carbonyl)-1’-(3,4,5-trimethoxyphenyl)-1’,2’,4a’,5’,6’,7’,8’,8a’,9’,9a’-decahydrospiro[indoline-3,3’-pyrrolo[1,2-*a*]indol]-2-one **8j**.


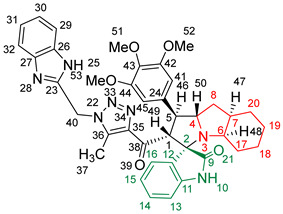


Yield: 68%; m.p.: 193–195 °C; a yellow, solid compound; ^1^H-NMR (500 MHz, DMSO-*d*_6_) *δ* 12.46 (s, 1H, NH), 9.88 (s, 1H, NH), 7.52 (d, *J* = 7.8 Hz, 1H, ArH), 7.42 (d, *J* = 7.8 Hz, 1H, ArH), 7.24 (dd, *J* = 7.5, 1.3 Hz, 1H, ArH), 7.17–7.13 (m, 1H, ArH), 7.10 (td, *J* = 7.6, 1.3 Hz, 1H, ArH), 6.93 (td, *J* = 7.6, 1.3 Hz, 1H, ArH), 6.83 (td, *J* = 7.5, 1.3 Hz, 1H, ArH), 6.62 (s, 2H, ArH), 6.35 (dd, *J* = 7.7, 1.3 Hz, 1H, ArH), 5.70 (s, 2H, CH_2_), 5.08 (d, *J* = 12.4 Hz, 1H, COCH), 4.03–3.98 (m, 1H), 3.77 (dd, *J* = 12.4, 9.9 Hz, 1H), 3.71 (s, 6H, OCH_3_), 3.56 (s, 3H, OCH_3_), 3.16 (d, *J* = 4.4 Hz, 1H, aliphatic-H), 2.08–2.03 (m, 1H, aliphatic-H), 1.96 (s, 3H, CH_3_), 1.87–1.83 (m, 1H, aliphatic-H), 1.57 (dd, *J* = 11.7, 7.2 Hz, 1H, aliphatic-H), 1.48–1.39 (m, 2H, aliphatic-H), 1.30 (dd, *J* = 13.0, 10.1 Hz, 2H, aliphatic-H), 1.01 (dd, *J* = 12.0, 3.7 Hz, 1H, aliphatic-H), 0.97–0.89 (m, 1H, aliphatic-H), 0.81–0.75 (m, 1H, aliphatic-H), and 0.68 (d, *J* = 11.7 Hz, 1H, aliphatic-H); ^13^C-NMR (126 MHz, DMSO-*d*_6_) *δ* 191.7 (C-38), 180.2 (C-9), 153.4 (C-44, C-42), 148.2 (C-12), 143.3 (C-36), 143.3 (C-11), 142.4 (C-23), 138.6 (C-24), 136.7 (C-27), 135.8 (C-26), 134.8 (C-43), 129.3 (C-15), 128.5 (C-35), 124.4 (C-14), 123.2 (C-31, C-30), 122.1, 120.7 (C-16), 119.4 (C-32, C-29), 112.1 (C-13), 109.6 (C-2), 105.0 (C-45, C-41), 71.5 (C-6), 71.0, 65.0 (C-4), 60.4 (C-51), 57.1 (C-1), 56.3 (C-53, C-52), 53.4 (C-40), 45.8 (C-7), 41.7 (C-20), 37.1 (C-17), 28.2 (C-5), 27.9 (C-8), 24.9 (C-19), 21.6, 19.8 (C-18), and 8.6 (C-37); Anal. for C_39_H_41_N_7_O_5_; Calcd: C, 68.11; H, 6.01; N, 14.26 Found: C, 68.14; H, 5.96; N, 14.23; [M+] *m*/*z*: 687; IR (KBr, cm^−1^): 1615 (C=N), 1684–1724 (C=O), 3429 (NH).

(*1’S,2’R,3S*)-2’-(1-((1*H*-Benzo[*d*]imidazol-2-yl)methyl)-5-methyl-1*H*-1,2,3-triazole-4-carbonyl)-1’-(3-nitrophenyl)-1’,2’,4a’,5’,6’,7’,8’,8a’,9’,9a’-decahydrospiro[indoline-3,3’-pyrrolo[1,2-*a*]indol]-2-one **8k**.


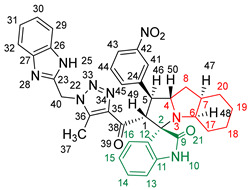


Yield: 73%; m.p.: >250 °C; a brown, solid compound; ^1^H-NMR (500 MHz, DMSO-*d*_6_) *δ* 12.47 (s, 1H, NH), 9.88 (s, 1H, NH), 7.53 (d, *J* = 8.0 Hz, 1H, ArH), 7.45 (d, *J* = 8.0 Hz, 1H, ArH), 7.21–7.12 (m, 6H, ArH), 6.93 (d, *J* = 7.5 Hz, 1H, ArH), 6.85 (t, *J* = 7.5 Hz, 1H, ArH), 6.82 (t, *J* = 7.5 Hz, 1H, ArH), 6.38 (d, *J* = 8.0 Hz, 1H, ArH), 5.70 (s, 2H, CH_2_), 5.05 (d, *J* = 12.2 Hz, 1H, COCH), 4.03 (m, 1H, CH), 3.76–3.72 (m, 1H, CH), 3.16 (d, *J* = 5.4 Hz, 1H, aliphatic-H), 2.06–2.01 (m, 1H, aliphatic-H), 1.93 (s, 3H, CH_3_), 1.83–1.76 (m, 1H, aliphatic-H), 1.52 (dd, *J* = 11.9, 6.2 Hz, 1H, aliphatic-H), 1.48–1.38 (m, 2H, aliphatic-H), 1.34–1.26 (m, 2H, aliphatic-H), 1.02–0.98 (m, 1H, aliphatic-H), 0.96–0.87 (m, 1H, aliphatic-H), 0.75 (t, *J* = 13.1 Hz, 1H, aliphatic-H), and 0.66 (d, *J* = 11.2 Hz, 1H, aliphatic-H); ^13^C-NMR (126 MHz, DMSO-*d_6_*) *δ* 191.4 (C-38), 180.5 (C-9), 148.1 (C-12), 143.1 (C-36), 142.5 (C-23), 140.2 (C-11), 138.4(C-24), 138.2 (C-27), 130.2 (C-26), 129.3 (C-42), 129.0 (C-15), 128.6 (C-35), 128.2 (C-41), 127.9 (C-44), 125.3 (C-43), 124.4 (C-14), 122.9 (C-45), 121.9 (C-31,C-30), 120.7 (C-16), 119.3 (C-32, C-29), 113.2 (C-13), 109.5 (C-2), 71.3 (C-6), 66.0 (C-4), 57.1 (C-1), 52.9 (C-40), 45.7 (C-7), 41.7 (C-20), 37.3 (C-17), 28.3 (C-5), 28.0 (C-8), 25.0 (C-19), 19.8 (C-18), and 8.5 (C-37); Anal. for C_36_H_34_N_8_O_4_; Calcd: C, 67.28; H, 5.33; N, 17.43 Found: C, 67.25; H, 5.37; N, 17.41; [M+] *m*/*z*: 642; IR (KBr, cm^−1^): 1619 (C=N), 1685–1727 (C=O), 3428 (NH).

(*1’S,2’R,3S*)-2’-(1-((1*H*-Benzo[*d*]imidazol-2-yl)methyl)-5-methyl-1*H*-1,2,3-triazole-4-carbonyl)-1’-(4-(dimethylamino)phenyl)-1’,2’,4a’,5’,6’,7’,8’,8a’,9’,9a’-decahydrospiro[indoline-3,3’-pyrrolo[1,2-*a*]indol]-2-one **8l**.


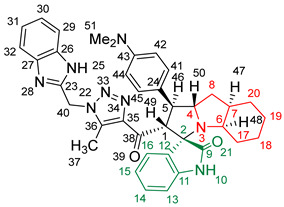


Yield: 84%; m.p.: 185–187 °C; a yellow, solid compound; ^1^H-NMR (500 MHz, DMSO-*d*_6_) *δ* 12.44 (s, 1H, NH), 9.84 (s, 1H, NH), 7.52 (d, *J* = 8.1 Hz, 1H, ArH), 7.42 (d, *J* = 7.1 Hz, 1H, ArH), 7.17–7.09 (m, 5H, ArH), 6.89 (td, *J* = 7.6, 1.3 Hz, 1H, ArH), 6.81 (td, *J* = 7.6, 1.3 Hz, 1H, ArH), 6.62 (d, *J* = 8.8 Hz, 2H, ArH), 6.30 (dd, *J* = 7.8, 1.1 Hz, 1H, ArH), 5.69 (s, 2H, CH_2_), 4.99 (d, *J* = 12.3 Hz, 1H, COCH), 4.01–3.95 (m, 1H), 3.68 (t, *J* = 10.3 Hz, 1H), 3.16 (d, *J* = 4.4 Hz, 1H, aliphatic-H), 2.78 (s, 6H, NCH_3_), 2.06–2.00 (m, 1H, aliphatic-H), 1.94 (s, 3H, CH_3_), 1.80–1.73 (m, 1H, aliphatic-H), 1.52 (dd, *J* = 11.1, 6.8 Hz, 1H, aliphatic-H), 1.46 (dd, *J* = 8.2, 3.9 Hz, 1H, aliphatic-H), 1.40 (dd, *J* = 8.9, 4.8 Hz, 1H, aliphatic-H), 1.34–1.26 (m, 2H, aliphatic-H), 1.02–0.91 (m, 2H, aliphatic-H), 0.78–0.72 (m, 1H, aliphatic-H), 0.66 (d, *J* = 10.2 Hz, 1H, aliphatic-H); ^13^C-NMR (126 MHz, DMSO-*d*_6_) *δ* 191.8 (C-38), 180.4 (C-9), 149.9 (C-43), 148.1 (C-12), 143.3 (C-36), 142.5 (C-23), 138.4 (C-11), 134.8 (C-27), 129.2 (C-26), 128.5 (C-15), 128.3 (C-35), 127.5 (C-45, C-41), 124.5 (C-24), 123.1 (C-14), 122.1 (C-31, C-30), 120.6 (C-16), 119.5 (C-32, C-29), 113.3 (C-44, C-42), 112.1(C-13), 109.5 (C-2), 71.4 (C-6), 71.1, 65.9 (C-4), 57.1 (C-1), 52.3 (C-40), 45.8 (C-7), 41.8 (C-20), 37.4 (C-17), 28.3 (C-5), 28.0 (C-8), 25.0 (C-19), 19.8 (C-18), and 8.6 (C-37); Anal. for C_38_H_40_N_8_O_2_; Calcd: C, 71.23; H, 6.29; N, 17.49 Found: C, 71.25; H, 6.32; N, 17.46; [M+] *m*/*z*: 640; IR (KBr, cm^−1^): 1617 (C=N), 1684–1723 (C=O), 3427 (NH).

(*1’S,2’R,3S*)-2’-(1-((1*H*-Benzo[*d*]imidazol-2-yl)methyl)-5-methyl-1*H*-1,2,3-triazole-4-carbonyl)-1’-(3-bromophenyl)-1’,2’,4a’,5’,6’,7’,8’,8a’,9’,9a’-decahydrospiro[indoline-3,3’-pyrrolo[1,2-*a*]indol]-2-one **8m**.


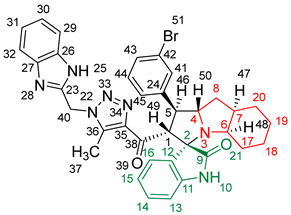


Yield: 87%; m.p.: 181–183 °C; a yellow, solid compound; ^1^H-NMR (500 MHz, DMSO-*d*_6_) *δ* 12.43 (s, 1H, NH), 9.89 (s, 1H, NH), 7.53 (t, *J* = 1.9 Hz, 2H, ArH), 7.45 (s, 1H, ArH), 7.36 (m, 3H, ArH), 7.24 (t, *J* = 7.8 Hz, 2H, ArH), 7.19 (d, *J* = 6.2 Hz, 1H, ArH), 6.90–6.87 (m, 1H, ArH), 6.80 (t, *J* = 7.5 Hz, 1H, ArH), 6.28 (d, *J* = 8.8 Hz, 1H, ArH), 5.69 (s, 2H, CH_2_), 4.97 (d, *J* = 12.2 Hz, 1H, COCH), 4.02 (q, *J* = 8.2 Hz, 1H), 3.86–3.80 (m, 1H), 3.16 (d, *J* = 4.3 Hz, 1H, aliphatic-H), 2.03 (d, *J* = 4.3 Hz, 1H, aliphatic-H), 1.92 (s, 3H, CH_3_), 1.88–1.86 (m, 1H, aliphatic-H), 1.50–1.46 (m, 2H, aliphatic-H), 1.39–1.37 (m, 1H, aliphatic-H), 1.28–0.65 (m, 6H, aliphatic-H); ^13^C-NMR (126 MHz, DMSO-*d*_6_) *δ* 191.4 (C-38), 180.2 (C-9), 148.1 (C-12), 143.2 (C-36), 143.1(C-23), 142.5 (C-11), 138.6 (C-24), 131.4 (C-27), 131.1 (C-26), 130.2 (C-15), 129.4 (C-24), 128.2 (C-35), 126.9 (C-44), 124.1 (C-43), 122.3 (C-45), 120.7 (C-14), 119.5 (C-31, C-30), 109.6 (C-2), 71.2 (C-6), 66.1 (C-4), 57.2 (C-1), 52.7 (C-40), 52.4, 45.8 (C-7), 41.67 (C-20), 38.36, 36.82 (C-17), 34.30, 30.91, 29.52, 28.64, 28.21 (C-5), 27.96 (C-8), 27.60, 24.93 (C-19), 19.8 (C-18), and 8.5 (C-37); Anal. for C_36_H_34_BrN_7_O_2_; Calcd: C, 63.91; H, 5.07; N, 14.49 Found: C, 63.89; H, 5.10; N, 14.45; [M+] *m*/*z*: 675; IR (KBr, cm^−1^): 1616 (C=N), 1681–1724 (C=O), 3428 (NH).

(*1’S,2’R,3S*)-2’-(1-((1*H*-Benzo[*d*]imidazol-2-yl)methyl)-5-methyl-1*H*-1,2,3-triazole-4-carbonyl)-1’-(3,4,5-trimethylphenyl)-1’,2’,4a’,5’,6’,7’,8’,8a’,9’,9a’-decahydrospiro[indoline-3,3’-pyrrolo[1,2-*a*]indol]-2-one **8n**.


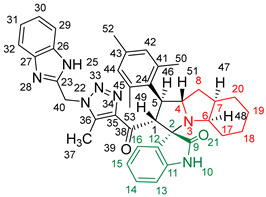


Yield: 86%; m.p.: 179–181 °C; a yellow, solid compound; ^1^H-NMR (400 MHz, DMSO-*d*_6_) *δ* 12.46 (s, 1H, NH), 9.94 (s, 1H, NH), 7.57 (d, *J* = 7.3 Hz, 1H, ArH), 7.46 (d, *J* = 8.1 Hz, 1H, ArH), 7.32 (d, *J* = 4.4 Hz, 1H, ArH), 7.14 (t, *J* = 7.3 Hz, 2H, ArH), 6.92 (t, *J* = 7.3 Hz, 1H, ArH), 6.87 (t, *J* = 7.3 Hz, 1H, ArH), 6.79 (s, 1H, ArH), 6.74 (s, 1H, ArH), 6.34 (d, *J* = 7.3 Hz, 1H, ArH), 5.72 (s, 2H, CH_2_), 4.47 (d, *J* = 4 Hz, 1H, COCH), 4.44 (q, *J* = 8.2 Hz, 1H), 4.31 (t, *J* = 11.4 Hz, 1H), 3.21 (d, *J* = 4.3 Hz, 1H, aliphatic-H), 2.66 (s, 3H, CH_3_), 2.61 (s, 3H, CH_3_), 2.19 (m, 1H, aliphatic-H), 2.13 (s, 3H, CH_3_), 2.09 (d, *J* = 5.1 Hz, 1H, aliphatic-H), 1.97 (s, 3H, CH_3_), 1.62–1.49 (m, 3H, aliphatic-H), 1.44 (m, 1H, aliphatic-H), 1.34 (m, 2H, aliphatic-H), 1.21 (d, *J* = 7.3 Hz, 1H, aliphatic-H), and 0.81 (d, *J* = 11.7 Hz, 2H, aliphatic-H); ^13^C-NMR (101 MHz, DMSO-*d*_6_) *δ* 192.7 (C-38), 180.6 (C-9), 148.1 (C-12), 143.3 (C-36), 142.6 (C-23), 138.5 (C-11), 136.4 (C-27), 135.6 (C-26), 134.9 (C-24), 132.2 (C-45, C-41), 131.6 (C-43), 129.7 (C-15), 129.2 (C-35), 128.6 (C-44, C-42), 128.2 (C-14), 127.0 (C-31, C-30), 124.6 (C-16), 123.2 (C-32, C-29), 119.5 (C-13), 109.6 (C-2), 72.0 (C-6), 67.9 (C-4), 63.5 (C-1), 57.3 (C-40), 52.8 (C-7), 47.8, 45.8 (C-20), 41.8 (C-17), 28.5 (C-5), 27.8 (C-8), 24.8 (C-19), 23.0 (C-18), 22.2 (C-52), 21.8 (C-51, C-53), and 8.7 (C-37); Anal. for C_39_H_41_N_7_O_2_; Calcd: C,73.21; H,6.46; N,15.32 Found: C, 73.32; H, 6.41; N, 15.29; [M+] *m*/*z*: 639; IR (KBr, cm^−1^): 1617 (C=N), 1682–1723 (C=O), 3428 (NH).

### 3.2. Computational Protocol

Computational protocol has been provided in the Supplementary Materials.

## 4. Conclusions

A new set of spirooxindoles with different pharmacophores like benzimidazole, triazoles, and isatin moieties were achieved via the 32CA reaction between the in situ generated AY and the synthesized chalcones containing a wide range of substituents. The final spirooxindoles were obtained with total selectivity and at an up to 90% yield, yielding only one of the possible isomeric products. The X-ray crystal structure of triazole-benzimdiazole **4** was identified. Several spirooxindoles molecules were created, whose final chemical architectures with different electronic effects constitute material for future studies.

The mechanism of the 32CA reactions between AY **9** and the simplest chalcone **5a** was theoretically studied by means of MEDT at the *ω*B97X-D/6-311g(d,p) DFT level. Out of the 16 possible isomeric reaction paths, the reported *ortho*/*endo* path leading to **8a** via **TS-on** is the most favorable one, with a very low activation Gibbs free energy of 11.1 kcal·mol^−1^ and a strong exergonic character of 24.0 kcal·mol^−1^. The formation of spirooxindole **8a** is completely selective because the other competitive isomeric reaction paths are at least 2.3 kcal·mol^−1^ higher in energy.

This low activation energy is a consequence of the supernucleophilicity of AY **9** and the strong electrophilicity of **5a**, which favor a highly polar 32CA reaction of FEDF, as characterized by the high GEDT computed at the most-favorable **TS-on**.

Finally, the geometrical analysis of **TS-on** and the corresponding vibrational modes observed indicate that the polar 32CA reaction follows a *two-stage, one-step* mechanism in which the formation of the C3–C4 single bond involving the β-conjugated C4 carbon of the chalcone derivative is more advanced. The present combined experimental and theoretical MEDT study reports the synthesis of new spirooxindoles with promising biological activity and sheds light on the mechanistic aspects of the key 32CA reaction step, with the aim of achieving a wider set of this relevant type of compound and a better understanding of the processes for potential future designs.

## Figures and Tables

**Table 1:** Crystal data and structural refinement data for 1,2,3-triazolyl ketone (**4**).

Compound	4
Empirical formula	C_13_H_13_N_5_O
Formula weight	255.28
Temperature	102(2) K
Wavelength	1.54178 Å
Crystal system	Tetragonal
Space group	*P(4)3*
Unit-cell dimensions	*a* = 8.1744(2) Å *σ* = 90 *b* = 8.1744(2) Å *β* = 90° *c* = 19.3863(8) Å *δ* = 90°.
Volume	1295.41(8) Å^3^
Z	4
Density (calculated)	1.309 Mg/m^3^
Absorption coefficient	0.724 mm^−1^
F(000)	536
Crystal size	0.280 × 0.070 × 0.040 mm^3^
Theta range for data collection	5.411 to 67.814°
Index ranges	−9 ≤ h ≤ 9, −9 ≤ k ≤ 9, −22 ≤ l ≤ 23
Reflections collected	10,956
Independent reflections	2023 [R(int) = 0.0591]
Completeness to theta = 67.679°	86.8%
Absorption correction	Semi-empirical from equivalents
Refinement method	Full-matrix least-squares on *F*^2^
Data/restraints/parameters	2023/1/180
Goodness-of-fit on *F*^2^	1.061
Final R indices [I > 2sigma(I)]	*R*_1_ = 0.0319, *wR*_2_ = 0.0762
R indices (all data)	*R*_1_ = 0.0337, *wR*_2_ = 0.0771
Absolute structure parameter	0.4(4)
Extinction coefficient	0.0147(16)
Largest diff. peak and hole	0.172 and −0.186 e.Å^−3^

**Table 2:** Hydrogen bonds of 1,2,3-triazolyl ketone (**4**). Distances are given in angstroms Å, and angles are given in degrees °.

D–H···A	d(D–H)	d(H···A)	d(D···A)	<(DHA)
N1–H2A···N2	0.87(4)	2.01(4)	2.878(3)	178(4)
C8–H8AB···O1	0.99	2.50	3.029(3)	113
C8–H8AB···N4	0.99	2.57	3.400(3)	142
C4–H4···O1	0.95	2.58	3.448(4)	151
C11–H11B···01	0.98	2.54	3.189(4)	123
C11–H11C···N5	0.98	2.53	3.388(4)	146

**Table 3:** *ω*B97X-D/6-311G(d,p) gas-phase electronic chemical potential (*μ*), chemical hardness (*η*), electrophilicity (ω), and nucleophilicity (*N*) indices, in eV, of AY **9** and chalcone **5a**.

	*μ*	*η*	ω	*N*
Chalcone **5a**	−4.46	7.97	1.25	2.96
AY **9**	−2.92	6.92	0.61	5.02

## Data Availability

The data presented in this study are available in this article and Supplementary Materials.

## Supplementary Materials

The following supporting information can be downloaded at https://www.mdpi.com/article/10.3390/molecules28196976/s1. General remarks about solvents, chemical reagents, and instrumentations; Computational protocol [21,22,25,31,33,34,35,36,37,38,39,40,41,42,43,44,45,46,47,48,49]; Figures S1–S31: NMR and IR spectrum; Figure S32: reaction mechanism roadmap of the competitive isomeric reaction paths in the 32CA reaction of AY **9** with chalcone **5a**; Figure S33: *ω*B97X-D/6-311G(d,p) IRC path associated with the most-favorable ortho/endo reaction path via **TS-on** in methanol.; Table S1: *ω*B97X-D/6-311G(d,p) enthalpies, entropies, and Gibbs free energies, as well as the relative versions of these measures with respect to the separated reagents, computed at 60 °C and 1 atm in methanol, for the stationary points involved in the 32CA reaction of AY **9** with chalcone **5a**; Cartesian coordinates and electronic energies of the stationary points involved in the 32CA reaction between AY **9** and chalcone **5a** in methanol. Imaginary frequencies for TSs at 60 °C are included.
