# Supplementary material for: Synthesis and Characterization of New Spirooxindoles Including Triazole and Benzimidazole Pharmacophores via [3+2] Cycloaddition Reaction: An MEDT Study of the Mechanism and Selectivity
Source: Molecules. 2023 Oct 8;28(19):6976. doi: 10.3390/molecules28196976 (PMC10574610; doi:10.3390/molecules28196976)
Supplement: Supplementary file 1 [file molecules-28-06976-s001.zip › molecules-2563520-supplementary.pdf]

Supplementary Material

Synthesis and Characterization of New Spirooxindoles including Triazole and Benzimidazole Pharmacophores via [3+2] Cycloaddition Reaction: A MEDT Study of the Mechanism and Selectivity

Saeed Alshahrani [1], Abdullah Mohammed Al-Majid [1], Abdullah Saleh Alamary [1], M. Ali [1], Mezna Saleh Altowyan [2], Mar Ríos-Gutiérrez [3], Sammer Yousuf [4] and Assem Barakat [1,*]

[1] Department of Chemistry, College of Science, King Saud University, P.O. Box 2455, Riyadh 11451, Saudi Arabia; chemistry99y@gmail.com (S.A.); amajid@ksu.edu.sa (A.M.A.-M.);
alamary1401@yahoo.com (A.S.A.); maly.c@ksu.edu.sa (M.A.)

[2] Department of Chemistry, College of Science, Princess Nourah bint Abdulrahman University,
P.O. Box 84428, Riyadh 11671, Saudi Arabia; msaltowyan@pnu.edu.sa

[3] Department of Organic Chemistry, University of Valencia, Dr. Moliner 50, 46100 Burjassot, Valencia, Spain; m.mar.rios@uv.es

[4] H.E.J. Research Institute of Chemistry, International Centre for Chemical and Biological Sciences,
University of Karachi, Karachi 75270, Pakistan; dr.sammer.yousuf@gmail.com

* Correspondence: ambarakat@ksu.edu.sa

Index

S2 General remarks about solvents, chemical reagents and instrumentations.

S7 Computational Protocol.

S8 References.

S10 NMR and IR spectrum for the synthesized compounds.

S38 **Figure S32.** Reaction mechanism roadmap of the competitive isomeric reaction paths in the 32CA reaction of AY **9** with chalcone **5a.**

S39 **Figure S33** with the *ω*B97X-D/6-311G(d,p) IRC path associated with the most favourable *ortho/endo* reaction path via **TS-on** in methanol.

S39 **Table S1** with *ω*B97X-D/6-311G(d,p) enthalpies, entropies, and Gibbs free energies, as well as the relative ones with respect to the separated reagents, computed at 60 ºC and 1 atm in methanol, for the stationary points involved in the 32CA reaction of AY **9** with chalcone **5a**.

S40 Cartesian coordinates and electronic energies of the stationary points involved in the 32CA reaction between AY **9** and chalcone **5a** in methanol. Imaginary frequencies for TSs at 60 ºC are included.

General Remarks

Solvents and Reagents

The origin of the solvents and reagents used in this work is shown in tables below which were used without further purification unless otherwise stated.

Origin and purity of solvents used.			
**Solvents**	**Empirical formula**	**CAS number**	**Provider**
Acetic acid	$C_2H_4O_2$	64-19-7	Carlo Erba
Acetonitrile	C_2H_3N	75-05-8	Carlo Erba
Chloroform 99.9%	$CHCl_3$	67-66-3	Carlo Erba
Chloroform (NMR) TMS 99.8%	$CDCl_3$	865-49-6	Eurisotop
Dichloromethane 99.9 %	CH_2Cl_2	75-09-2	Carlo Erba
Diethyl ether 99.8 %	$C_4H_{10}O$	60-29-7	Carlo Erba
Distillated water	H_2O	7732-18-5	-
DMF anhydrous 99.8 %	C_3H_7NO	68-12-2	Acros Organics
DMSO-d_6 (NMR) TMS 99.8 %	C_2D_6SO	2206-27-1	Eurisotop
Ethanol absolute 99.8 %	C_2H_6O	64-17-5	Carlo Erba
Ethyl acetate	$C_4H_8O_2$	141-78-6	Carlo Erba
Hexane	C_6H_{14}	110-54-3	Carlo Erba
Hydrochloric acid 37 %	HCl	7647-01-0	Carlo Erba

Methanol 99.8 %	CH_4O	67-56-1	Carlo Erba
Petroleum spirit 99.9 %	-	64742-49-0	Carlo Erba
Sulfuric acid 98 %	H_2SO_4	7664-93-9	Carlo Erba

Origin and purity of reagents used.

Reagents	Empirical formula	CAS number	Provider
Orthophenylenediamine	$C_6H_8N_2$	95-54-5	TCI
Potassium dichromate (VI)	$K_2Cr_2O_7$	7778-50-9	Sigma-Aldrich
Ammonia solution	NH_4OH	7664-41-7	Sigma-Aldrich
4-Chlorobenzaldehyde	C_7H_5ClO	104-88-1	Sigma-Aldrich
Benzaldehyde	C_7H_6O	100-52-7	Sigma-Aldrich
4-Methylbenzaldehyde	C_8H_8O	104-87-0	Sigma-Aldrich
4-(Trifluoromethyl) benzaldehyde	$C_8H_5F_3O$	455-19-6	Sigma-Aldrich
4-Nitrobenzaldehyde	$C_7H_5NO_3$	555-16-8	Sigma-Aldrich
2,4-Dichlorobenzaldehyde	$C_7H_4Cl_2O$	874-42-0	Sigma-Aldrich
Thiophene-2-carbaldehyde	C_5H_4OS	98-03-3	Sigma-Aldrich
4-Fluorobenzaldehyde	C_7H_5FO	459-57-4	Sigma-Aldrich
3-Nitrobenzaldehyde	$C_7H_5NO_3$	99-61-6	Sigma-Aldrich
3,4,5-trimethoxybenzaldehyde	$C_{10}H_{12}O_4$	86-81-7	Sigma-Aldrich
4-Dimethylaminobenzaldehyde	$C_9H_{11}NO$	100-10-7	Sigma-Aldrich
4-Bromobenzaldehyde	C_7H_5BrO	1122-91-4	Sigma-Aldrich

2-Furaldehyde	$C_5H_4O_2$	98-01-1	Sigma-Aldrich
3-Fluorobenzaldehyde	C_7H_5FO	456-48-4	Sigma-Aldrich
(2S,3aS,7aS)-Octahydroindole-2-carboxylic acid	$C_9H_{15}NO_2$	80875-98-5	Sigma-Aldrich
Isatin	$C_8H_5NO_2$	91-56-5	Sigma-Aldrich
Sodium hydroxide	NaOH	1310-73-2	Sigma-Aldrich
Potassium hydroxide	KOH	1310-58-3	Sigma-Aldrich
Sodium Sulfate Anhydrous	Na_2SO_4	7757-82-6	Sigma-Aldrich
Sodium hydrogen carbonate	$NaHCO_3$	144-55-8	Sigma-Aldrich
L-proline	$C_5H_9NO_2$	147-85-3	Sigma-Aldrich
α-Chloroacetic acid	$C_2H_3ClO_2$	79-11-8	Sigma-Aldrich

Chromatography

Analytical thin layer chromatography (TLC)

Silica plates (Kieselger 60 F254, thickness 0.2 mm, Merck) are used for thin layer chromatography. Plates revelation is done by direct observation for colored compounds or under ultraviolet light (λ = 254 nm or λ = 365 nm) for conjugated compounds. The various eluents used are specified in the synthesis chapter after each experimental protocol. The ratio indicated are by volume.

Column chromatography

Purifications were performed with columns from 2 to 5 centimeters in diameter, packed on 20 to 40 centimeters tall with silica (Silica Gel 60, granulometry 0.015 to 0.040 mm, Merck) dispersed in the eluent mixture selected. In order to be purified, all crude products are dissolved in a minimum of starting eluent or fixed on Florisil (60-100 mesh, VWR) and deposited in the column heading.

Drying and Evaporation of Organic Solvents and reagents.

Organic solvents were dried by standing over anhydrous magnesium sulfate unless otherwise stated. Solutions were evaporated under reduced pressure on a Büchi rotary evaporator using a water pump vacuum unless otherwise stated.

Physico-chemical analysis.

Infrared spectroscopy.

IR spectra were performed on a Nicolet 6700 FT-IR spectrophoto- meter, on samples conditioned in potassium bromide pellet (1-2 wt%). The wave numbers are given in cm^{-1}.

NMR spectroscopy

The NMR spectra were recorded using a Varian Mercury Jeol-400 and Jeol-500 NMR spectrometer. ^{1}H-NMR (400 MHz) and ^{13}C-NMR (101 MHz) and ^{1}H-NMR (500 MHz) and ^{13}C-NMR (126 MHz) spectroscopy were performed in either deuterated dimethylsulfoxide (DMSO-d_6) or

deuterated chloroform ($CDCl_3$). Chemical shifts (δ) are reported in terms of ppm and coupling constants J are given in Hz.

Organic elemental analysis

Elemental analysis was carried out using an Elmer 2400 Elemental Analyzer in CHN mode.

Computational Protocol

All calculations were performed using the ωB97X-D [1] functional together with the standard 6-311G(d,p) [2] basis set. This functional includes long-range exchange and semi classical London dispersion corrections, and has demonstrated to be an efficient method in the study of organic reactions. [3] The TSs were characterized by the presence of only one imaginary frequency. The Berny method was used in optimizations. [4,5] The intrinsic reaction coordinates (IRC) paths [6] were traced to obtain the energy profiles connecting each TS to the two associated minima in the potential energy surface using the Hratchian-Schlegel Hessian-based Predictor-Corrector integrator [7-9].

Solvent effects of methanol in the thermodynamic calculations were taken into account by full optimization of the gas-phase structures at the same computational level using the polarizable continuum model (PCM) [10,11] in the framework of the self-consistent reaction field (SCRF). [12-14] Values of ωB97X-D/6-311G(d,p) enthalpies, entropies, and Gibbs free energies in methanol were calculated with standard statistical thermodynamics at 61 °C and 1 atm [2] by PCM frequency calculations at the solvent-optimized structures.

The GEDT [15] values were computed using the equation $GEDT(f) = \Sigma q_f$, where q are the natural charges [16,17] of the atoms belonging to one of the two frameworks (f) at the TS geometries. Conceptual DFT [18,19] (CDFT) indices were calculated using the equations in reference 32.

The Gaussian 16 suite of programs was used to perform the calculations. [20] Molecular geometries were visualized by using the GaussView program. [21]

References

1. Chai, J.-D.; Head-Gordon, M. Long-range corrected hybrid density functionals with damped atom–atom dispersion corrections. *Phys. Chem. Chem. Phys.* **2008**, *10*, 6615-6620.
2. Hehre, M.J.; Radom, L.; Schleyer, P.v.R.; Pople, J. Ab initio Molecular Orbital Theory, Wiley, New York, 1986.
3. Domingo, L.R.; Pérez. Lithium cation-catalyzed benzene Diels−Alder reaction: Insights on the molecular mechanism within the Molecular Electron Density Theory. *J. Org. Chem.* **2020**, *85*, 13121−13132.
4. Schlegel, H.B. Optimization of equilibrium geometries and transition structures. *J. Comput. Chem.* **1982**, *3*, 214-218.
5. Schlegel, H.B. In modern electronic structure theory, Yarkony, D.R., Ed., World Scientific Publishing, Singapore, 1994.
6. Fukui, K. Formulation of the reaction coordinate. *J. Phys. Chem.* **1970**, *74*, 4161–4163.
7. Hratchian, H.P.; Schlegel, H. B. in *Theory and Applications of Computational Chemistry: The First 40 Years*, Elsevier, Amsterdam, **2005**, pp. 195–249.
8. Hratchian, H.P.; Schlegel, H. B. Accurate reaction paths using a Hessian based predictor–corrector integrator, *J. Chem. Phys.* **2004**, *120*, 9918–9924.
9. Hratchian, H.P.; Schlegel, H. B. Using Hessian Updating To Increase the Efficiency of a Hessian Based Predictor-Corrector Reaction Path Following Method, *J. Chem. Theory Comput.* **2005**, *1*, 61–69.
10. Tomasi, J.; Persico, M. Molecular interactions in solution: and overview of methods based on continuous distributions of the solvent. *Chem. Rev.* **1994**, *94*, 2027-2094.
11. Simkin, B.Ya.; Sheikhet, I.I. Quantum chemical and statistical theory of solutions–computational approach, Ellis Horwood: London, 1995.
12. Cossi, M.; Barone, V.; Cammi, R.; Tomasi, J. Ab initio study of solvated molecules: A new implementation of the polarizable continuum model. *Chem. Phys. Lett.* **1996**, *255*, 327-335.
13. Cances, E.; Mennucci, B.; Tomasi, J. A new integral equation formalism for the polarizable continuum model: Theoretical background and applications to isotropic and anisotropic dielectrics. *J. Chem. Phys.* **1997**, *107*, 3032-3041.

14. Barone, V.; Cossi, M.; Tomasi, J. Geometry optimization of molecular structures in solution by the polarizable continuum model. *J. Comput. Chem.* **1998**, *19*, 404-417.
15. Domingo, L.R. A new C-C bond formation model based on the quantum chemical topology of electron density. *RSC Adv*. **2014**, *4*, 32415-32428.
16. Reed, A.E.; Weinstock, R.B.; Weinhold, F. Natural population analysis. *J. Chem. Phys.* **1985**, *83*, 735-746.
17. Reed, A.E.; Curtiss, L.A.; Weinhold, F. Intermolecular interactions from a natural bond orbital, donor-acceptor viewpoint. *Chem. Rev.* **1988**, *88*, 899-926.
18. Parr, R.G.; Yang, W. *Density functional theory of atoms and molecules*, Oxford University Press, New York, 1989.
19. Domingo, L.R.; Ríos-Gutiérrez, M.; Pérez, P. Applications of the conceptual density functional indices to organic chemistry reactivity. *Molecules* **2016**, *21*, 748.
20. Gaussian 16, Revision A.03, Frisch, M.J.; Trucks, G.W.; Schlegel, H.B.; Scuseria, G.E.; Robb, M.A.; Cheeseman, J.R.; Scalmani, G.; Barone, V.; Petersson, G. A.; Nakatsuji, H.; et al. Gaussian, Inc., Wallingford CT, **2016**.
21. GaussView, Version 6.0, Dennington, R.; Keith, T.A.; Millam, J.M., Semichem Inc., Shawnee Mission, KS, **2016**.

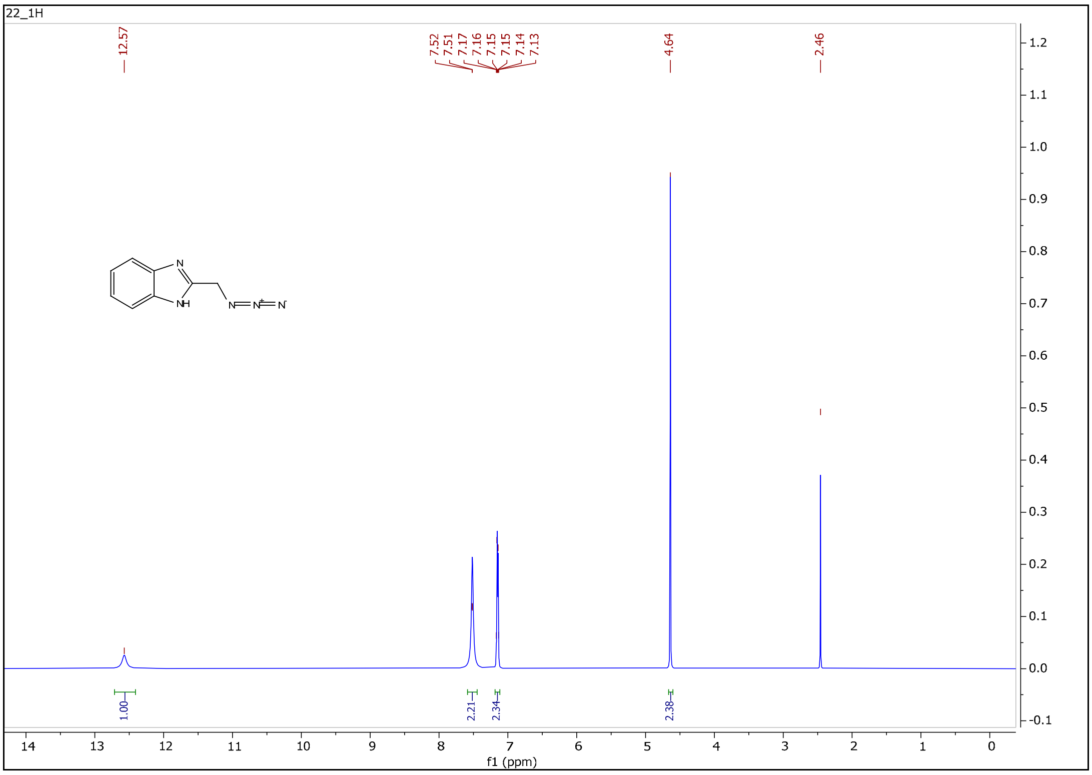

Figure S1. ^{1}H-NMR Spectrum of compound (**3**) (DMSO-d_6).

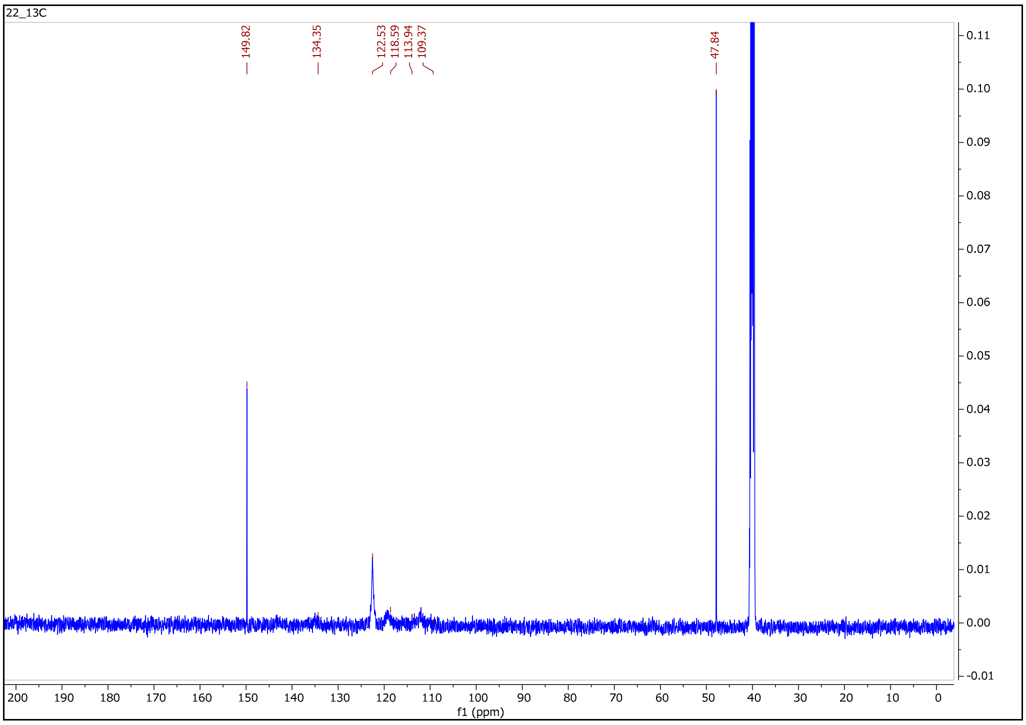

Figure S2. ^{13}C-NMR Spectrum of compound (**3**) (DMSO-d_6).

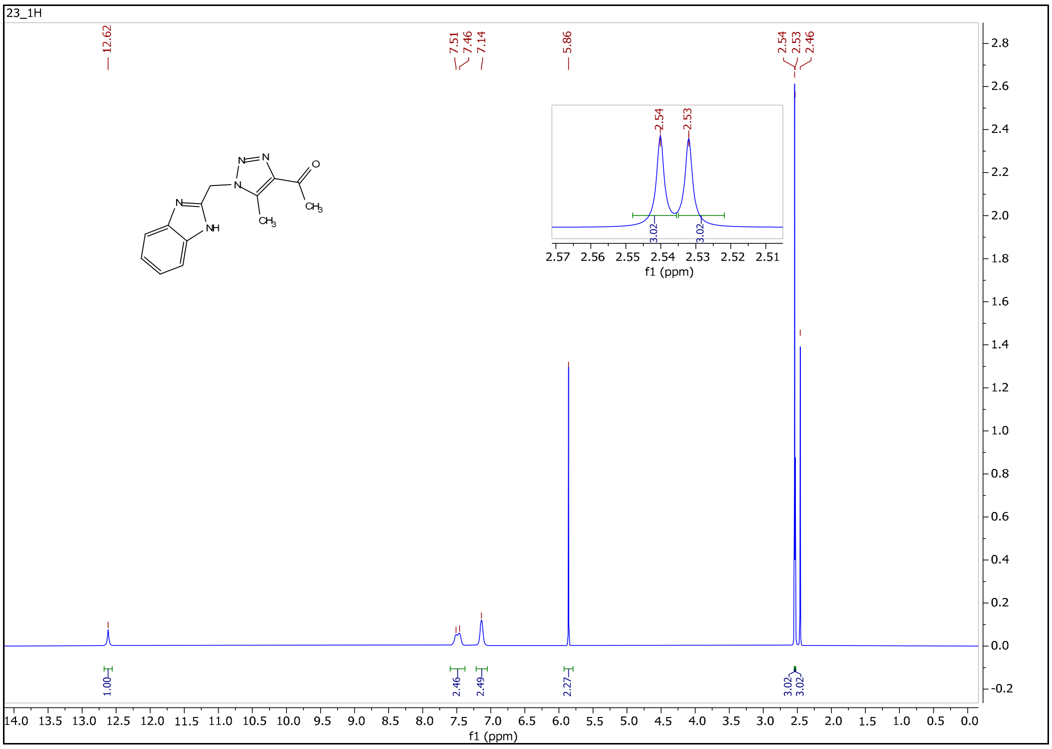

Figure S3. ^{1}H-NMR Spectrum of compound (**4**) (DMSO-d_6).

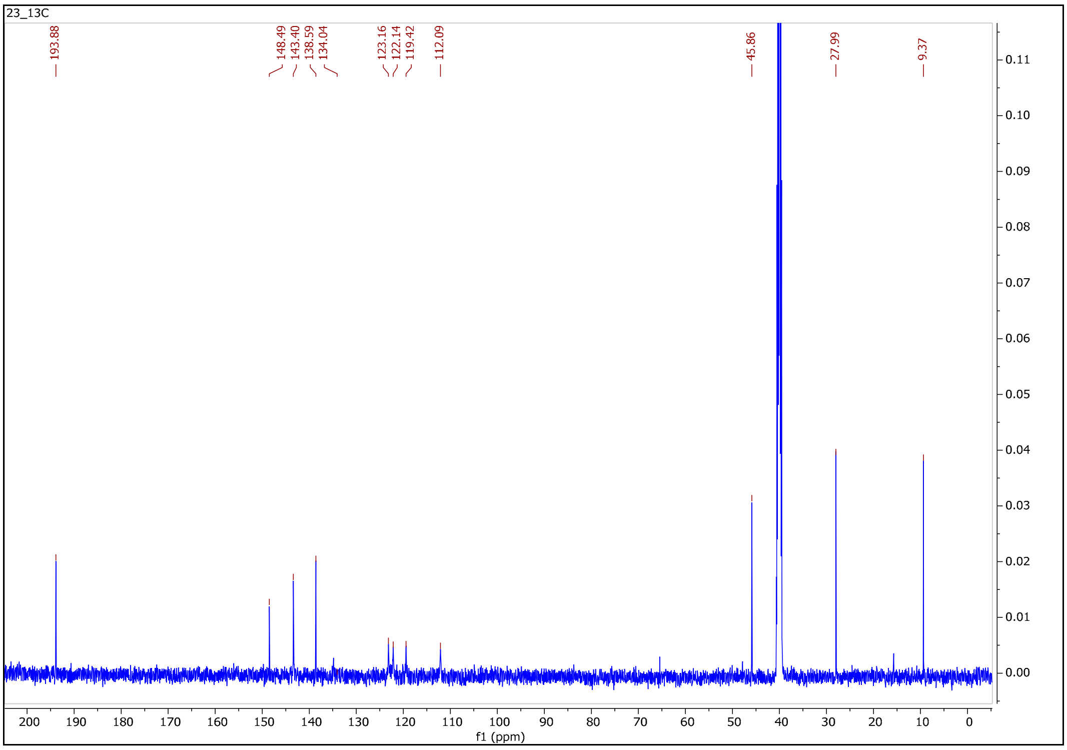

Figure S4. ^{13}C-NMR Spectrum of compound (**4**) (DMSO-d_6).

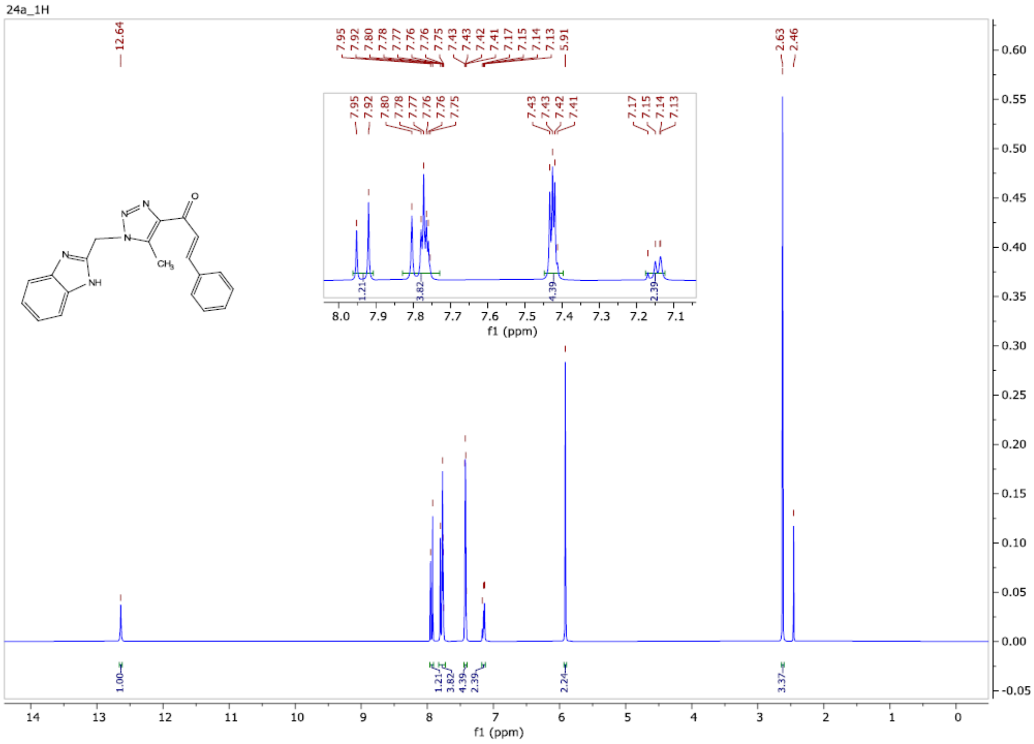

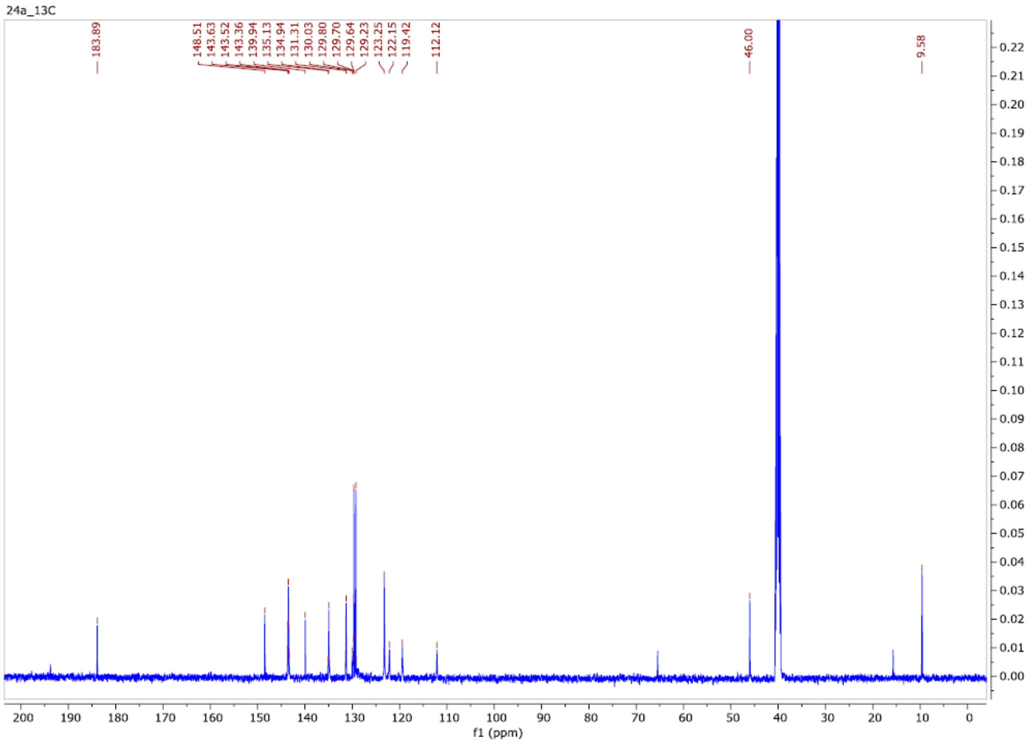

Figure S6. ^{1}H-NMR &^{13}C-NMR Spectrum of compound (**5a**) (DMSO-d_6).

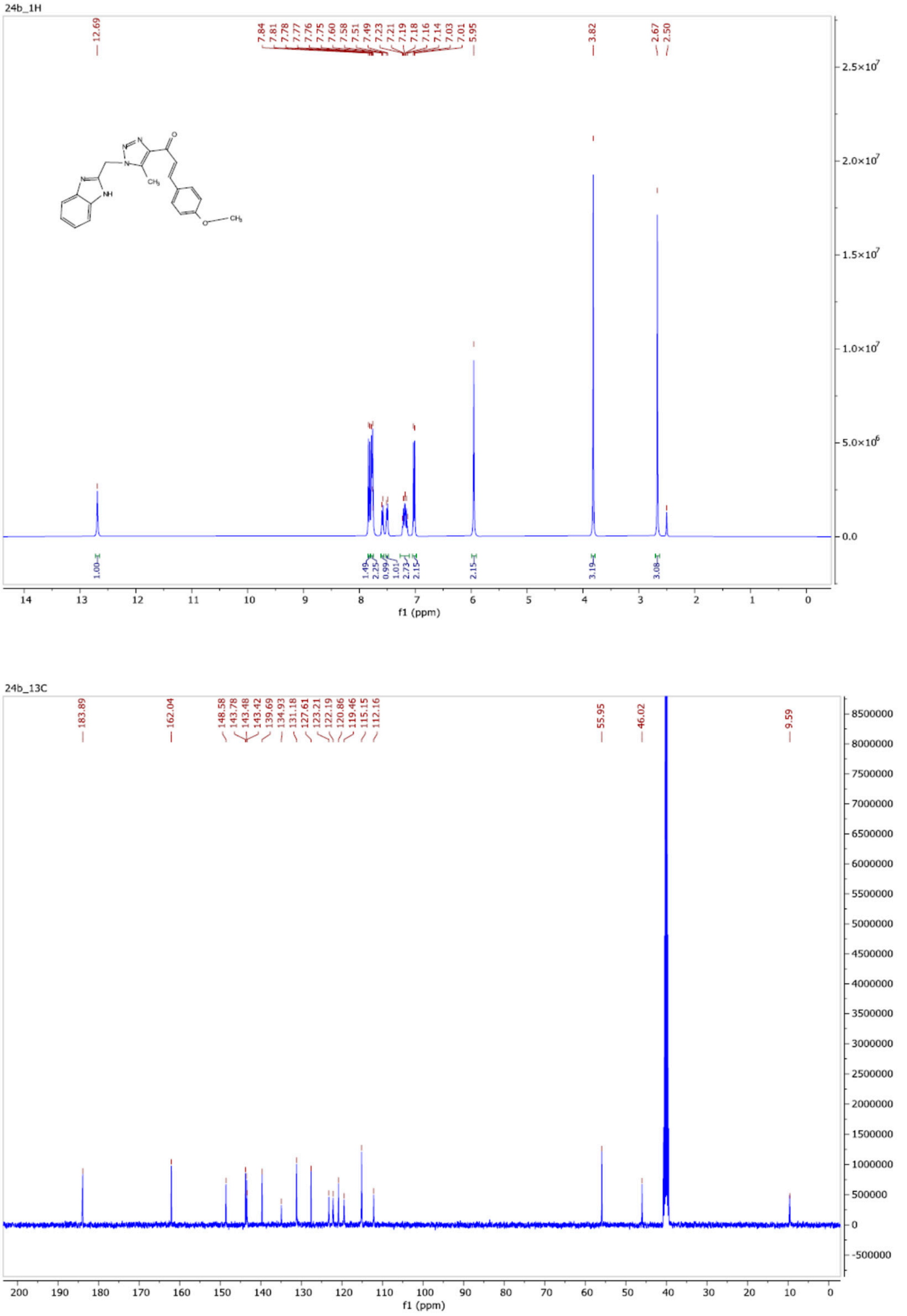

Figure S7. ^{1}H-NMR &^{13}C-NMR Spectrum of compound (**5b**) (DMSO-*d*$_6$).

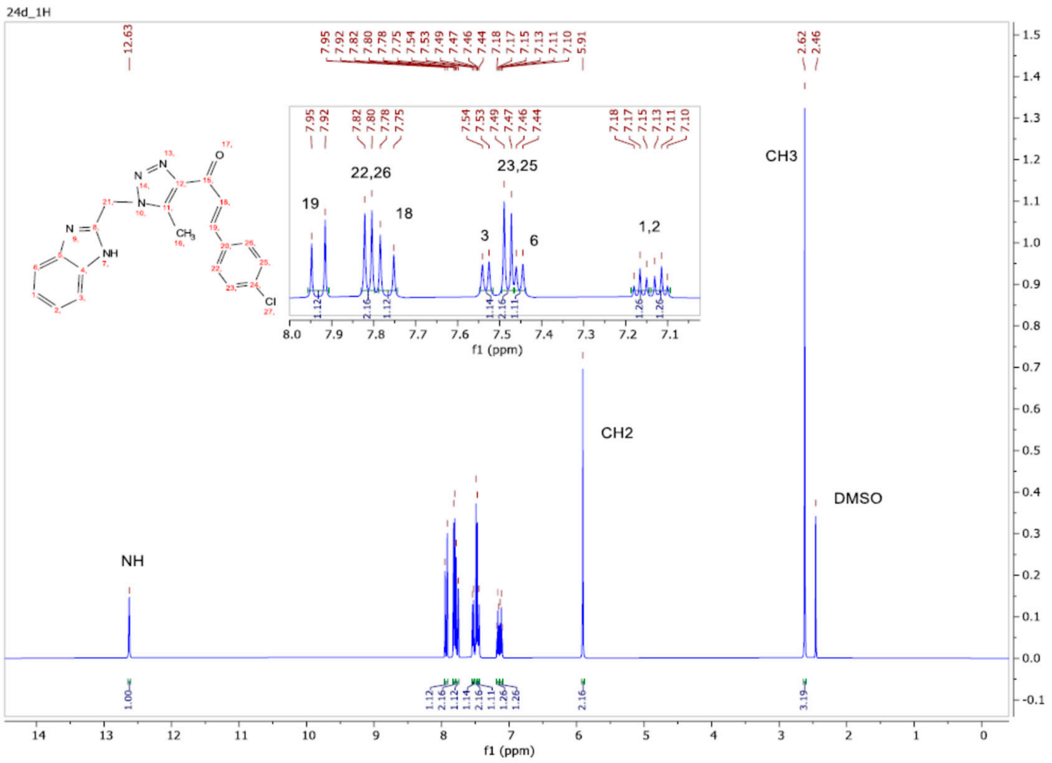

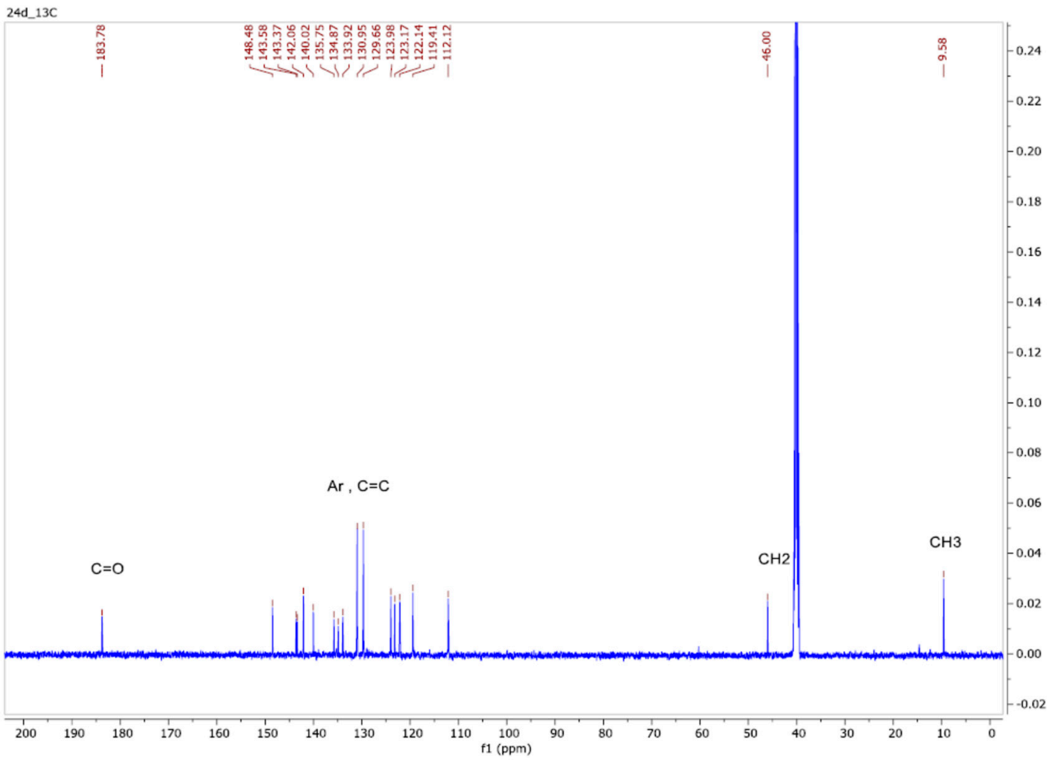

Figure S8. ^{1}H-NMR &^{13}C-NMR Spectrum of compound (**5d**) (DMSO-d_6).

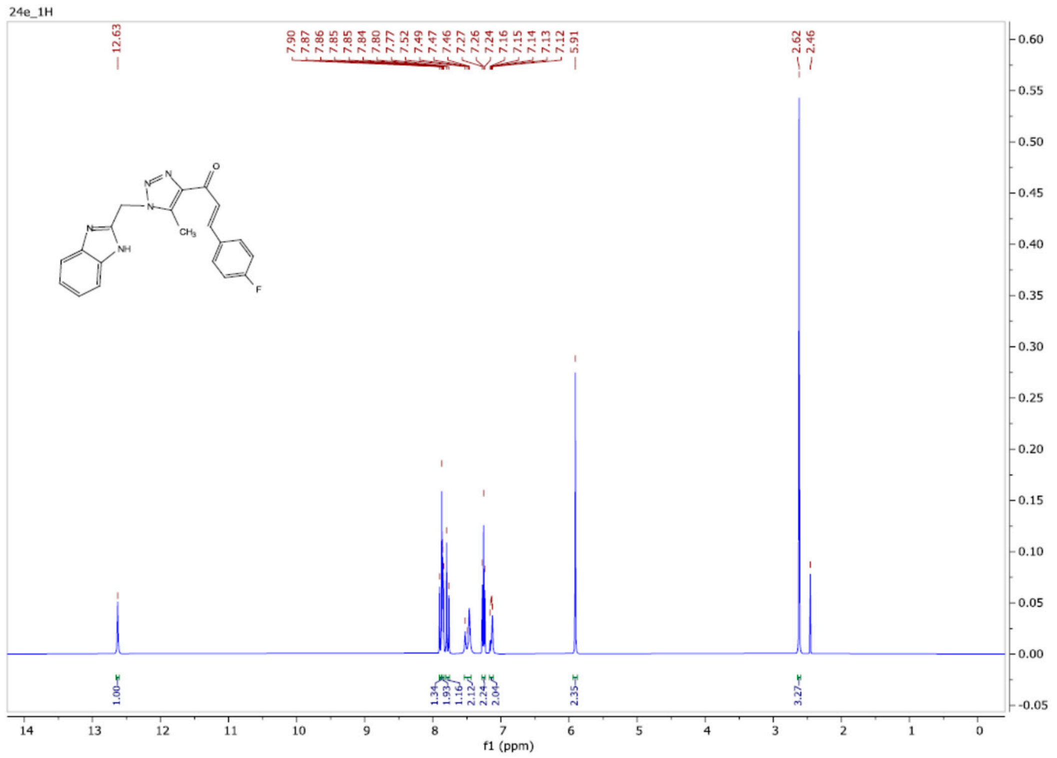

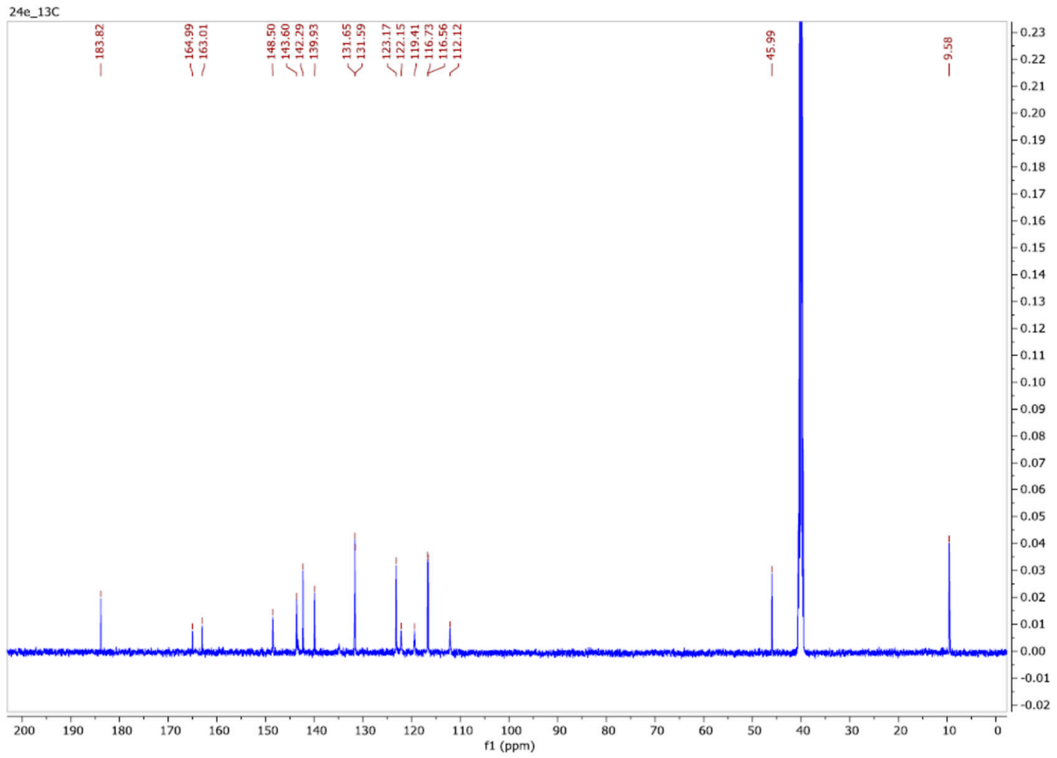

Figure S9. ^{1}H-NMR &^{13}C-NMR Spectrum of compound (**5e**) (DMSO-d_6).

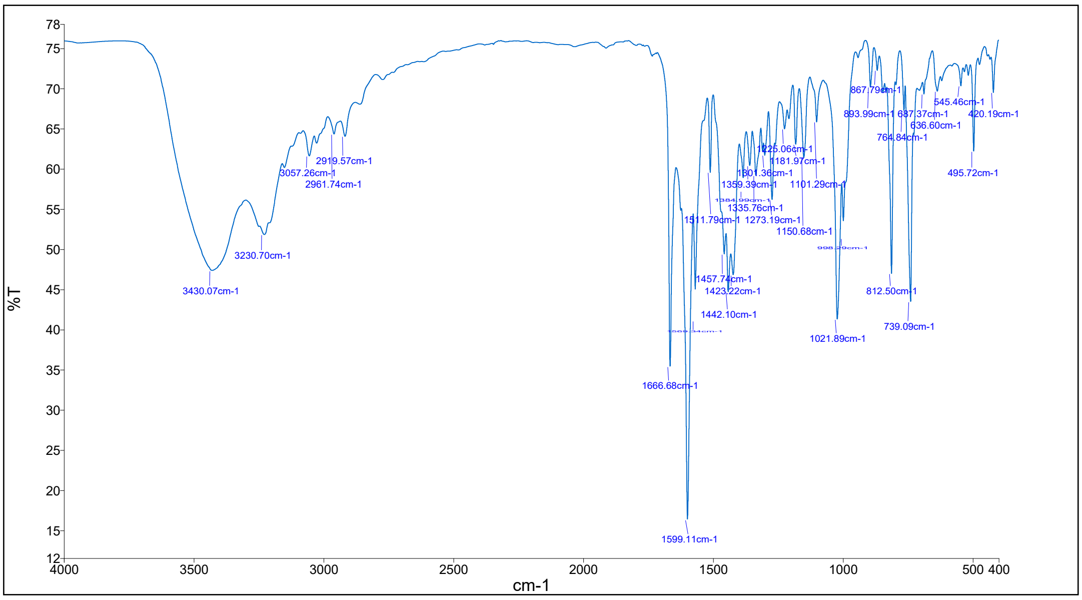

Figure S10. IR Spectrum of compound (**5f**).

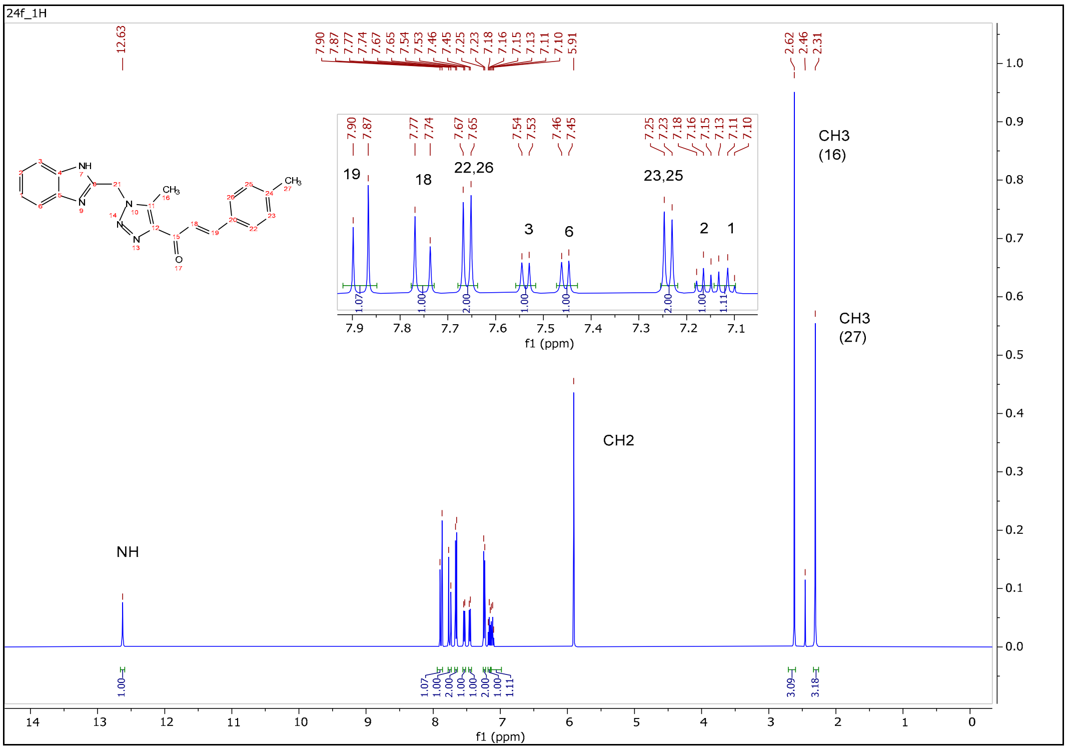

Figure S11. ^{1}H-NMR Spectrum of compound (**5f**) (DMSO-*d*6).

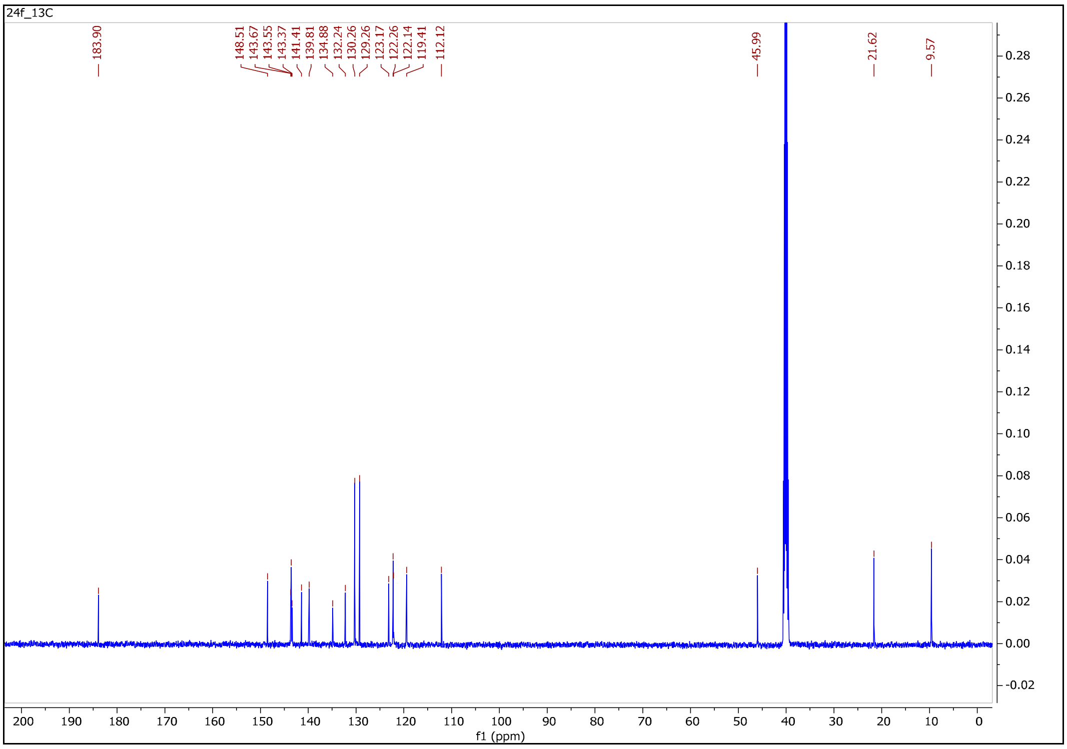

Figure S12. ^{13}C-NMR Spectrum of compound (**5f**) (DMSO-*d*6).

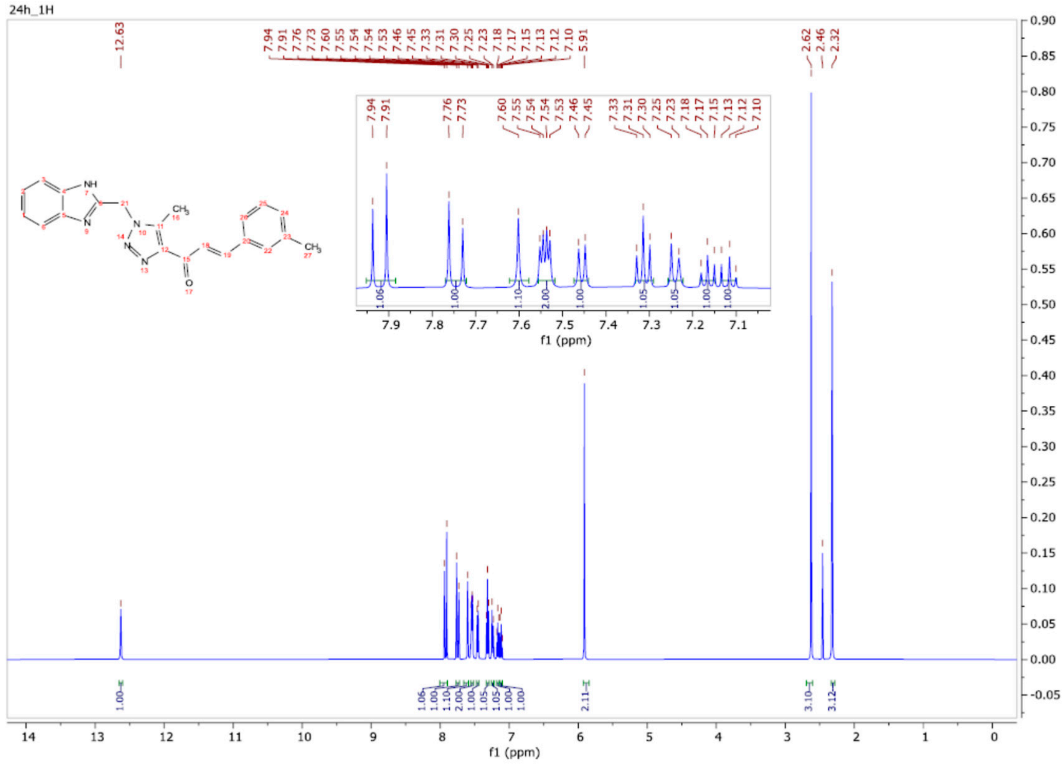

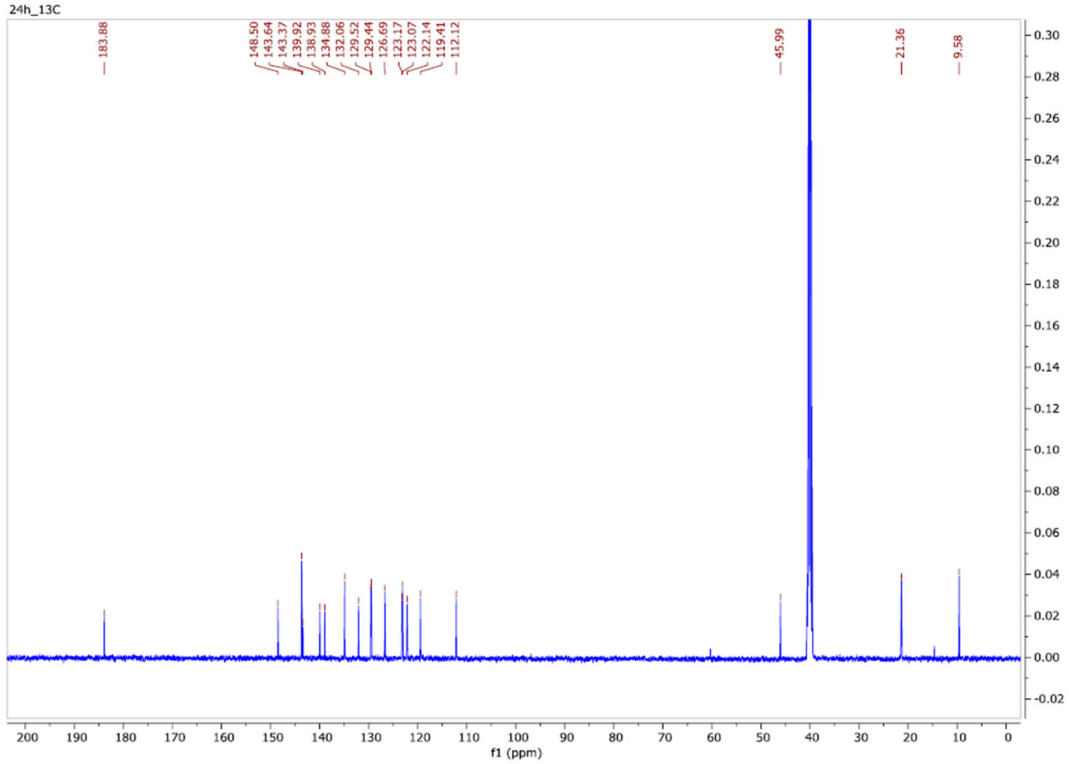

Figure S13. ^{1}H-NMR &^{13}C-NMR Spectrum of compound (**5h**) (DMSO-d_6).

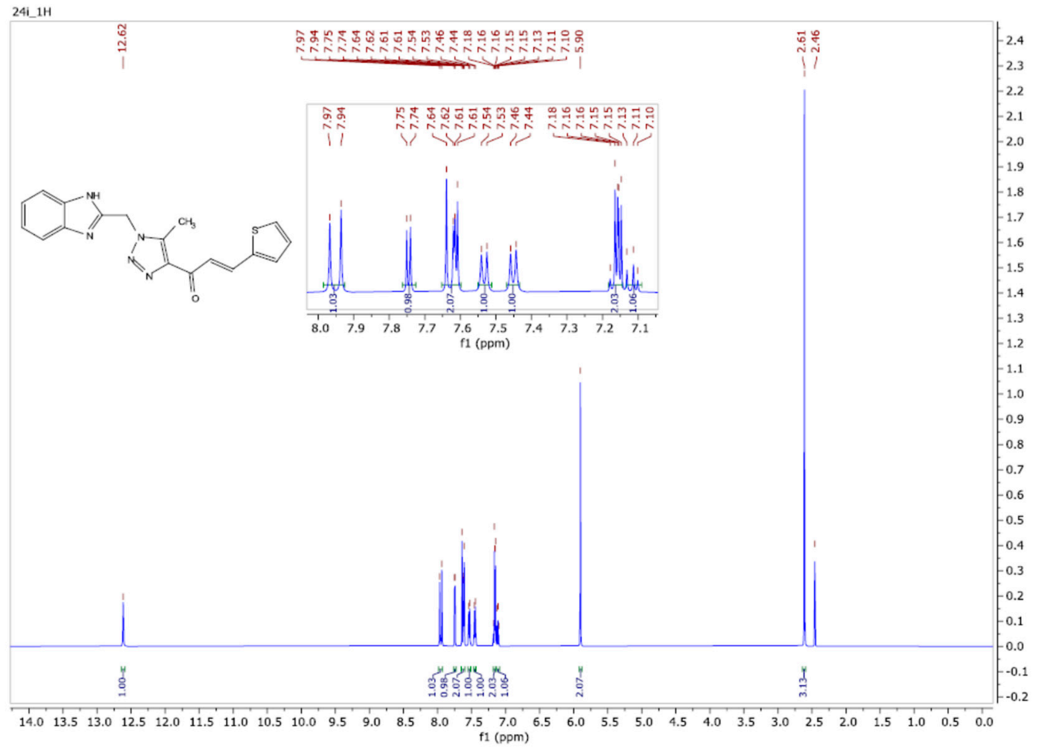

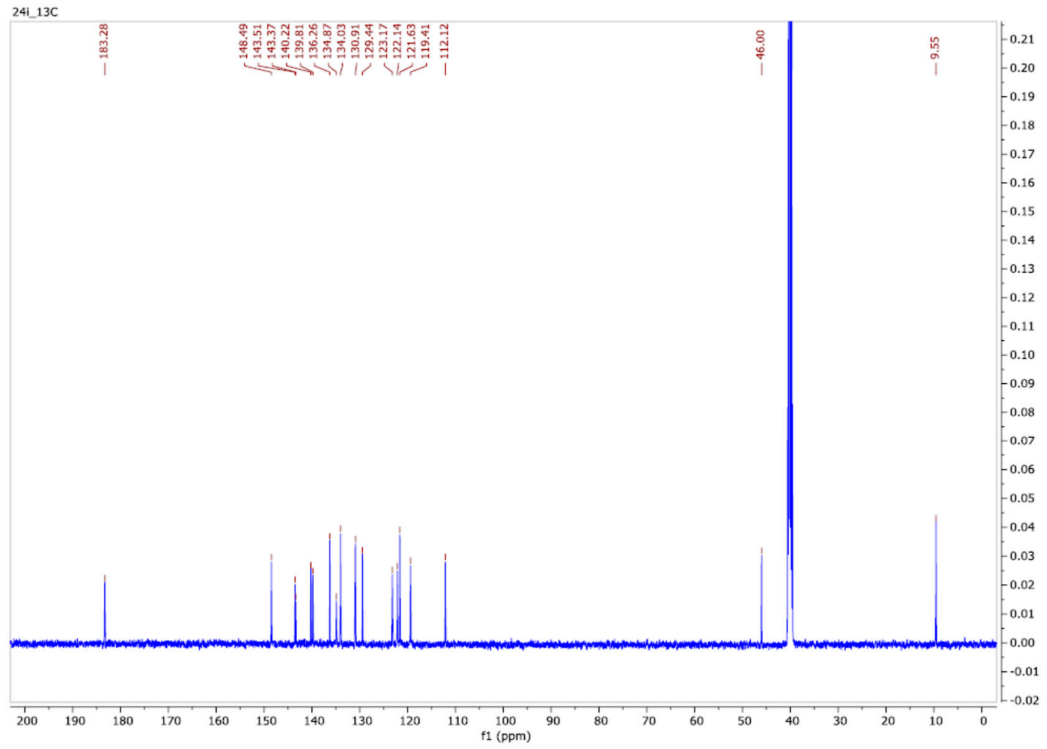

Figure S14. ^{1}H-NMR &^{13}C-NMR Spectrum of compound (**5i**) (DMSO-*d₆*).

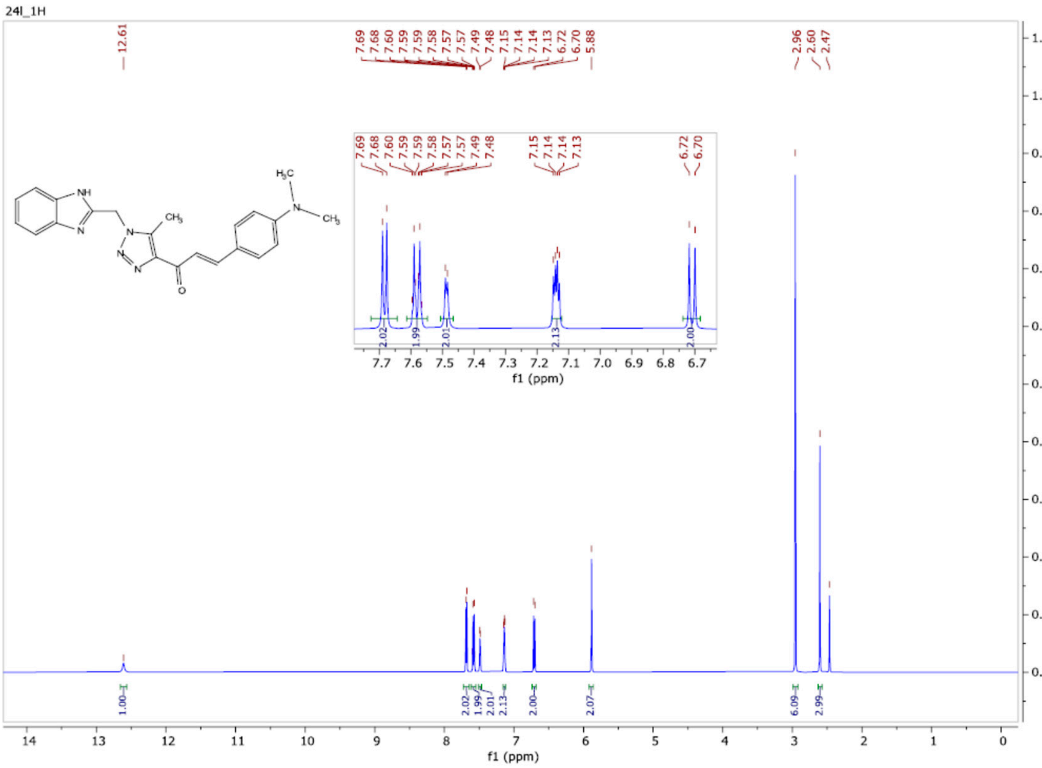

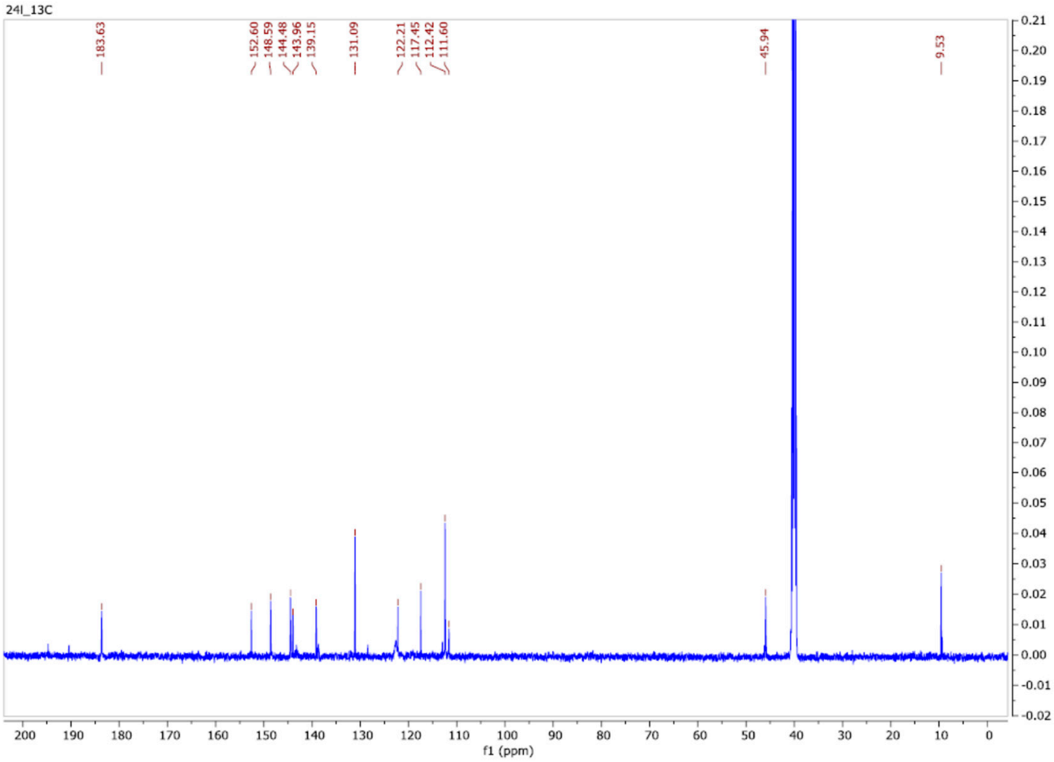

Figure S15. ^{1}H-NMR &^{13}C-NMR Spectrum of compound (**5l**) (DMSO-d_6).

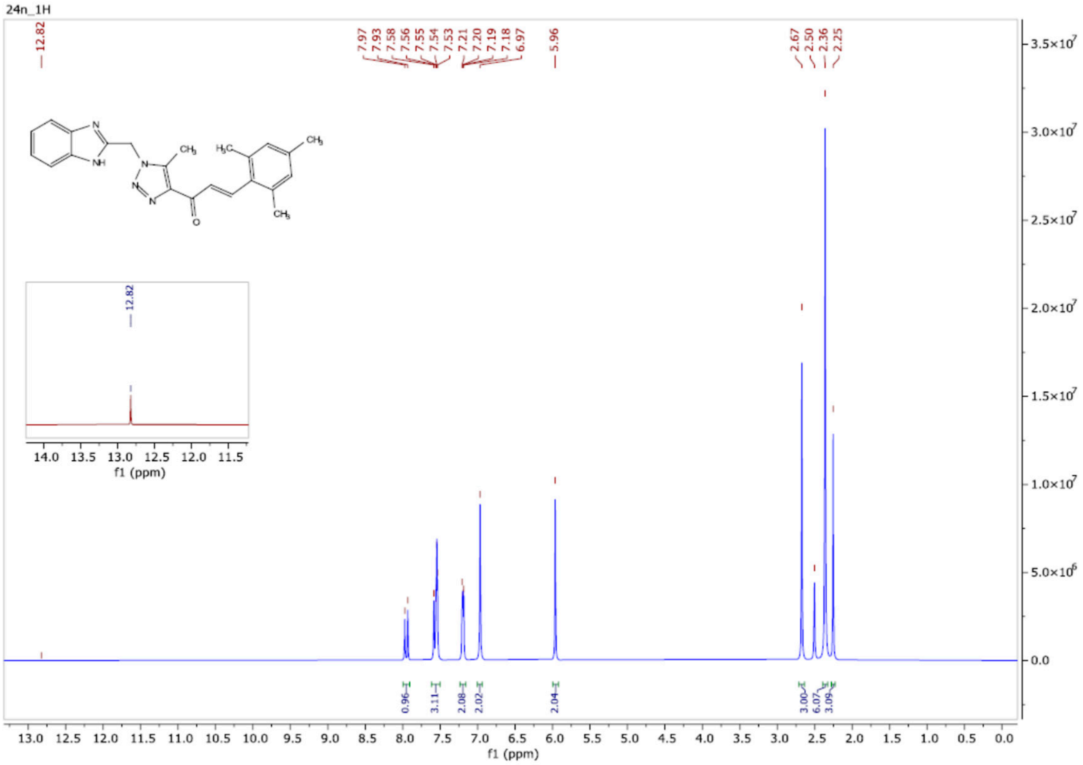

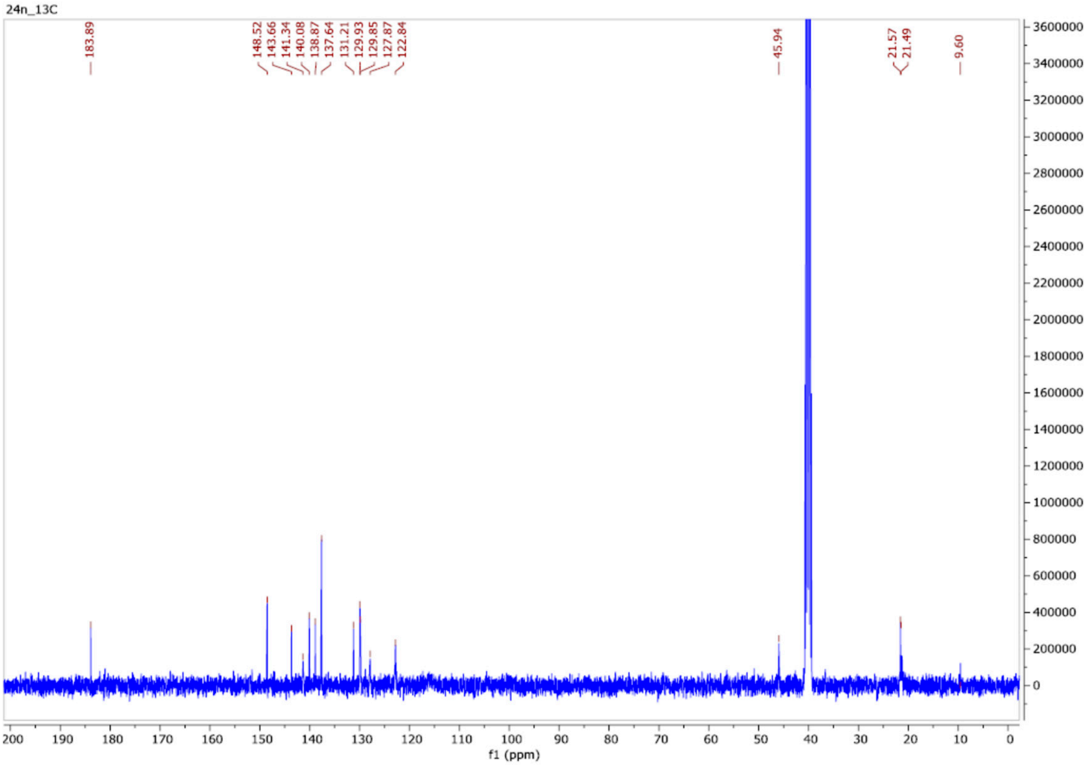

Figure S16. ^{1}H-NMR &^{13}C-NMR Spectrum of compound (**5n**) (DMSO-d_6).

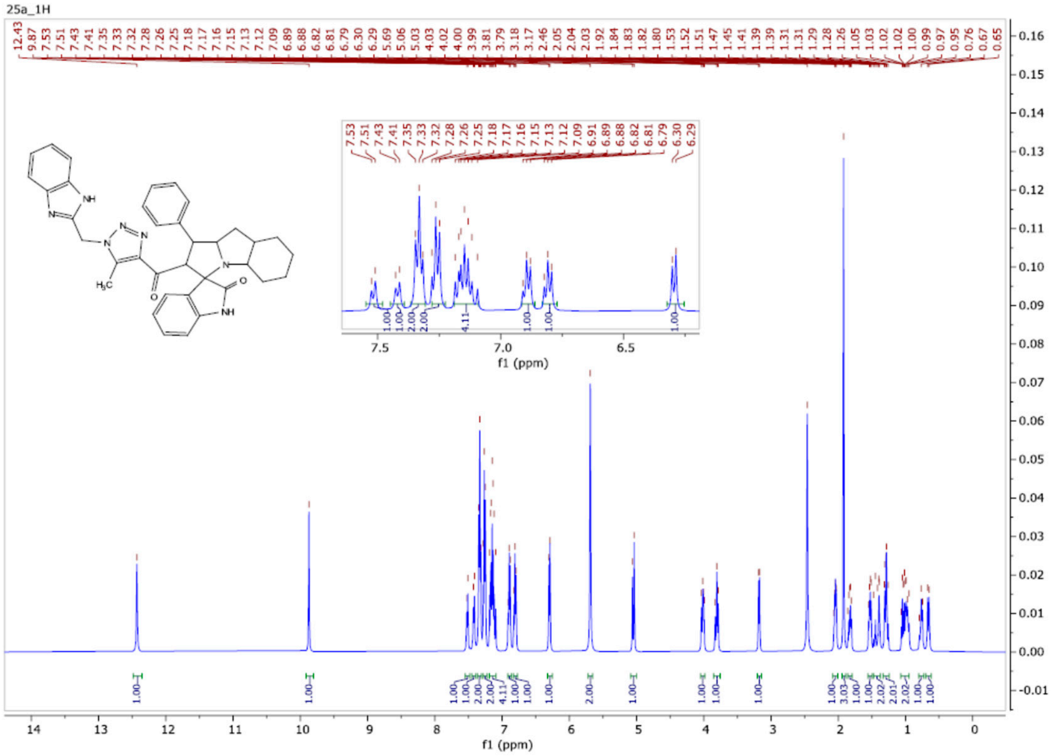

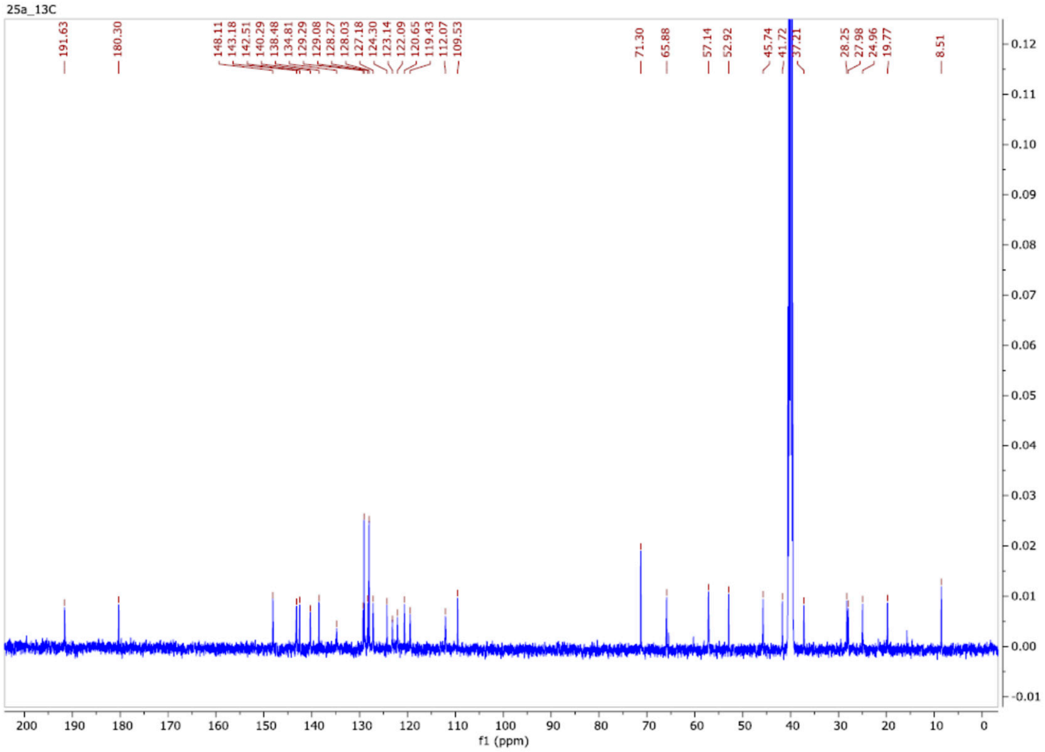

Figure S17. ^{1}H-NMR &^{13}C-NMR Spectrum of compound (**8a**) (DMSO-d_6).

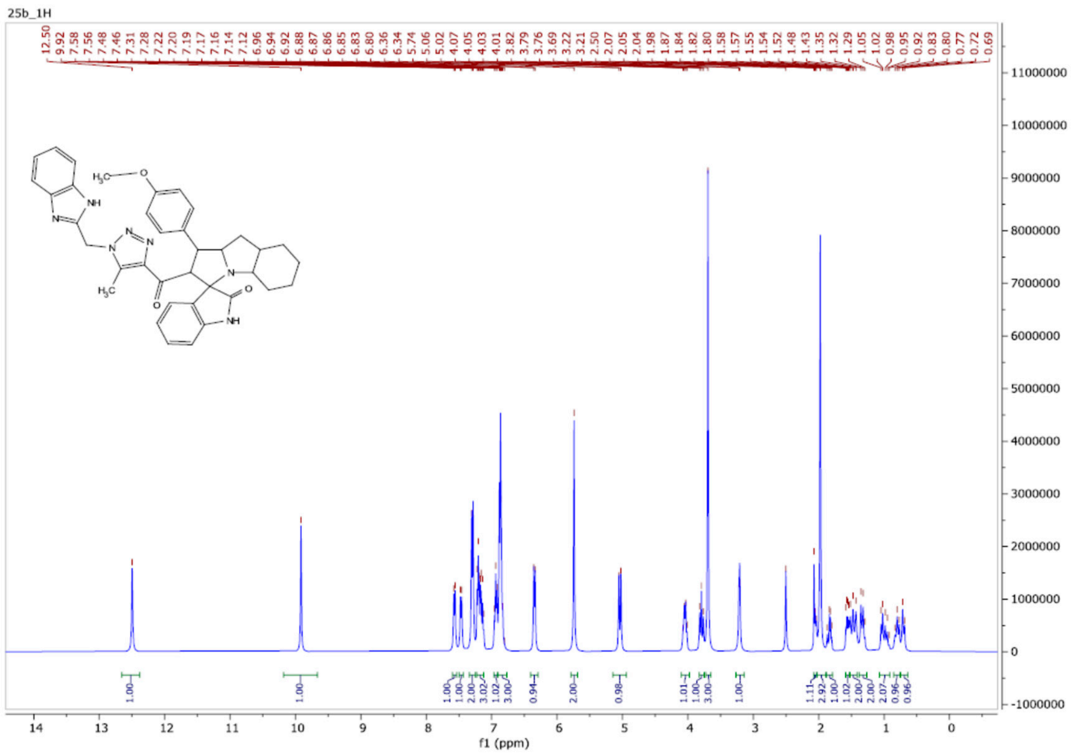

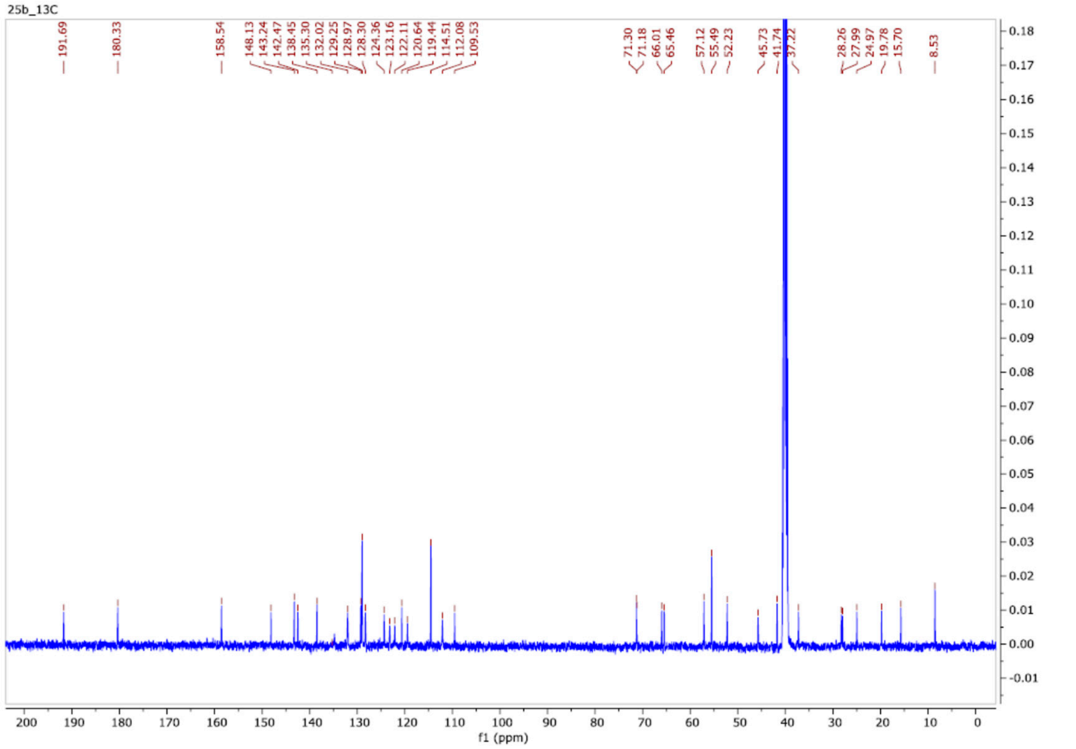

Figure S18. ^{1}H-NMR &^{13}C-NMR Spectrum of compound (**8b**) (DMSO-d_6).

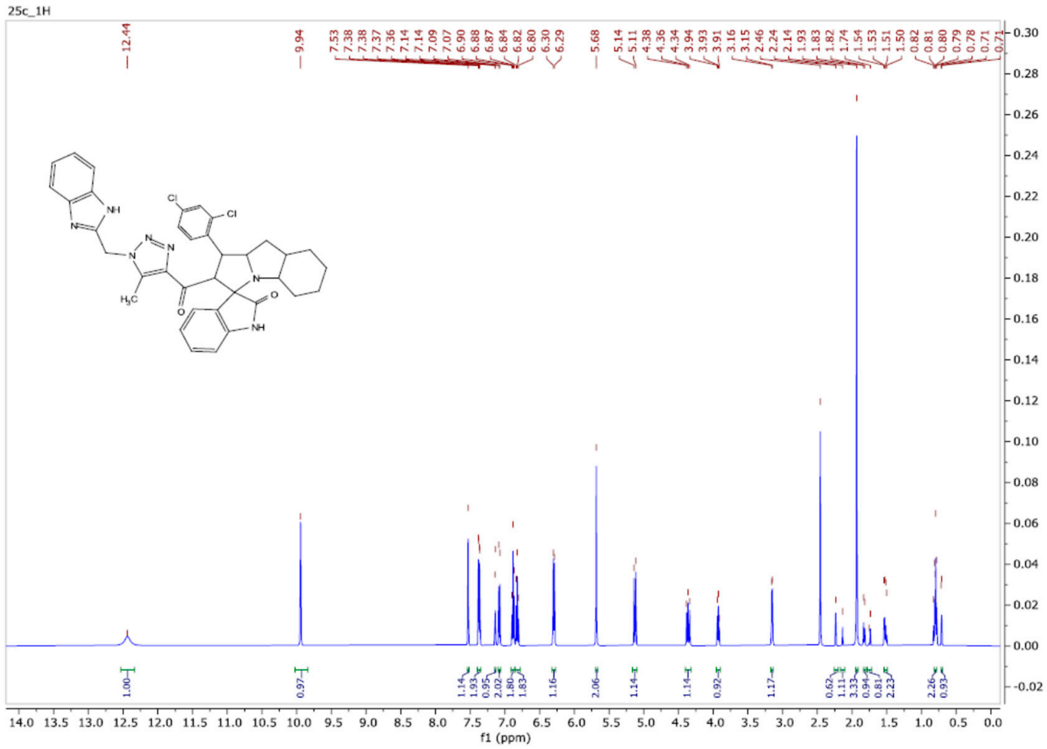

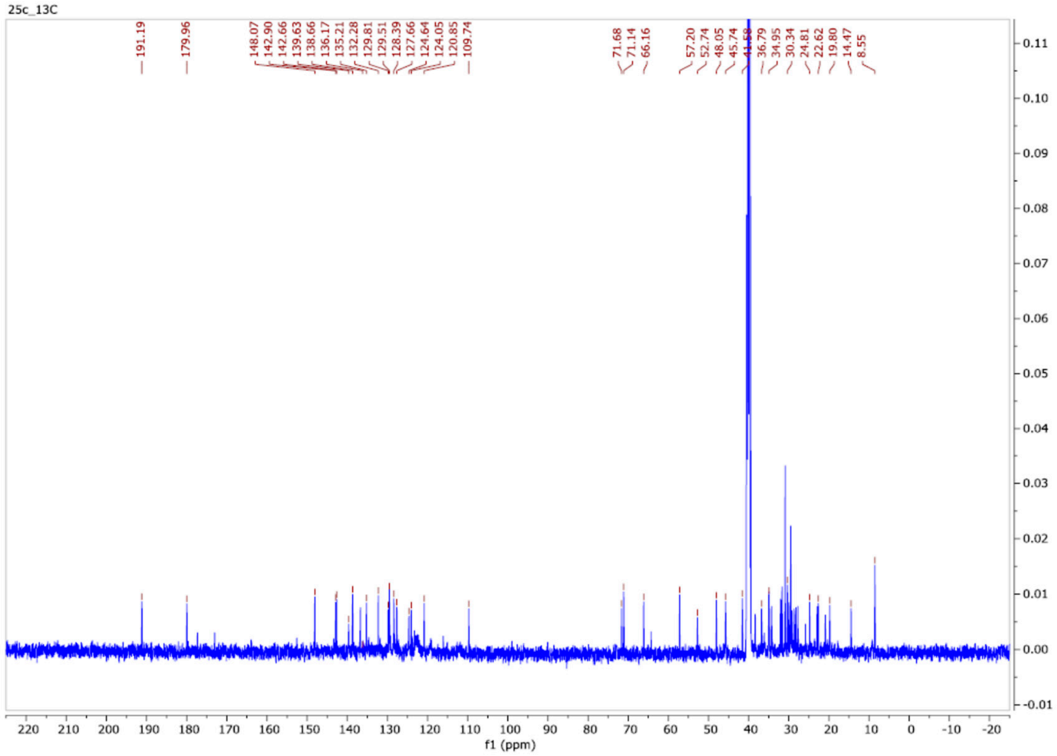

Figure S19. ^{1}H-NMR &^{13}C-NMR Spectrum of compound (**8c**) (DMSO-*d6*)

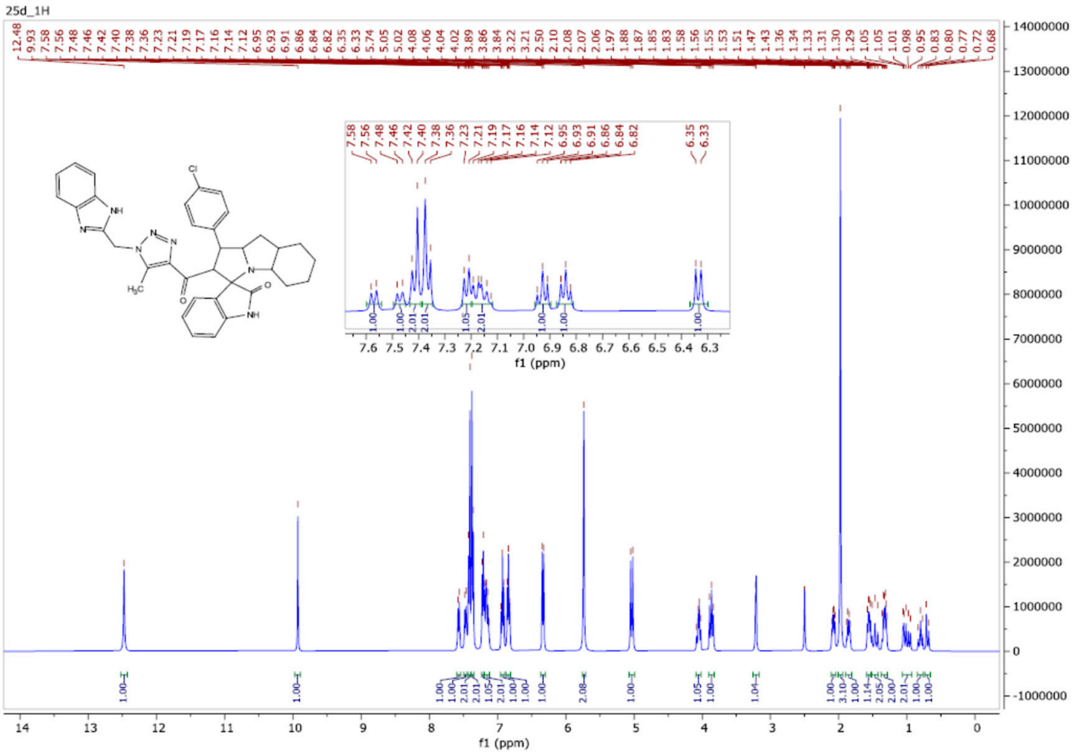

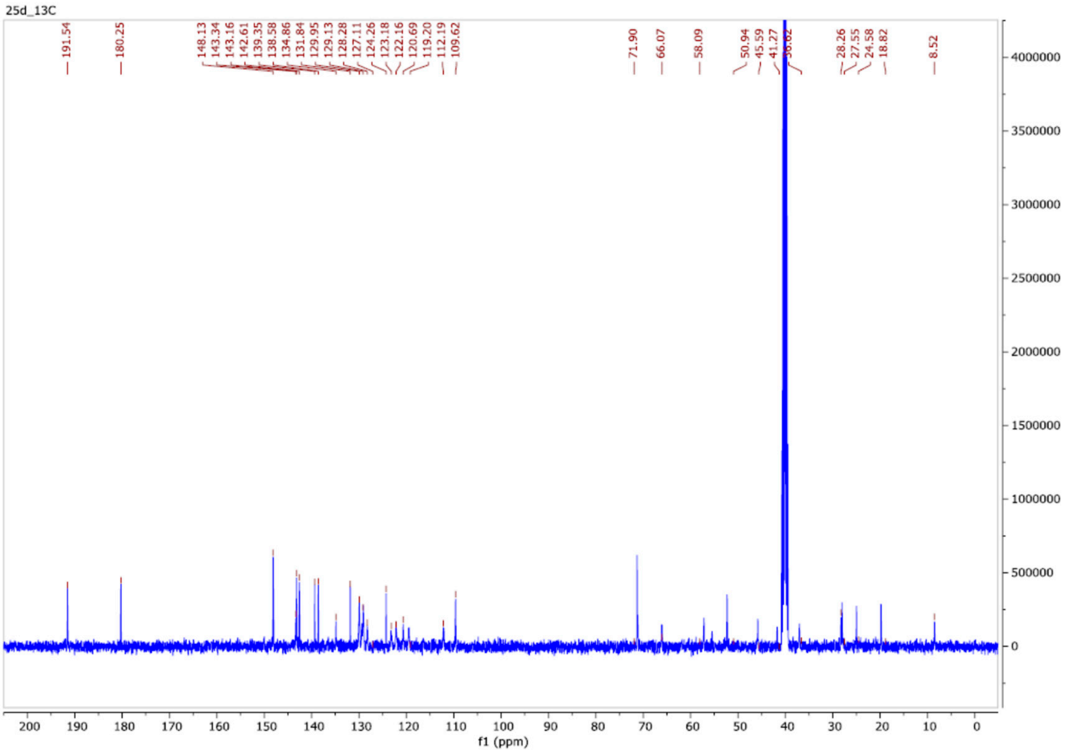

Figure S20. ^{1}H-NMR &^{13}C-NMR Spectrum of compound (**8d**) (DMSO-d_6).

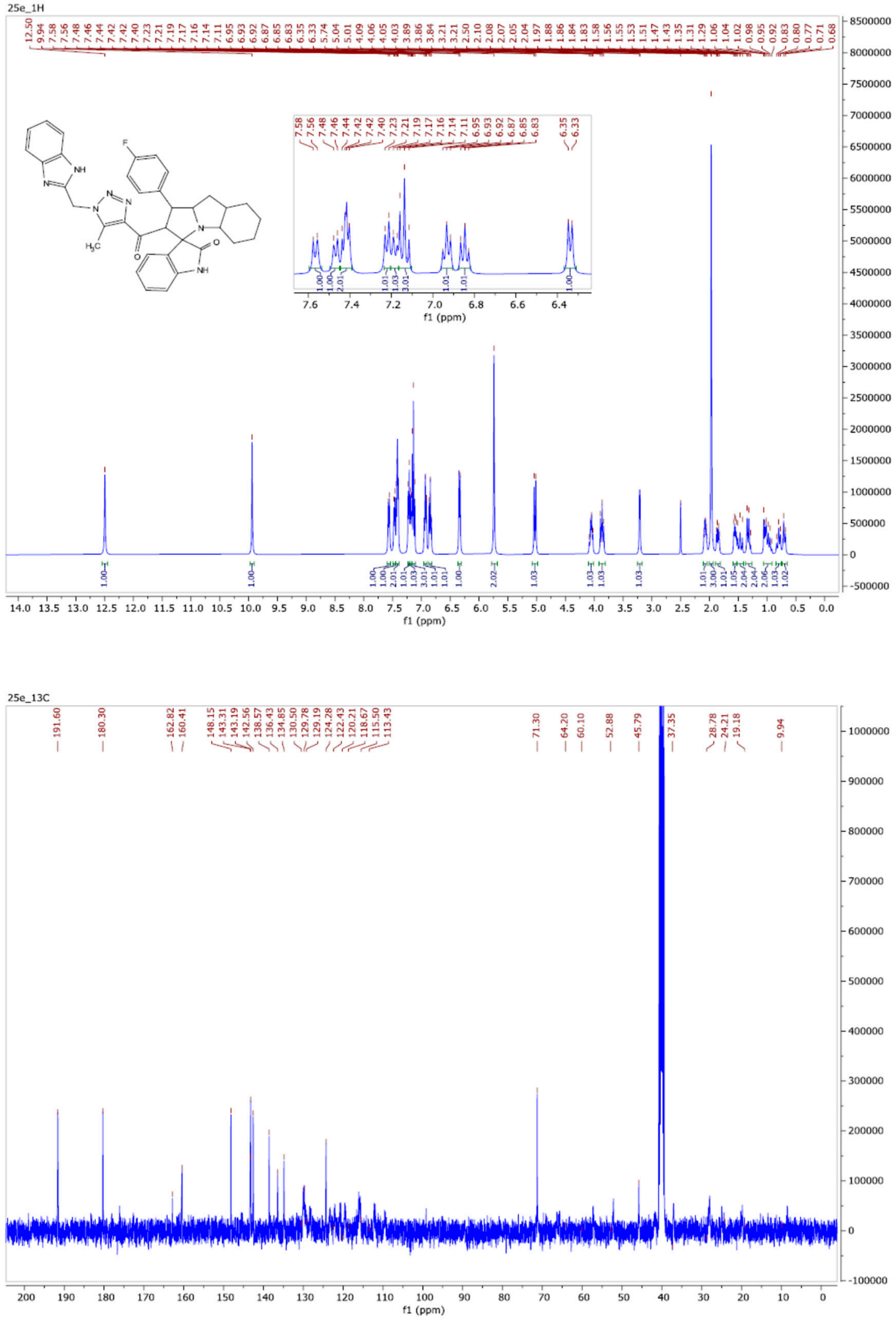

Figure S21. ^{1}H-NMR &^{13}C-NMR Spectrum of compound (**8e**) (DMSO-d_6).

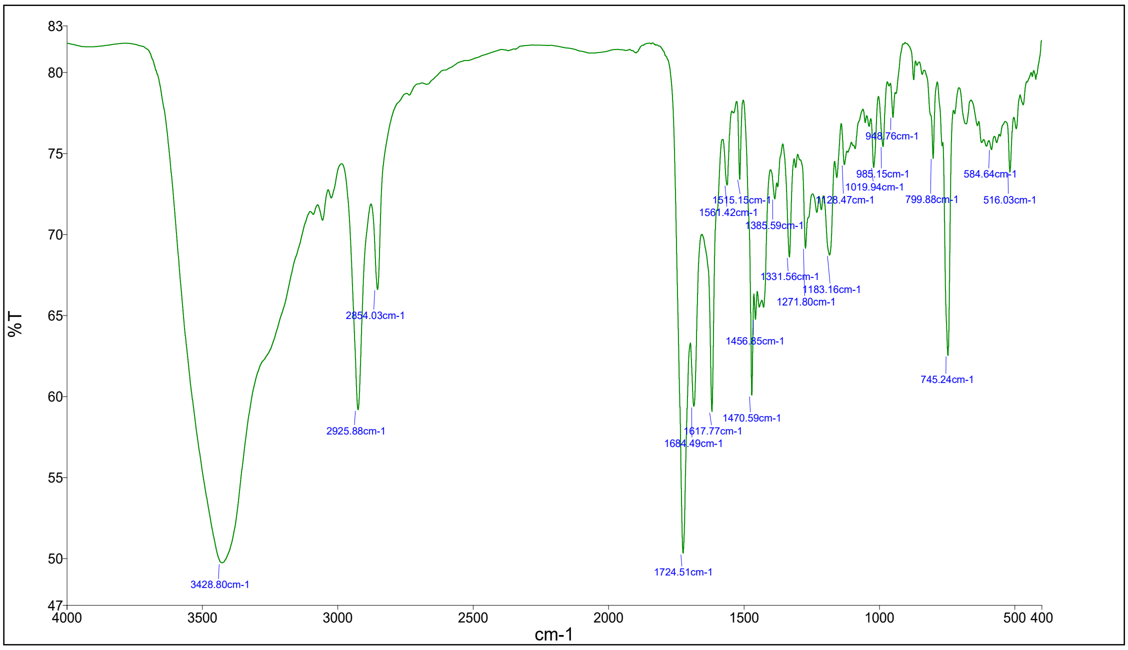

Figure S22. IR Spectrum of compound (**8f**).

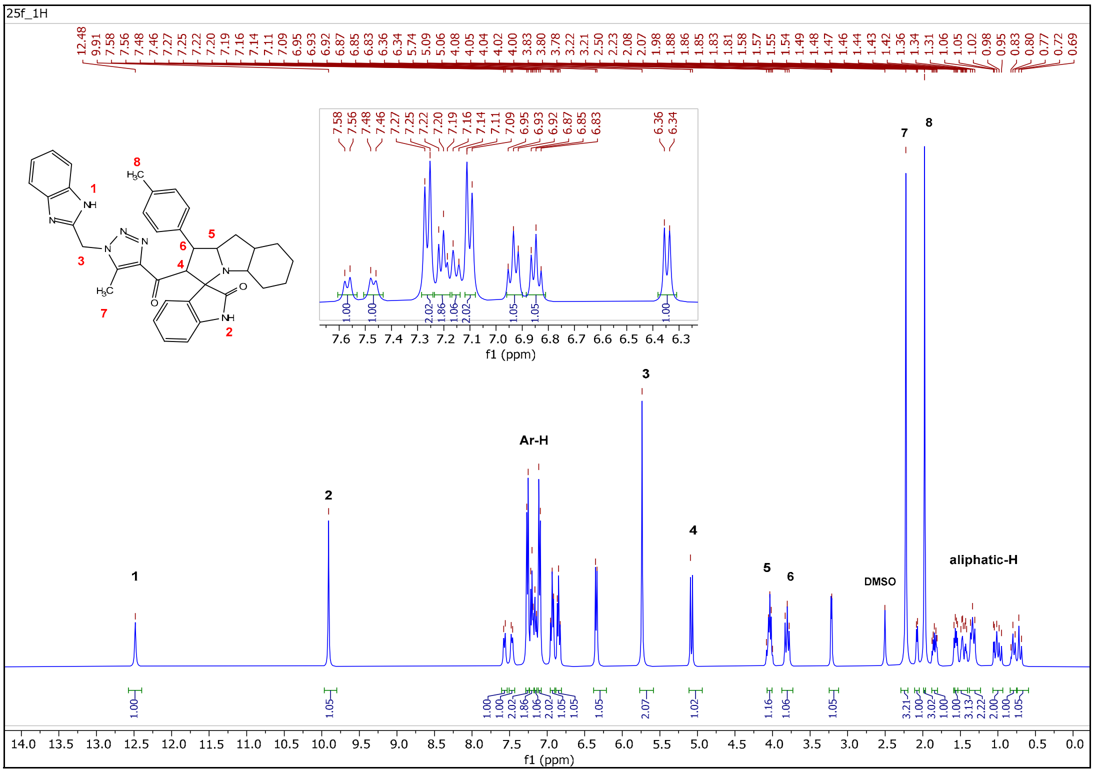

Figure S23. ^{1}H-NMR Spectrum of compound (**8f**) (DMSO-d_6)

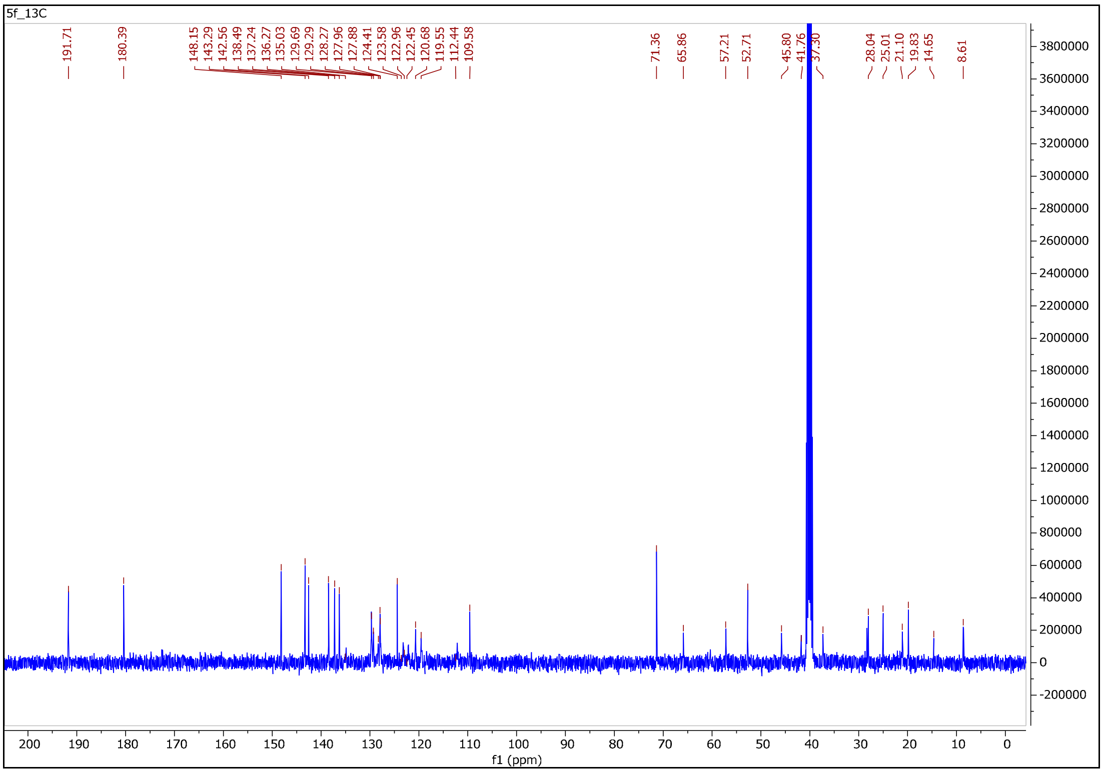

Figure S24. ^{13}C-NMR Spectrum of compound (**8f**) (DMSO-d_6).

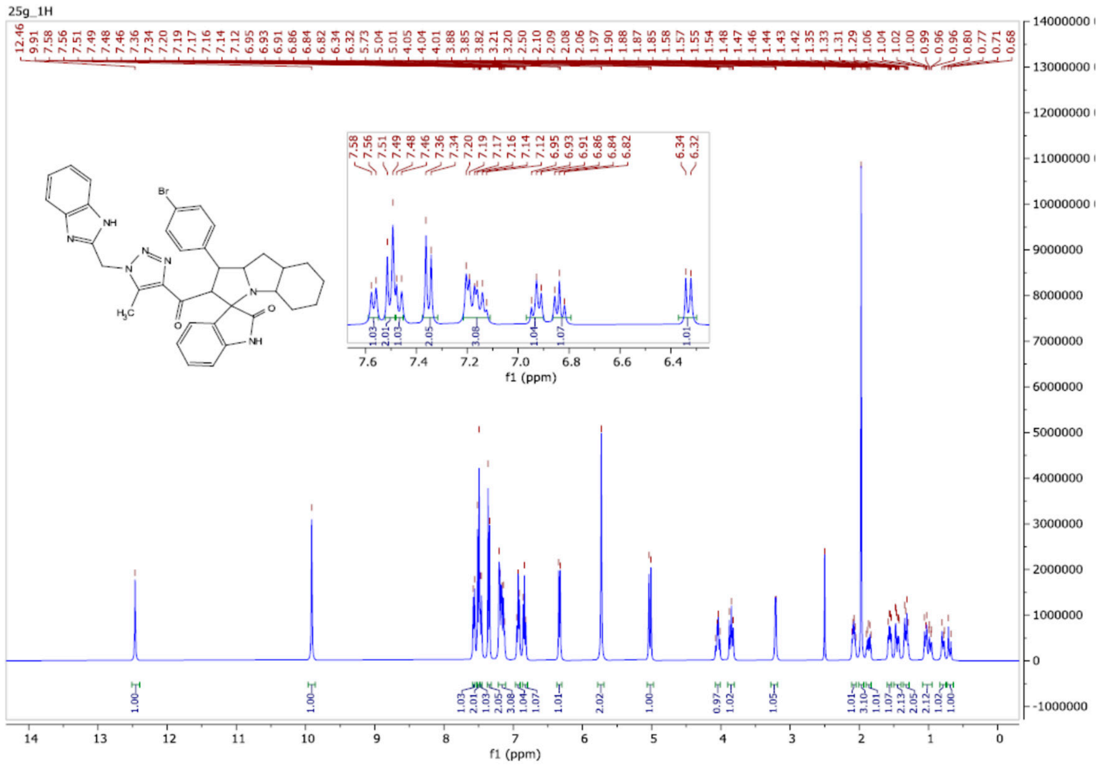

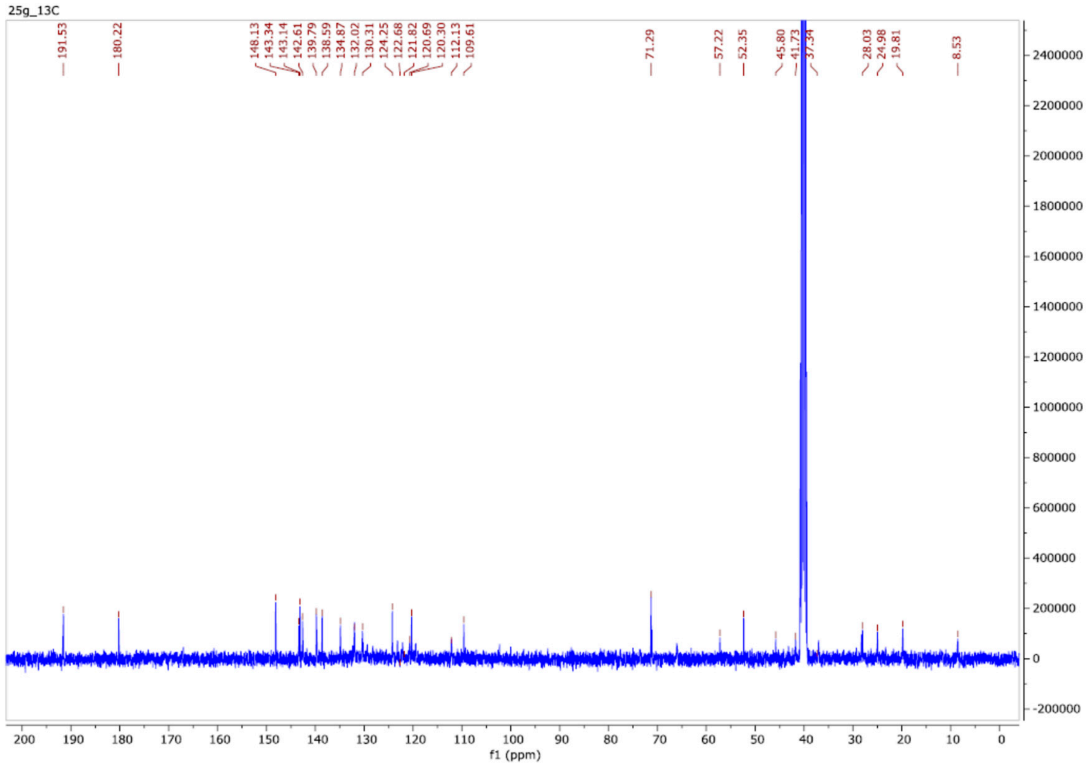

Figure S25. ^{1}H-NMR &^{13}C-NMR Spectrum of compound (**8g**) (DMSO-d_6).

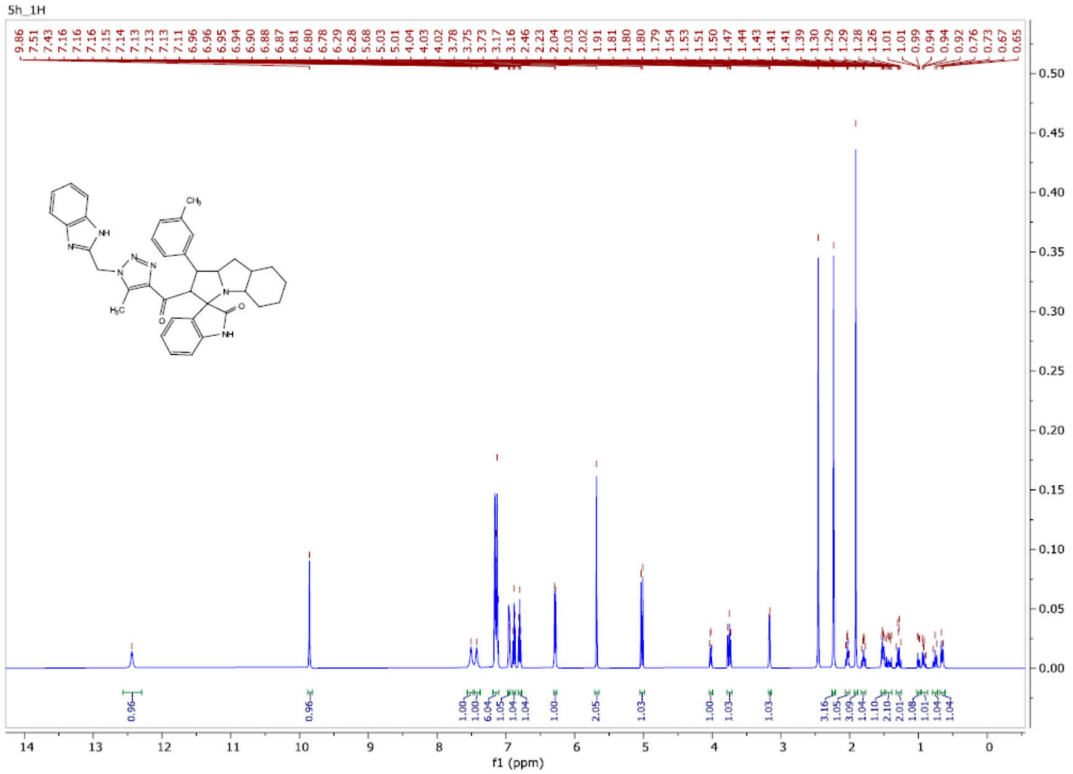

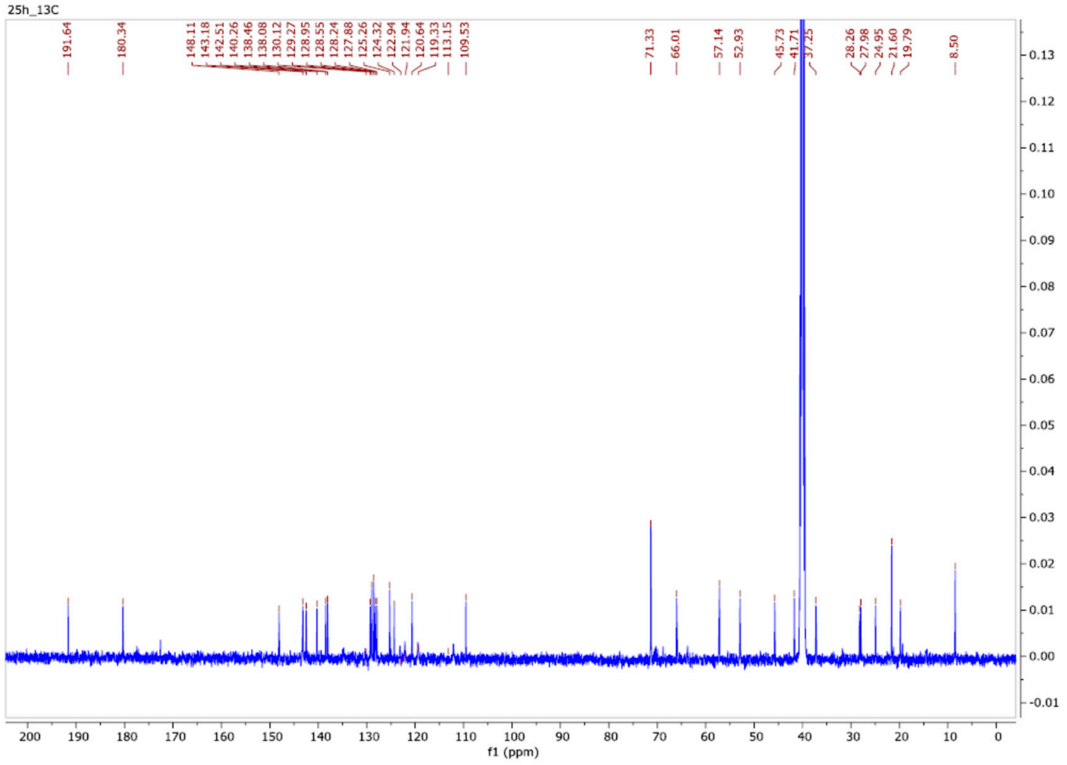

Figure S26. ^{1}H-NMR &^{13}C-NMR Spectrum of compound (**8h**) (DMSO-*d*$_6$).

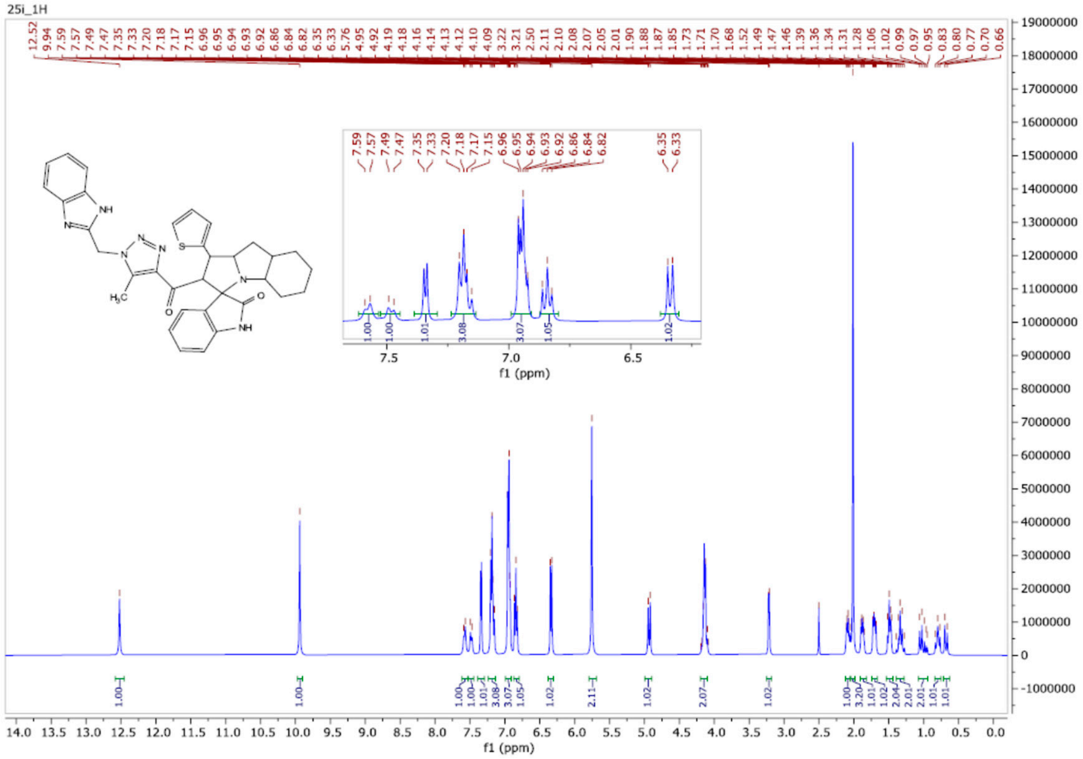

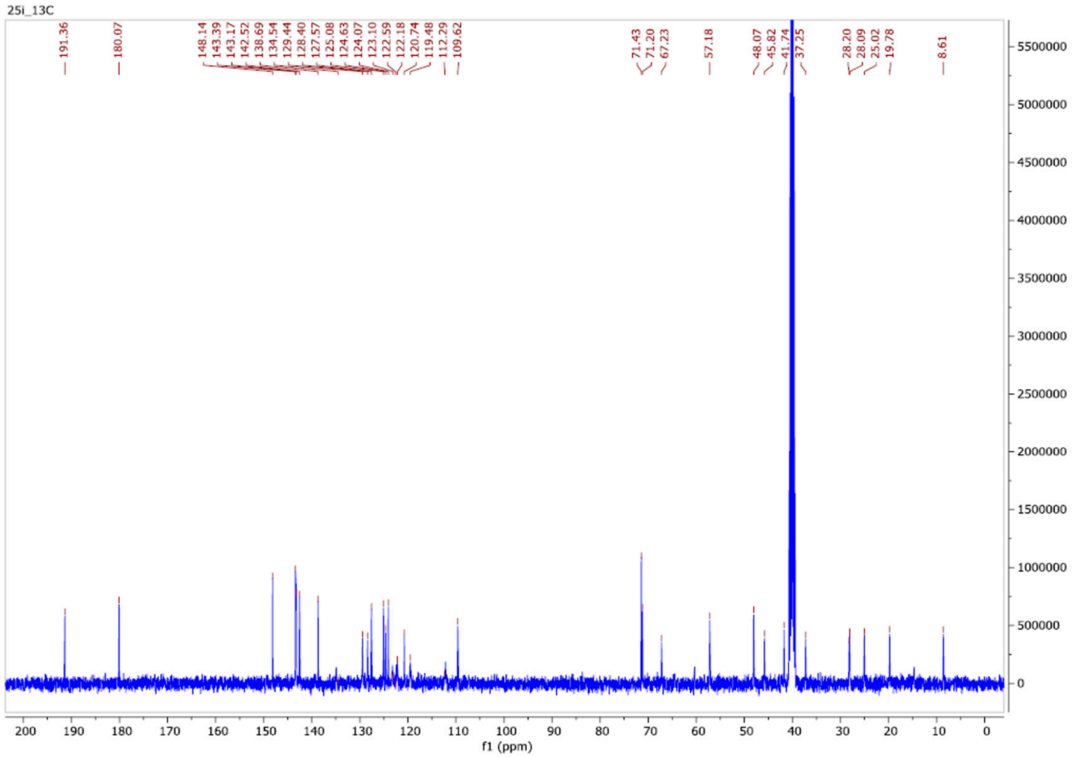

Figure S27. ^{1}H-NMR &^{13}C-NMR Spectrum of compound (**8i**) (DMSO-d_6).

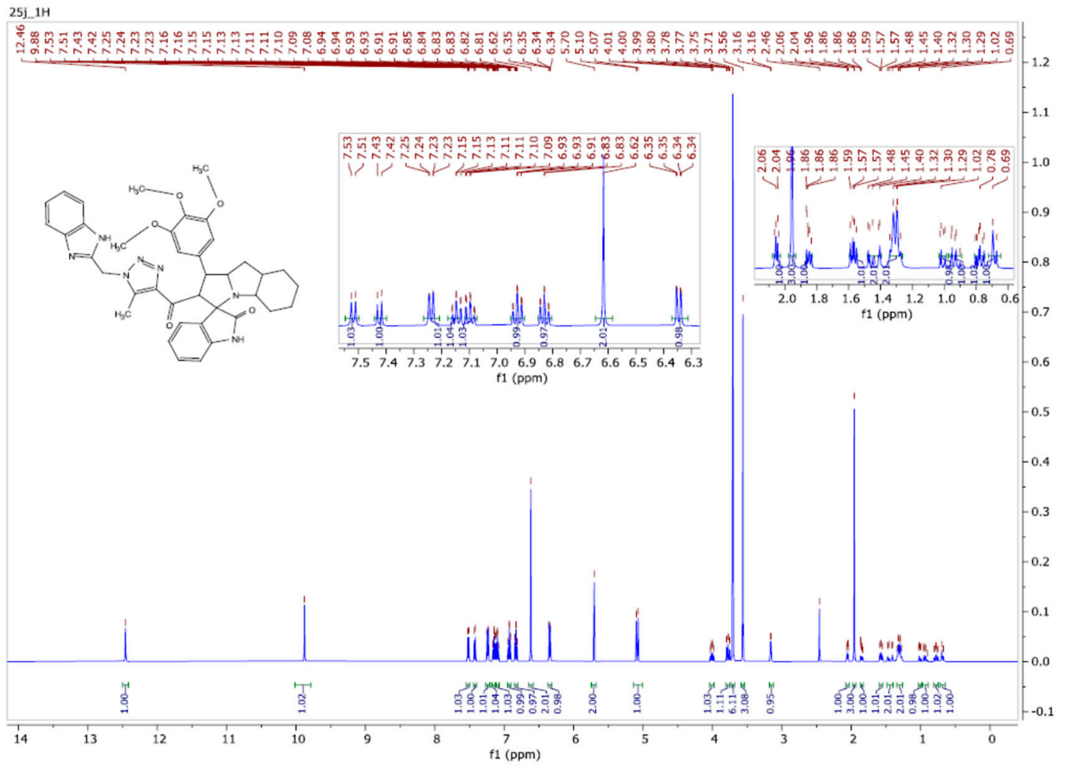

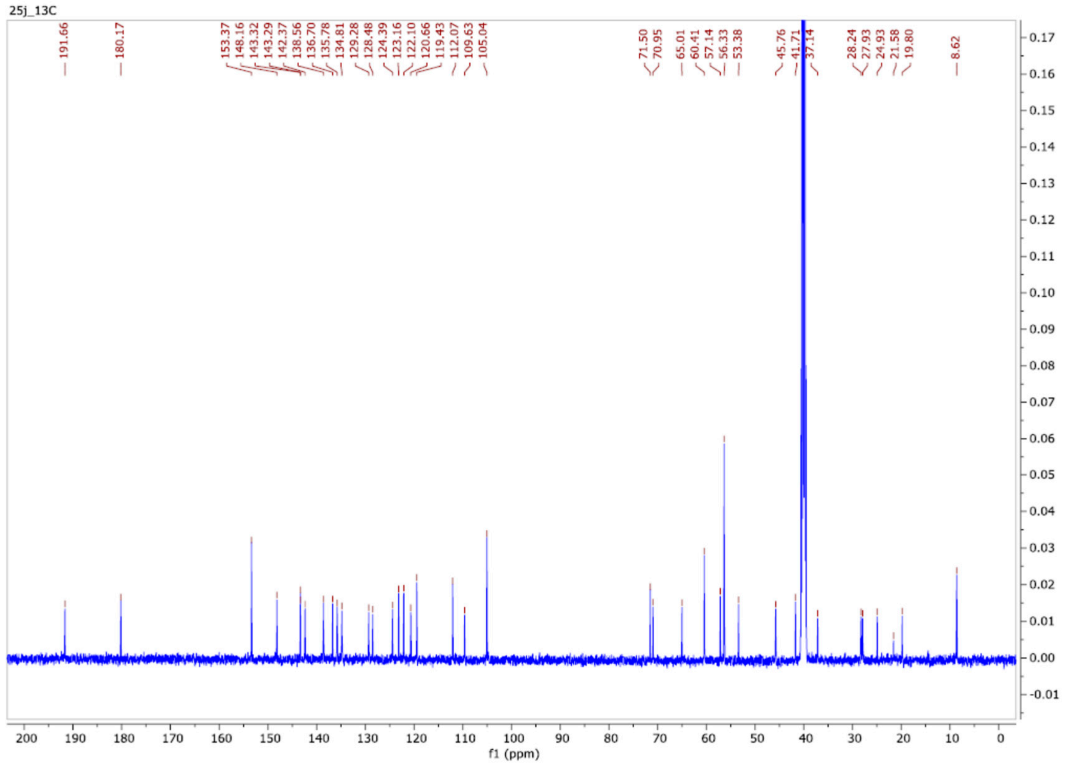

Figure S28. ^{1}H-NMR &^{13}C-NMR Spectrum of compound (**8j**) (DMSO-d_6).

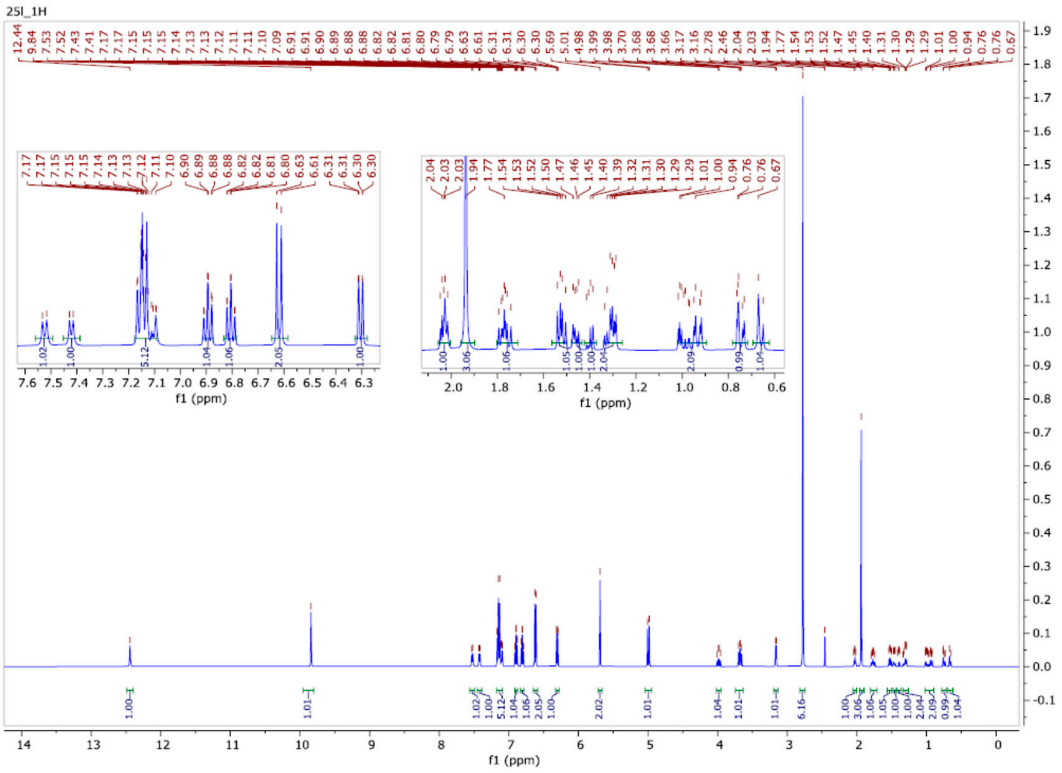

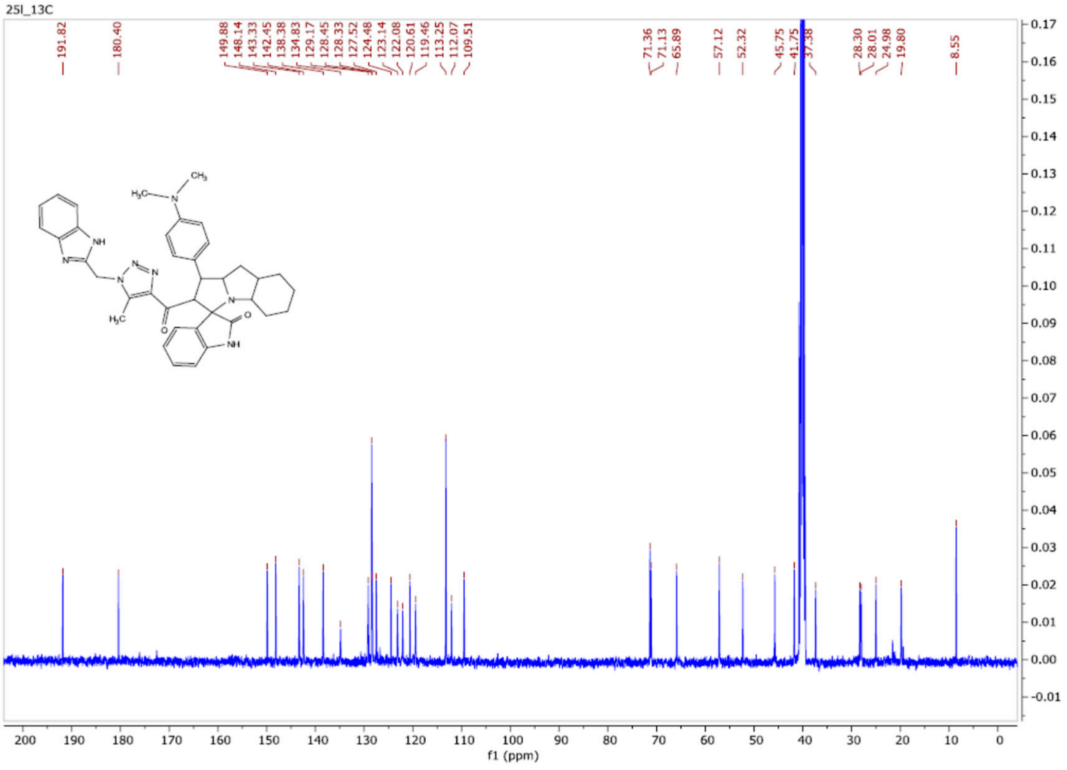

Figure S29. ^{1}H-NMR &^{13}C-NMR Spectrum of compound (**8l**) (DMSO-d_6).

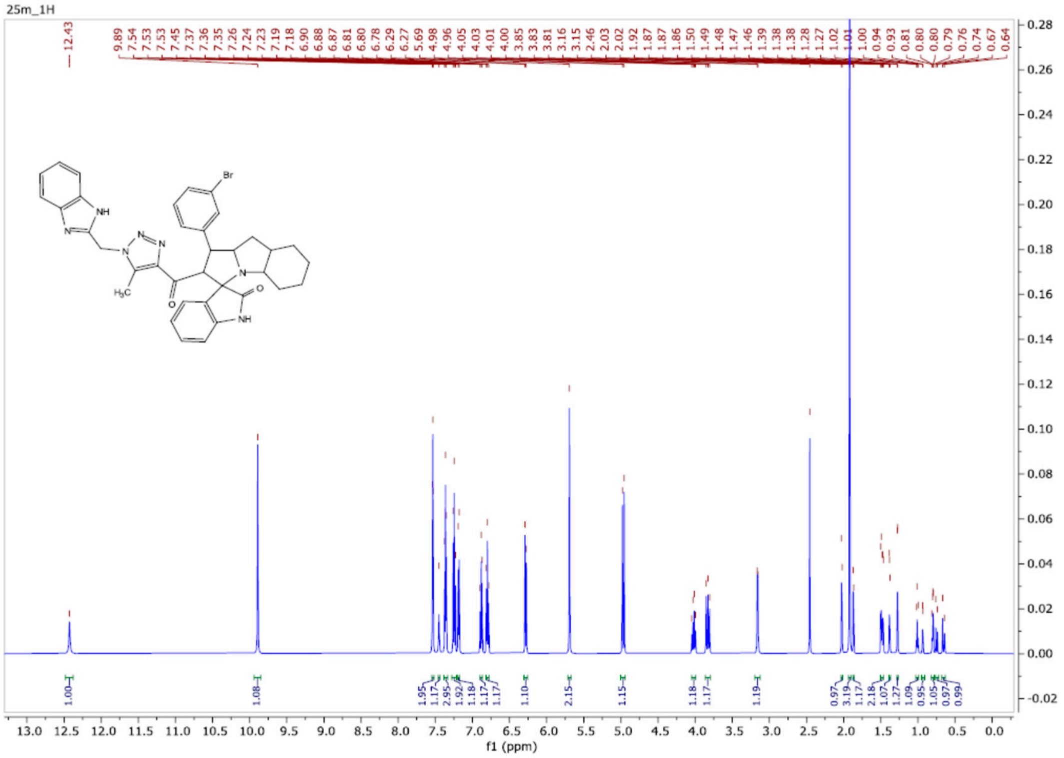

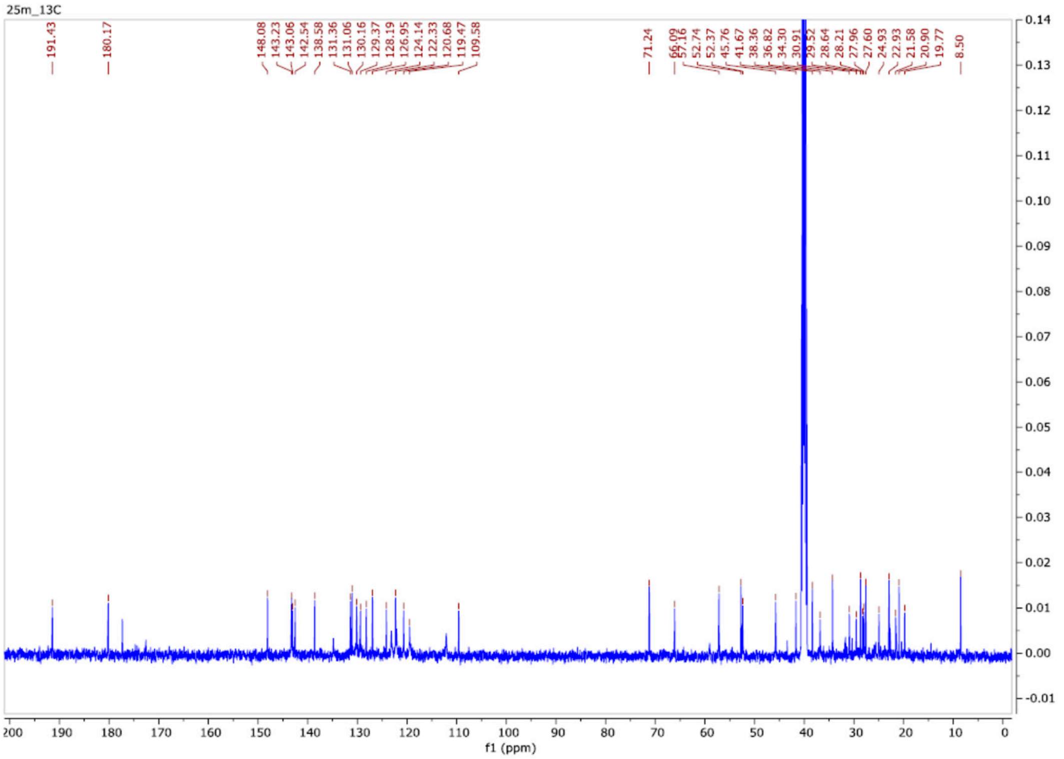

Figure S30. ^{1}H-NMR &^{13}C-NMR Spectrum of compound (**8m**) (DMSO-*d*$_6$).

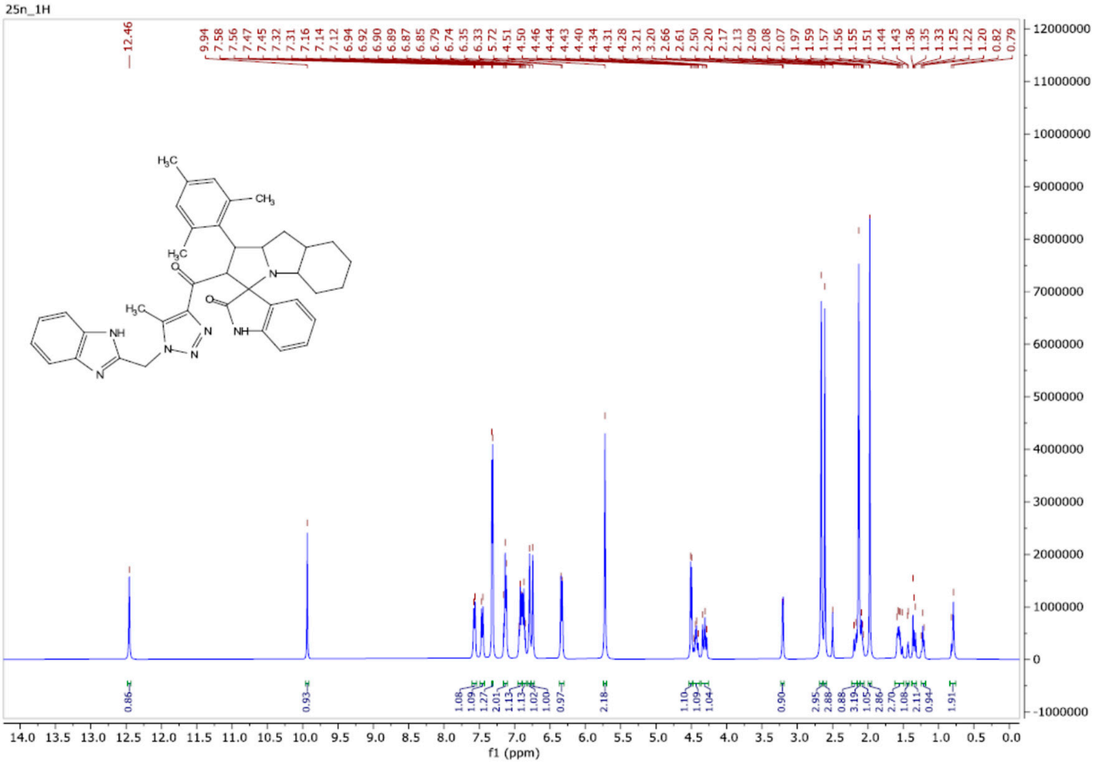

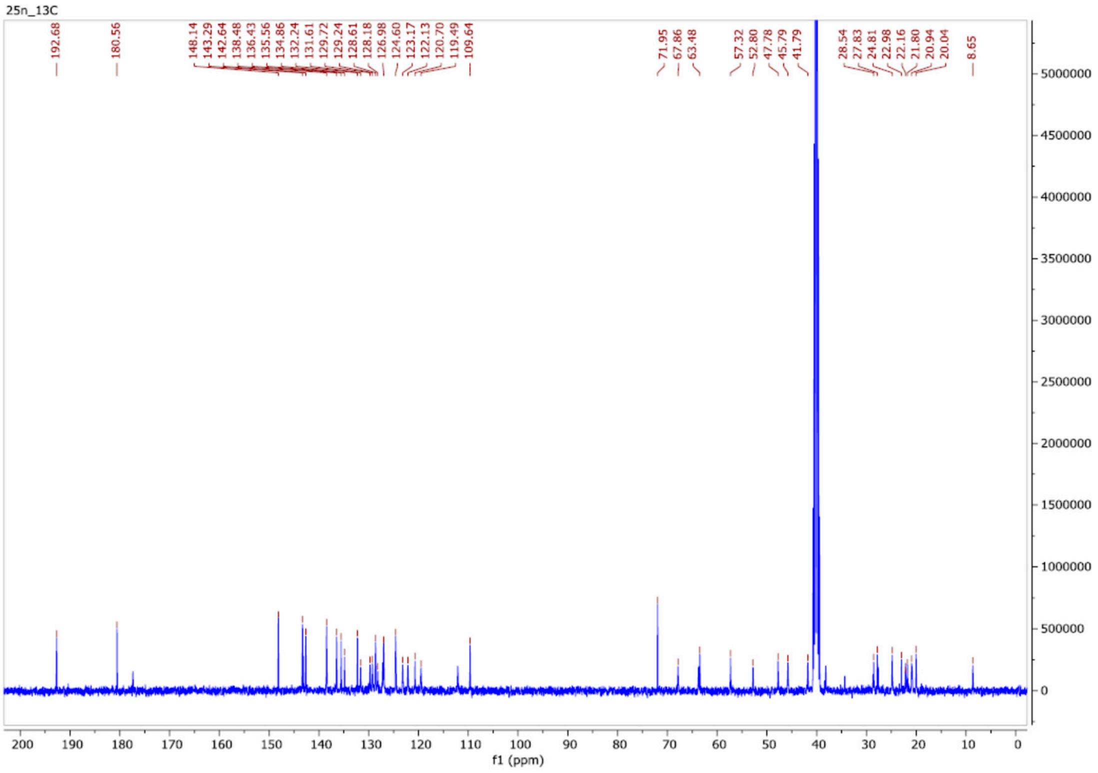

Figure S31. ^{1}H-NMR &^{13}C-NMR Spectrum of compound (**8n**). (DMSO-d_6).

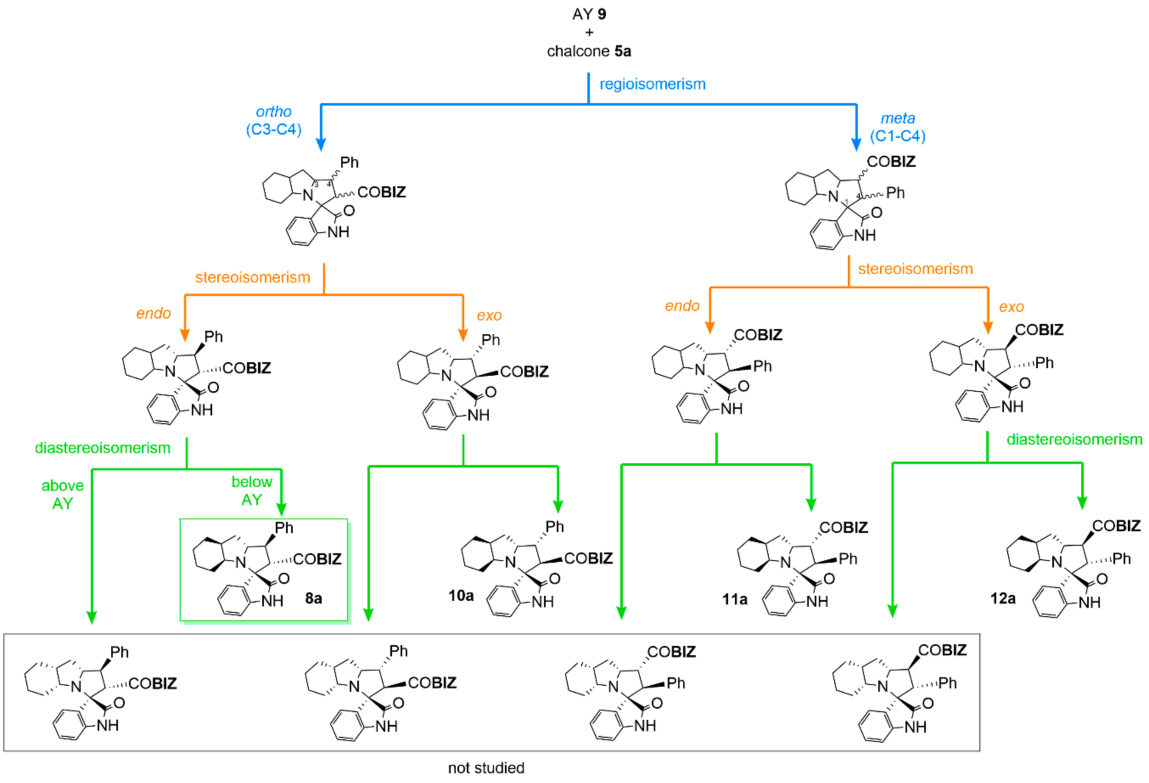

Figure S32. Reaction mechanism roadmap of the competitive isomeric reaction paths in the 32CA reaction of AY **9** with chalcone **5a.**

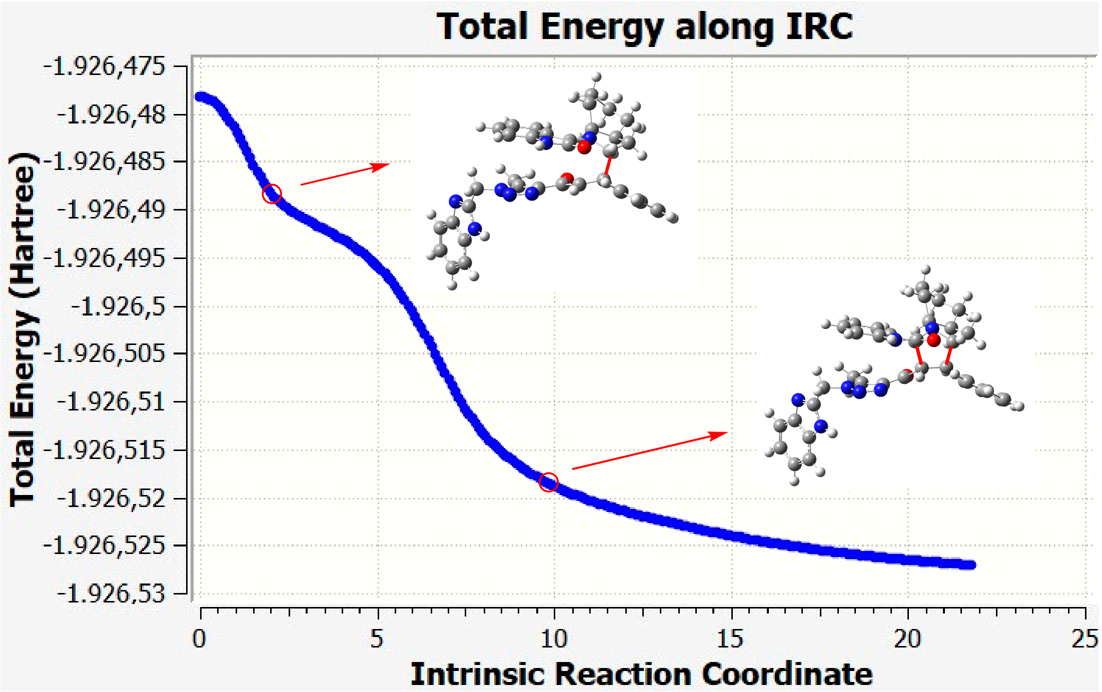

Figure S33. *ω*B97X-D/6-311G(d,p) IRC path associated with the most favourable *ortho/endo* reaction path via **TS-on** in methanol.

Table S1. *ω*B97X-D/6-311G(d,p) enthalpies (H, in kcal·mol^{-1}), entropies (S, in cal·mol^{-1}·K^{-1}) and Gibbs free energies (G, in kcal·mol^{-1}), and the relative ones with respect to the separated reagents (ΔH, ΔS and ΔG), computed at 61 ºC and 1 atm in methanol, for the stationary points involved in the 32CA reaction of AY **9** with chalcone **5a**.

	H	ΔH	S	ΔS	G	ΔG
AY **9**	-804.886382		130.365		-804.956570	
Chalcone **5a**	-1120.866046		176.928		-1120.961303	
**MC-on**	-1925.782775	-19.0	254.432	-52.9	-1925.919761	-1.2
**TS-on**	-1925.769785	-10.9	245.595	-61.7	-1925.902013	10.0
**TS-ox**	-1925.760629	-5.1	249.646	-57.6	-1925.895038	14.3
**TS-mn**	-1925.763649	-7.0	249.944	-57.3	-1925.898219	12.3
**TS-mx**	-1925.757641	-3.3	251.838	-55.5	-1925.893230	15.5
**8a**	-1925.825316	-45.7	243.008	-64.3	-1925.956152	-24.0
**10a**	-1925.817515	-40.8	239.965	-67.3	-1925.946711	-18.1
**11a**	-1925.814995	-39.3	252.773	-54.5	-1925.951088	-20.8
**12a**	-1925.813804	-38.5	255.927	-51.4	-1925.951595	-21.2

Cartesian coordinates and electronic energies of the stationary points involved in the 32CA reaction between AY **9** and chalcone **5a** in methanol. Imaginary frequencies for TSs at 60 ºC are included.

AY **9**
E(RwB97XD) = -805.223607105 A.U.

6	-1.247455	2.121596	-0.200818
7	-0.574943	1.008910	-0.203618
6	0.796334	0.863049	-0.062574
6	1.611150	-0.322147	-0.163670
6	2.944412	0.115098	0.065822
6	4.028202	-0.746218	0.050452
6	3.796556	-2.096528	-0.201297
6	2.500927	-2.554123	-0.430996
6	1.412525	-1.685278	-0.414475
6	1.638631	1.988810	0.211997
1	5.030054	-0.372548	0.231032
1	0.426324	-2.090857	-0.598271
6	-1.472109	-0.173076	-0.302175
1	-0.715242	3.054946	-0.087791
7	2.927697	1.477254	0.288300
1	-1.075328	-0.822020	-1.082469
8	1.358879	3.187772	0.364308
1	3.733156	2.054140	0.462563
1	4.629079	-2.790394	-0.217946
6	-2.830682	0.432507	-0.718201
6	-2.713409	1.913250	-0.319003
1	-2.905078	0.369099	-1.806851
1	-3.197508	2.119267	0.644104
1	-3.158787	2.593629	-1.047771
6	-4.008327	-0.325816	-0.095051
6	-3.703249	-1.823539	-0.051185
6	-2.506799	-2.120344	0.878598
6	-1.566853	-0.912537	1.027423
1	-4.581435	-2.384754	0.274905
1	-3.478262	-2.162807	-1.068591
1	-2.864046	-2.401703	1.872232
1	-1.944964	-2.976474	0.494095
1	-1.955334	-0.215990	1.777609
1	-0.581999	-1.231786	1.371151
1	-4.916135	-0.127787	-0.669417
1	-4.193918	0.039444	0.921406
1	2.329988	-3.606953	-0.626318

Chalcone **5a**
E(RwB97XD) = -1121.23564268 A.U.

6	4.098525	0.259705	0.776600
6	2.975070	0.387651	0.060150
1	4.093440	0.683241	1.778790
1	2.872957	-0.000241	-0.945612
6	1.803250	1.080370	0.635994
8	1.802131	1.563049	1.758599
6	5.344070	-0.391897	0.365408
6	6.403235	-0.445630	1.278104
6	5.521371	-0.963440	-0.901778
6	7.605527	-1.053548	0.940173
1	6.278266	-0.005631	2.262015
6	6.721151	-1.569304	-1.239137
1	4.719330	-0.934114	-1.630177
6	7.766950	-1.616937	-0.319733
1	8.415044	-1.086626	1.659966
1	6.844443	-2.006779	-2.223056
1	8.703674	-2.091897	-0.587725
6	-4.507933	-1.196217	-0.411485
6	-5.069295	-0.229409	0.441041
6	-6.061633	-0.595400	1.353876
6	-6.458277	-1.920654	1.381315
6	-5.885738	-2.874753	0.520436
6	-4.901638	-2.532581	-0.391168
6	-3.623700	0.791953	-0.748394
1	-6.499741	0.142540	2.015388
1	-7.225780	-2.234983	2.078985
1	-6.224123	-3.903107	0.572557
1	-4.460976	-3.266595	-1.054473
7	-4.487685	1.007719	0.206680
7	-3.584753	-0.506079	-1.164657
1	-2.978545	-0.881681	-1.876962
6	-0.625424	1.734407	0.063237
6	0.605668	1.164155	-0.220546
7	-0.600071	0.862496	-1.988635
7	0.561534	0.647672	-1.481280
7	-1.326574	1.527211	-1.067282
6	-1.177851	2.432920	1.252670
1	-2.157197	2.028512	1.518544
1	-0.491563	2.318221	2.087406
1	-1.297403	3.499561	1.042881
6	-2.723642	1.833633	-1.333842
1	-2.831596	1.891050	-2.417007
1	-2.972565	2.800849	-0.901603

MC-on
E(RwB97XD) = -1926.49229855 A.U.

6	-3.982598	-0.444481	0.113204
7	-2.893358	-1.142271	0.244717
6	-1.982951	-1.071747	1.284130
6	-0.747140	-1.790177	1.471185
6	-0.220538	-1.321602	2.705104
6	0.980898	-1.768059	3.225488
6	1.696174	-2.724030	2.507640
6	1.206568	-3.199846	1.293917
6	0.001014	-2.740695	0.769232
6	-2.187166	-0.196452	2.403489
1	1.350878	-1.379078	4.167361
1	-0.327618	-3.132254	-0.183697
6	-2.689379	-2.059098	-0.905671
1	-4.271148	0.236410	0.900357
7	-1.096118	-0.397015	3.234654
1	-1.653969	-1.951455	-1.225645
8	-3.109674	0.595427	2.651301
1	-0.938894	0.149410	4.064302
1	2.634792	-3.099328	2.899594
6	-3.658464	-1.512146	-1.974631
6	-4.699858	-0.730744	-1.156025
1	-3.096037	-0.806417	-2.591569
1	-5.599068	-1.326419	-0.954461
1	-5.032312	0.190802	-1.640041
6	-4.217164	-2.628892	-2.864348
6	-3.151847	-3.703522	-3.087877
6	-2.782364	-4.403382	-1.762410
6	-3.022600	-3.500707	-0.540416
1	-3.495318	-4.439234	-3.818232
1	-2.263296	-3.228892	-3.519223
1	-3.374401	-5.313951	-1.641260
1	-1.733603	-4.713229	-1.790657
1	-4.073810	-3.542471	-0.236539
1	-2.434518	-3.839968	0.313718
1	-4.554127	-2.208498	-3.814576
1	-5.096617	-3.081429	-2.392218
6	-2.600414	2.003018	-0.946096
6	-1.540777	1.803934	-0.152920
1	-2.574803	1.544412	-1.932662
1	-1.456510	2.252901	0.828325
6	-0.438719	0.930551	-0.598705
8	-0.488107	0.280751	-1.636790
6	-3.789523	2.799787	-0.632222

6	-4.605807	3.240828	-1.679056
6	-4.150170	3.111628	0.684944
6	-5.738663	4.002871	-1.422732
1	-4.343054	2.988064	-2.701249
6	-5.284170	3.868960	0.938304
1	-3.568005	2.712531	1.508423
6	-6.077841	4.322086	-0.112924
1	-6.358095	4.343183	-2.244488
1	-5.559295	4.097821	1.961619
1	-6.964709	4.911178	0.090861
1	1.773682	-3.938741	0.738858
6	6.238038	1.461266	-0.685476
6	6.603806	0.104771	-0.735572
6	7.652820	-0.304709	-1.562301
6	8.301461	0.661252	-2.311497
6	7.922196	2.014307	-2.248332
6	6.884857	2.440518	-1.436076
6	4.976622	0.231777	0.637072
1	7.941410	-1.348117	-1.608502
1	9.119294	0.373712	-2.961992
1	8.455419	2.740839	-2.850479
1	6.592378	3.482153	-1.387017
7	5.787681	-0.639907	0.102323
7	5.190894	1.508980	0.207969
1	4.678591	2.327712	0.494689
6	1.885839	0.099619	0.123883
6	0.745630	0.871662	0.270508
7	1.974782	1.315260	1.990234
7	0.855522	1.591459	1.421612
7	2.609929	0.411636	1.214621
6	2.345892	-0.836423	-0.932967
1	3.034139	-0.325729	-1.613204
1	1.487175	-1.182331	-1.502414
1	2.864826	-1.692131	-0.499255
6	3.902598	-0.110669	1.619490
1	4.108818	0.318054	2.600873
1	3.847178	-1.194646	1.715371

TS-on
E(RwB97XD) = -1926.47821736 A.U.
Imaginary frequency = -421.8293 cm^{-1}

6	-3.873030	-0.012278	-0.086256
7	-2.960475	-0.977905	0.150471
6	-2.057426	-0.909349	1.147727
6	-0.880544	-1.728587	1.378182

6	-0.373554	-1.333561	2.635640
6	0.802592	-1.839968	3.156981
6	1.490033	-2.791045	2.407245
6	1.006406	-3.206416	1.169743
6	-0.168225	-2.675469	0.643894
6	-2.285514	-0.107856	2.367413
1	1.173218	-1.504020	4.117776
1	-0.504380	-3.012008	-0.326301
6	-2.900066	-1.972141	-0.946975
1	-4.240809	0.531630	0.770534
7	-1.222406	-0.393508	3.192720
1	-1.880016	-1.998367	-1.323623
8	-3.217715	0.631530	2.664753
1	-1.086690	0.063724	4.078812
1	2.408789	-3.214428	2.796913
6	-3.850587	-1.393099	-2.018786
6	-4.739190	-0.413338	-1.240182
1	-3.239158	-0.827559	-2.727291
1	-5.647938	-0.903245	-0.871161
1	-5.053991	0.447467	-1.833054
6	-4.594923	-2.496590	-2.778246
6	-3.675057	-3.700093	-2.989679
6	-3.282062	-4.344981	-1.642679
6	-3.355613	-3.347568	-0.474824
1	-4.152222	-4.440074	-3.635918
1	-2.776010	-3.361029	-3.516575
1	-3.944174	-5.186005	-1.421868
1	-2.269822	-4.753751	-1.712731
1	-4.383551	-3.263665	-0.107100
1	-2.747843	-3.692198	0.364361
1	-4.953607	-2.106719	-3.733777
1	-5.481192	-2.808848	-2.214010
6	-2.676588	1.592277	-0.701963
6	-1.507824	1.503907	0.040679
1	-2.595235	1.218883	-1.720009
1	-1.363975	2.080671	0.944478
6	-0.393609	0.728254	-0.453685
8	-0.432951	0.071497	-1.500804
6	-3.650512	2.695741	-0.520318
6	-4.288540	3.235057	-1.639598
6	-3.960845	3.210869	0.742348
6	-5.198738	4.278192	-1.506787
1	-4.060250	2.840296	-2.624597
6	-4.867749	4.253232	0.874065
1	-3.509401	2.766603	1.623398
6	-5.488879	4.793314	-0.249299
1	-5.680217	4.687601	-2.387765

1	-5.098984	4.641579	1.859781
1	-6.199319	5.605097	-0.142446
1	1.554106	-3.947052	0.599297
6	6.315366	1.346424	-0.723921
6	6.707998	-0.003293	-0.746918
6	7.756528	-0.410477	-1.575333
6	8.377655	0.550828	-2.353379
6	7.971639	1.897042	-2.317131
6	6.934239	2.320893	-1.503648
6	5.092218	0.122520	0.638928
1	8.065783	-1.448673	-1.600939
1	9.194378	0.264795	-3.005942
1	8.483635	2.620038	-2.941546
1	6.621025	3.357263	-1.475314
7	5.915218	-0.744883	0.115916
7	5.276436	1.393636	0.179265
1	4.749177	2.207805	0.451566
6	1.991806	-0.006855	0.181247
6	0.838107	0.733115	0.365661
7	2.101384	1.182627	2.063286
7	0.961067	1.440274	1.523307
7	2.736148	0.305271	1.262040
6	2.451958	-0.920505	-0.896367
1	3.168607	-0.408666	-1.545761
1	1.593627	-1.222221	-1.490987
1	2.940783	-1.803698	-0.481489
6	4.035913	-0.216318	1.641791
1	4.263213	0.215482	2.617297
1	3.985863	-1.300773	1.741931

TS-ox
E(RwB97XD) = -1926.46900245 A.U.
Imaginary frequency = -445.0282 cm^{-1}

6	2.862501	0.830362	-1.628957
7	2.997916	-0.285412	-0.875223
6	1.931595	-1.033541	-0.523490
6	1.774561	-1.899892	0.632178
6	0.619726	-2.671664	0.389149
6	0.108956	-3.553453	1.324883
6	0.782648	-3.678217	2.536764
6	1.930368	-2.932234	2.797698
6	2.428448	-2.037462	1.855242
6	0.894262	-1.412458	-1.504619
1	-0.788189	-4.124624	1.118661
1	3.317318	-1.465982	2.088362

6	4.370719	-0.461344	-0.351287
1	2.038128	0.849404	-2.329318
7	0.135881	-2.378038	-0.874897
1	4.352168	-0.407763	0.736714
8	0.731615	-1.035518	-2.656394
1	-0.670911	-2.799763	-1.304136
1	0.406318	-4.365286	3.285731
6	5.149170	0.748716	-0.924606
6	4.219032	1.352579	-1.990727
1	5.277305	1.472751	-0.117141
1	4.485002	1.005819	-2.995627
1	4.247865	2.443462	-2.005270
6	6.536709	0.345870	-1.428190
6	7.193605	-0.608093	-0.430023
6	6.403671	-1.931716	-0.308862
6	4.952371	-1.798401	-0.793787
1	8.227795	-0.810756	-0.716885
1	7.233350	-0.109135	0.544881
1	6.890454	-2.716081	-0.893703
1	6.409409	-2.269683	0.731373
1	4.906077	-1.850938	-1.886400
1	4.344919	-2.620872	-0.409101
1	7.146437	1.242333	-1.567200
1	6.459115	-0.137095	-2.409141
6	1.847277	2.044296	-0.314983
6	0.777918	1.241570	0.081804
1	1.633745	2.747751	-1.115804
1	0.720226	0.802595	1.067610
6	-0.392483	1.137550	-0.761569
8	-0.478851	1.678268	-1.864512
6	2.877663	2.486480	0.650196
6	3.613280	3.647749	0.395231
6	3.177138	1.751426	1.801667
6	4.637907	4.045089	1.244961
1	3.381424	4.240779	-0.483540
6	4.203003	2.144069	2.649859
1	2.615628	0.852476	2.031065
6	4.944238	3.288475	2.370842
1	5.199879	4.945820	1.025568
1	4.426945	1.552567	3.530484
1	5.748725	3.592026	3.030662
6	-6.966828	0.639555	0.821873
6	-7.406654	0.241556	-0.452957
6	-8.574369	0.788456	-0.990315
6	-9.262971	1.717998	-0.230796
6	-8.807767	2.105082	1.042701
6	-7.653150	1.574904	1.593236

6	-5.612579	-0.846897	-0.075576
1	-8.921073	0.488242	-1.972061
1	-10.172247	2.159866	-0.621496
1	-9.375199	2.836541	1.606386
1	-7.301710	1.872266	2.573557
7	-6.529622	-0.688921	-0.991174
7	-5.813791	-0.081925	1.035537
1	-5.212862	-0.045258	1.844075
6	-2.557593	-0.245217	-0.969172
6	-1.498270	0.289577	-0.259603
7	-2.583557	-0.942963	1.147405
7	-1.560429	-0.169600	1.020637
7	-3.187523	-1.010157	-0.053525
6	-2.994731	-0.138407	-2.385355
1	-4.030091	0.206107	-2.448718
1	-2.345384	0.562516	-2.902690
1	-2.932142	-1.112282	-2.878692
6	-4.427720	-1.752454	-0.197978
1	-4.430702	-2.516577	0.579890
1	-4.453010	-2.239032	-1.171301
1	2.441159	-3.044546	3.746525

TS-mn
E(RwB97XD) = -1926.47173342 A.U.
Imaginary frequency = -344.3953 cm^{-1}

6	1.473315	0.945299	-1.721135
7	2.258719	1.124334	-0.689725
6	3.165539	0.149419	-0.278338
6	4.019881	0.272170	0.913586
6	5.256102	-0.309208	0.592581
6	6.256488	-0.485206	1.532040
6	6.001869	-0.063632	2.835182
6	4.774067	0.495153	3.180281
6	3.770409	0.650717	2.226038
6	4.057766	-0.390287	-1.353810
1	7.201146	-0.942479	1.263173
1	2.806084	1.047756	2.517499
6	1.961387	2.376636	0.046791
1	1.674305	0.154346	-2.422520
7	5.253747	-0.673742	-0.750265
1	1.773930	2.122269	1.087534
8	3.803751	-0.554243	-2.535411
1	6.011062	-1.129681	-1.232464
1	6.768837	-0.184694	3.591330
6	0.661706	2.886160	-0.621813

6	0.574978	2.107073	-1.945781
1	-0.168047	2.578625	0.016721
1	0.950303	2.690419	-2.795681
1	-0.437388	1.787778	-2.200161
6	0.648766	4.410254	-0.759469
6	1.245892	5.049184	0.494884
6	2.736514	4.677268	0.659813
6	3.103440	3.376693	-0.073505
1	1.129823	6.134791	0.465507
1	0.676935	4.699166	1.363568
1	3.368760	5.480748	0.273734
1	2.973939	4.575375	1.722482
1	3.282107	3.573732	-1.135910
1	4.025320	2.955952	0.333243
1	-0.375890	4.753810	-0.922281
1	1.226497	4.715618	-1.639649
6	1.914830	-1.424395	0.184368
6	0.705159	-1.214738	-0.500421
1	1.883672	-1.099106	1.220222
1	0.444717	-1.753483	-1.400876
6	-0.260098	-0.346322	0.088360
8	-0.073461	0.277381	1.149866
6	2.757911	-2.625481	-0.027707
6	3.576709	-3.070499	1.014112
6	2.788050	-3.317876	-1.242181
6	4.407956	-4.171741	0.848603
1	3.564755	-2.539579	1.960593
6	3.616942	-4.418312	-1.408246
1	2.172614	-2.983574	-2.068895
6	4.432497	-4.848551	-0.364926
1	5.036997	-4.499437	1.668684
1	3.630120	-4.942063	-2.357609
1	5.080454	-5.707449	-0.498416
1	4.590444	0.801931	4.203041
6	-6.968070	-1.279457	0.160949
6	-7.265944	-0.068829	0.810993
6	-8.211995	-0.042664	1.838527
6	-8.828934	-1.233135	2.180705
6	-8.518638	-2.435312	1.519418
6	-7.584370	-2.482481	0.498584
6	-5.785088	0.390051	-0.653372
1	-8.447402	0.885447	2.345839
1	-9.566897	-1.244363	2.974417
1	-9.023756	-3.346987	1.816851
1	-7.344903	-3.407981	-0.010399
7	-6.501252	0.958746	0.278646
7	-6.015739	-0.948509	-0.776897

1	-5.554921	-1.571228	-1.421767
6	-2.584043	0.705686	-0.324405
6	-1.546325	-0.155616	-0.633601
7	-3.008533	-0.397675	-2.213149
7	-1.856767	-0.799841	-1.792809
7	-3.455583	0.520911	-1.339845
6	-2.823552	1.666899	0.785087
1	-3.800235	1.497210	1.245071
1	-2.039741	1.544860	1.527981
1	-2.802159	2.695407	0.412352
6	-4.779171	1.095593	-1.507466
1	-5.027667	1.010223	-2.565589
1	-4.759955	2.148877	-1.233305

TS-mx
E(RwB97XD) = -1926.46560304 A.U.
Imaginary frequency = -392.3288 cm^{-1}

6	1.381432	1.359617	-1.109114
7	2.651703	1.088838	-0.763866
6	3.250404	-0.081987	-1.131107
6	4.489083	-0.660618	-0.630896
6	4.839919	-1.681814	-1.538010
6	5.922932	-2.519356	-1.337135
6	6.688031	-2.330023	-0.189684
6	6.360283	-1.333655	0.724919
6	5.262923	-0.503667	0.516771
6	3.003380	-0.677287	-2.460434
1	6.163578	-3.296746	-2.052775
1	5.033085	0.248966	1.257342
6	3.112925	1.925186	0.363698
1	1.052934	1.027926	-2.082903
7	3.934816	-1.687152	-2.589000
1	3.385891	1.274102	1.193663
8	2.182583	-0.377150	-3.315764
1	4.004081	-2.255877	-3.416460
1	7.545045	-2.968617	-0.008937
6	1.868017	2.763897	0.738460
6	0.952147	2.651203	-0.487157
1	1.376140	2.271733	1.583430
1	1.121319	3.473015	-1.193393
1	-0.105712	2.651575	-0.226169
6	2.242450	4.188187	1.157508
6	3.525030	4.167829	1.990662
6	4.727790	3.669405	1.158595
6	4.295265	2.799647	-0.032560

1	3.734045	5.159630	2.397600
1	3.367767	3.506749	2.850531
1	5.298763	4.519786	0.777401
1	5.406862	3.099925	1.799906
1	3.989903	3.427616	-0.875900
1	5.127041	2.183940	-0.381800
1	1.418671	4.633661	1.720483
1	2.390464	4.813541	0.269605
6	1.437281	-1.357136	-0.185967
6	0.479733	-0.339213	-0.126339
1	1.344284	-2.055663	-1.011389
1	0.308705	0.171652	0.813692
6	-0.701121	-0.419066	-0.996697
8	-0.760433	-1.104342	-2.008303
6	2.200619	-1.838129	0.965992
6	2.907086	-3.043249	0.858887
6	2.285503	-1.134523	2.174901
6	3.664706	-3.528286	1.914774
1	2.862941	-3.597457	-0.073010
6	3.042884	-1.620064	3.231572
1	1.754766	-0.196720	2.298838
6	3.735384	-2.820540	3.109341
1	4.205252	-4.461571	1.802793
1	3.092020	-1.056706	4.156955
1	4.327359	-3.197201	3.935554
1	6.961441	-1.202770	1.616929
6	-7.165290	-0.451667	1.089474
6	-7.669093	-0.486960	-0.222688
6	-8.723804	-1.345385	-0.542831
6	-9.238574	-2.141530	0.464945
6	-8.721718	-2.093540	1.772514
6	-7.677370	-1.249716	2.110302
6	-6.093233	0.949644	-0.227926
1	-9.118244	-1.379606	-1.551470
1	-10.056806	-2.818029	0.247019
1	-9.152184	-2.733534	2.534001
1	-7.278993	-1.212596	3.116696
7	-6.969778	0.401665	-1.025655
7	-6.156261	0.484534	1.052586
1	-5.560275	0.765977	1.815168
6	-3.047608	0.619631	-1.287998
6	-1.864844	0.408499	-0.599015
7	-3.037284	1.743149	0.636767
7	-1.915553	1.115143	0.565537
7	-3.728213	1.459561	-0.483147
6	-3.576083	0.132245	-2.588697
1	-4.566405	-0.311581	-2.461118

1	-2.892330	-0.607274	-2.995990
1	-3.667393	0.960526	-3.296834
6	-5.080895	1.968302	-0.646671
1	-5.151781	2.873020	-0.042970
1	-5.248888	2.225098	-1.690843

8a
E(RwB97XD) = -1926.53705024 A.U.

6	3.511777	0.704639	0.781809
7	2.235968	1.420538	0.829312
6	1.183029	0.395517	0.835924
6	-0.167887	0.858216	0.326157
6	-1.044372	0.949177	1.409136
6	-2.358340	1.356877	1.265918
6	-2.802180	1.671164	-0.017008
6	-1.956179	1.557295	-1.114005
6	-0.633507	1.147416	-0.945515
6	0.876788	0.124695	2.334755
1	-3.023650	1.421263	2.118464
1	0.008075	1.039951	-1.811417
6	2.230794	2.441910	-0.237879
1	3.751869	0.309553	1.772037
7	-0.393407	0.552999	2.578854
1	1.668367	2.103318	-1.112517
8	1.636980	-0.356414	3.146780
1	-0.828962	0.481445	3.484652
1	-3.827323	1.994460	-0.157370
6	3.717225	2.613110	-0.664044
6	4.514388	1.744867	0.318138
1	3.826043	2.199198	-1.671680
1	4.844307	2.341330	1.173931
1	5.398518	1.290833	-0.135170
6	4.139195	4.080982	-0.720938
6	3.099619	4.902606	-1.486792
6	1.702013	4.839313	-0.820122
6	1.610710	3.745175	0.249946
1	3.431491	5.940129	-1.575423
1	3.040351	4.509575	-2.507820
1	1.457035	5.801506	-0.362469
1	0.938848	4.658983	-1.583971
1	2.134627	4.051568	1.161440
1	0.567447	3.572249	0.530712
1	5.119453	4.164499	-1.199704
1	4.253577	4.475674	0.295408
6	3.254130	-0.473674	-0.183670

6	1.835797	-0.900410	0.199287
1	3.224449	-0.085853	-1.206615
1	1.864130	-1.657859	0.984940
6	1.011386	-1.464275	-0.933351
8	1.316166	-1.340588	-2.103833
6	4.286420	-1.568236	-0.111959
6	5.073417	-1.873475	-1.221124
6	4.499376	-2.278028	1.073362
6	6.047861	-2.864634	-1.154615
1	4.918328	-1.330373	-2.147928
6	5.470638	-3.267899	1.143331
1	3.899264	-2.053690	1.950198
6	6.248962	-3.565439	0.028144
1	6.648650	-3.089087	-2.028953
1	5.621633	-3.808979	2.070799
1	7.006638	-4.338882	0.082777
1	-2.325092	1.784124	-2.106914
6	-5.622520	-0.170437	-0.725606
6	-5.447535	-0.250488	0.666783
6	-5.934363	0.718573	1.540736
6	-6.601306	1.788751	0.969442
6	-6.784194	1.886291	-0.422552
6	-6.305133	0.913573	-1.282286
6	-4.486628	-1.938234	-0.367753
1	-5.788475	0.645538	2.611419
1	-6.991679	2.571734	1.609033
1	-7.311534	2.743312	-0.824920
1	-6.439221	0.987431	-2.354896
7	-4.717715	-1.399557	0.863005
7	-5.008148	-1.246788	-1.345467
6	-1.382108	-2.346670	-1.274918
6	-0.241715	-2.118121	-0.526350
7	-1.692698	-2.919246	0.856994
7	-0.489805	-2.473613	0.765052
7	-2.242774	-2.851636	-0.371094
6	-1.729501	-2.089802	-2.695201
1	-1.875216	-3.031489	-3.231362
1	-0.917634	-1.544630	-3.170782
1	-2.654145	-1.511436	-2.760789
6	-3.653104	-3.167257	-0.546019
1	-3.807791	-3.565173	-1.546631
1	-3.903476	-3.935307	0.184874
1	-4.371743	-1.756174	1.739523

10a

E(RwB97XD) = -1926.52907475 A.U.

6	-2.794314	-0.173194	-1.685878
7	-1.896438	0.769495	-1.021338
6	-0.870185	-0.026355	-0.354192
6	-0.142118	0.632004	0.797378
6	1.184321	0.850713	0.425056
6	2.112777	1.412759	1.283740
6	1.684636	1.750840	2.565233
6	0.372115	1.523127	2.964414
6	-0.544980	0.955600	2.080425
6	0.280806	-0.185192	-1.392648
1	3.137011	1.577260	0.971423
1	-1.556428	0.766623	2.417729
6	-2.684976	1.752724	-0.259704
1	-2.312711	-0.537985	-2.595202
7	1.398135	0.405071	-0.882454
1	-2.743580	1.489282	0.801586
8	0.202346	-0.731627	-2.473780
1	2.327410	0.186528	-1.235608
1	2.390014	2.190520	3.260980
6	-4.119566	1.675130	-0.855390
6	-4.023204	0.662894	-2.006459
1	-4.780662	1.270682	-0.083942
1	-3.853931	1.184492	-2.953273
1	-4.928317	0.063707	-2.114844
6	-4.657442	3.048182	-1.254853
6	-4.471259	4.046840	-0.109863
6	-2.983612	4.192118	0.302886
6	-2.083729	3.149274	-0.368513
1	-4.884664	5.018871	-0.390776
1	-5.059499	3.697191	0.745683
1	-2.615942	5.190715	0.052531
1	-2.892067	4.095178	1.389632
1	-1.948320	3.381160	-1.429996
1	-1.087886	3.158364	0.084152
1	-5.716560	2.965639	-1.517418
1	-4.140691	3.405801	-2.153182
6	-2.919866	-1.408540	-0.728989
6	-1.558541	-1.422717	-0.019317
1	-2.990864	-2.302447	-1.351128
1	-1.649313	-1.484925	1.067161
6	-0.680846	-2.601580	-0.420268
8	-0.998152	-3.430853	-1.243769
6	-4.133346	-1.401091	0.178149
6	-5.338288	-1.900191	-0.324673
6	-4.134305	-0.891286	1.476104
6	-6.505390	-1.865078	0.427273

1	-5.359245	-2.320813	-1.325291
6	-5.297349	-0.856250	2.237268
1	-3.222035	-0.505274	1.913388
6	-6.490943	-1.337216	1.713974
1	-7.426363	-2.256409	0.009666
1	-5.267041	-0.450265	3.242147
1	-7.399020	-1.309449	2.305213
6	4.991561	0.706450	-0.750174
6	5.888122	0.649994	0.328298
6	6.742150	1.702765	0.648631
6	6.665452	2.827216	-0.154692
6	5.770726	2.900914	-1.237958
6	4.926491	1.850520	-1.549678
6	4.689289	-1.193868	0.184303
1	7.426513	1.646242	1.485897
1	7.308904	3.673171	0.056521
1	5.745004	3.802619	-1.838385
1	4.235520	1.906189	-2.382574
7	5.668644	-0.583684	0.901705
7	4.254344	-0.466426	-0.813751
6	1.869548	-2.881712	-0.380269
6	0.651644	-2.631189	0.221141
7	2.134488	-2.242935	1.739241
7	0.868760	-2.238819	1.506930
7	2.753136	-2.617876	0.602636
6	2.244432	-3.265794	-1.764172
1	2.662550	-4.275382	-1.786073
1	1.359203	-3.241134	-2.395972
1	2.985215	-2.568623	-2.161479
6	4.200045	-2.571243	0.528751
1	4.531429	-3.286902	-0.224281
1	4.590797	-2.889810	1.493751
1	0.059439	1.781135	3.968968
1	6.135983	-0.961293	1.710602

11a
E(RwB97XD) = -1926.52688526 A.U.

6	-0.650825	0.381868	1.147545
7	-1.799199	1.263642	0.889925
6	-2.940714	0.381039	0.627725
6	-4.181310	1.027018	0.052418
6	-5.192156	1.001835	1.014822
6	-6.458288	1.505454	0.776358
6	-6.712818	2.036260	-0.487255
6	-5.733861	2.036037	-1.474071

6	-4.464601	1.519407	-1.209377
6	-3.446416	-0.083486	2.024028
1	-7.226287	1.477088	1.539739
1	-3.721094	1.503066	-1.997069
6	-1.447692	2.227528	-0.174520
1	-0.628691	0.107932	2.200304
7	-4.721853	0.373848	2.169513
1	-1.951630	1.976435	-1.111930
8	-2.824842	-0.707384	2.858595
1	-5.267535	0.207447	3.000324
1	-7.695169	2.439721	-0.704059
6	0.079449	2.084852	-0.400104
6	0.554439	1.241663	0.783627
1	0.227160	1.515213	-1.322538
1	0.780095	1.889243	1.636819
1	1.455174	0.664183	0.571116
6	0.775473	3.437379	-0.560160
6	-0.048806	4.344482	-1.476009
6	-1.439897	4.652959	-0.872566
6	-1.843519	3.646033	0.214012
1	0.489910	5.273198	-1.680069
1	-0.167313	3.837494	-2.440427
1	-1.448201	5.658103	-0.441959
1	-2.191700	4.651501	-1.668102
1	-1.354154	3.887219	1.163854
1	-2.921144	3.688381	0.396906
1	1.780711	3.290673	-0.965667
1	0.899422	3.913696	0.419749
6	-2.342525	-0.764344	-0.232685
6	-0.938778	-0.956751	0.348227
1	-2.211392	-0.330804	-1.229023
1	-0.907232	-1.771168	1.070672
6	0.088394	-1.259367	-0.718567
8	-0.082650	-1.004787	-1.897063
6	-3.210285	-1.989978	-0.386194
6	-3.975855	-2.138342	-1.544020
6	-3.310357	-2.968675	0.605256
6	-4.819939	-3.229679	-1.711319
1	-3.907852	-1.387318	-2.324453
6	-4.152382	-4.062119	0.440509
1	-2.738751	-2.870634	1.520505
6	-4.910364	-4.197305	-0.717549
1	-5.404104	-3.324817	-2.619757
1	-4.215981	-4.811633	1.221479
1	-5.565467	-5.051688	-0.844661
1	-5.956993	2.431733	-2.457423
6	6.825996	0.112854	-0.418099

6	6.336162	0.291971	0.887526
6	6.668182	1.397615	1.667617
6	7.515753	2.329155	1.092656
6	8.015696	2.165942	-0.212219
6	7.680855	1.064427	-0.979697
6	5.579389	-1.569132	-0.013725
1	6.282566	1.522878	2.671860
1	7.801430	3.206442	1.661415
1	8.676361	2.921659	-0.620917
1	8.062950	0.935305	-1.985384
7	5.541165	-0.808444	1.117785
7	6.326423	-1.063413	-0.957494
6	2.532572	-2.042935	-0.953026
6	1.353823	-1.848058	-0.252023
7	2.757293	-2.648756	1.180788
7	1.550289	-2.229493	1.041525
7	3.361843	-2.548430	-0.020974
6	2.935666	-1.791219	-2.360212
1	3.095111	-2.737996	-2.883323
1	2.146706	-1.243062	-2.868307
1	3.867386	-1.222076	-2.398952
6	4.784405	-2.830817	-0.137498
1	4.988333	-3.288522	-1.103441
1	5.031189	-3.540657	0.651825
1	5.015184	-1.014964	1.952587

12a
E(RwB97XD) = -1926.52554231 A.U.

6	-0.733672	0.807896	1.167781
7	-2.053724	1.381798	0.916478
6	-3.019412	0.267507	1.001488
6	-4.254275	0.412704	0.142761
6	-5.345811	0.683594	0.968944
6	-6.623350	0.871173	0.471392
6	-6.799570	0.766037	-0.907179
6	-5.731541	0.466168	-1.745936
6	-4.453020	0.280840	-1.220377
6	-3.615293	0.369993	2.431896
1	-7.456769	1.081928	1.130385
1	-3.633177	0.018085	-1.877624
6	-1.987719	2.184065	-0.321816
1	-0.604687	0.609065	2.235598
7	-4.935457	0.690190	2.305193
1	-2.394455	1.636490	-1.176357
8	-3.014449	0.218931	3.473206

1	-5.545515	0.824502	3.095947
1	-7.789041	0.907659	-1.326499
6	-0.473913	2.427068	-0.584686
6	0.238562	1.847689	0.642732
1	-0.186522	1.840445	-1.463170
1	0.378474	2.620070	1.404810
1	1.218057	1.428216	0.404706
6	-0.165292	3.893666	-0.882677
6	-1.150269	4.441279	-1.918241
6	-2.617162	4.363962	-1.424382
6	-2.772885	3.481549	-0.179756
1	-0.890709	5.470876	-2.176998
1	-1.038254	3.854803	-2.836962
1	-2.992608	5.365022	-1.195969
1	-3.252956	3.972219	-2.224707
1	-2.412507	4.004317	0.712438
1	-3.828676	3.252549	-0.004424
1	0.862350	3.988207	-1.246126
1	-0.226156	4.482816	0.039869
6	-2.170438	-1.058816	0.855283
6	-0.797735	-0.549986	0.423011
1	-2.048653	-1.453576	1.868756
1	-0.760512	-0.346416	-0.648133
6	0.343091	-1.456305	0.808722
8	0.354893	-2.065979	1.862439
6	-2.803396	-2.141602	0.015684
6	-3.960028	-2.764779	0.492644
6	-2.301609	-2.536107	-1.222083
6	-4.611983	-3.733597	-0.254888
1	-4.359696	-2.477253	1.460785
6	-2.950050	-3.513250	-1.973606
1	-1.399490	-2.084590	-1.619675
6	-4.109218	-4.110176	-1.497599
1	-5.512150	-4.197983	0.131812
1	-2.543656	-3.805196	-2.935506
1	-4.615347	-4.867708	-2.084963
1	-5.892837	0.368195	-2.812615
6	7.138517	-0.198506	0.392608
6	6.558391	0.827709	-0.373420
6	6.953965	2.159282	-0.264482
6	7.959536	2.434222	0.646419
6	8.551224	1.419896	1.421314
6	8.152641	0.100023	1.306111
6	5.662669	-1.130737	-0.832194
1	6.498203	2.939689	-0.861238
1	8.299917	3.456244	0.766146
1	9.335113	1.682724	2.122062

1	8.605115	-0.684241	1.901318
7	5.615950	0.194148	-1.152201
7	6.549275	-1.415210	0.083230
6	2.715431	-2.122235	0.049381
6	1.480594	-1.516584	-0.121567
7	2.589893	-1.102088	-1.929165
7	1.462732	-0.909550	-1.342972
7	3.355834	-1.840422	-1.100111
6	3.318569	-2.908688	1.155682
1	3.398517	-3.961065	0.869255
1	2.684078	-2.838028	2.035305
1	4.321140	-2.543241	1.388895
6	4.737375	-2.125346	-1.458840
1	5.003079	-3.123730	-1.117179
1	4.792186	-2.095378	-2.546885
1	4.995519	0.620941	-1.822131
